# Synthesis and Characterization of Azido- and Nitratoalkyl Nitropyrazoles as Potential Melt-Cast Explosives

**DOI:** 10.3390/molecules28186489

**Published:** 2023-09-07

**Authors:** Elena Reinhardt, Tobias Lenz, Lukas Bauer, Jörg Stierstorfer, Thomas M. Klapötke

**Affiliations:** Department of Chemistry, Ludwig-Maximilians-University of Munich, Butenandtstr. 5–13, 81377 Munich, Germany; elrech@cup.uni-muenchen.de (E.R.); tolech@cup.uni-muenchen.de (T.L.); luauch@cup.uni-muenchen.de (L.B.)

**Keywords:** melt-cast explosives, nitropyrazoles, nitrate ester, azides, structure elucidation

## Abstract

Desirable advancements in the field of explosive materials include the development of novel melt-castable compounds with melting points ranging from 80 to 110 °C. This is particularly important due to the limited performance and high toxicity associated with TNT (trinitrotoluene). In this study, a series of innovative melt-castable explosives featuring nitratoalkyl and azidoalkyl functionalities attached to the 3-nitro-, 4-nitro-, 3,4-dinitropyrazole, or 3-azido-4-nitropyrazole scaffold are introduced. These compounds were synthesized using straightforward methods and thoroughly characterized using various analytical techniques, including single-crystal X-ray diffraction, IR spectroscopy, multinuclear nuclear magnetic resonance (NMR) spectroscopy, mass spectrometry, elemental analysis, and DTA. Furthermore, the energetic properties such as (theoretical) performance data, sensitivities, and compatibilities of the compounds were evaluated and compared among the different structures.

## 1. Introduction

Materials that store and release energy are essential for many applications. Energetic materials can store large amounts of energy and release it quickly when stimulated, whether as fuel for rockets and ammunitions or as explosives in the military or civilian sector [1,2,3]. In the mining industry, the invention of dynamite (nitroglycerin absorbed in kieselguhr) made explosive mining convenient. Today, cheap ammonium nitrate mixed with fuel oil, called ammonium nitrate fuel oil (ANFO), is used for safer handling. The initiation of relatively insensitive ANFO is typically performed by Pentolite, a mixture of pentaerythritol tetranitrate (PETN) that is embedded in a trinitrotoluene (TNT) melt. In the military sector, more robust, high-performance materials are needed. Mixtures of castable compounds such as TNT (Comp-B, Comp-C, Pentolite) or dinitroanisole (DNAN) (IMX-101) with high-performance materials such as hexogen (RDX), octogen (HMX), or PETN are commonly employed and comprise a significant category of military explosives [4,5]. In this procedure, the solid components of the compositions are agitated until they form a homogeneous dispersion within molten TNT. This mixture is then poured into molds or cavities and allowed to solidify as it cools. The inclusion of a melt-castable component, such as TNT, serves as a carrier material, facilitating the shaping of explosive compositions into desired forms and enhancing their loading capacity [5,6,7]. These traditional materials have several disadvantages that science is trying to solve with new technology and materials. TNT is toxic, and prolonged exposure causes anemia and harms the liver [8]. The production produces red wastewater which is harmful to the environment and disposal is, therefore, problematic [9]. The commonly used explosive RDX is found in air, water, and soil, especially around military facilities. RDX is proven to be toxic and harms the nervous system, urinary system, and prostate of animals. In addition, there is evidence of carcinogenic potential in animals [10,11]. Therefore, science is looking for more environmentally friendly alternatives with at best better detonation performance and lower sensitivity.

**Figure 1:**
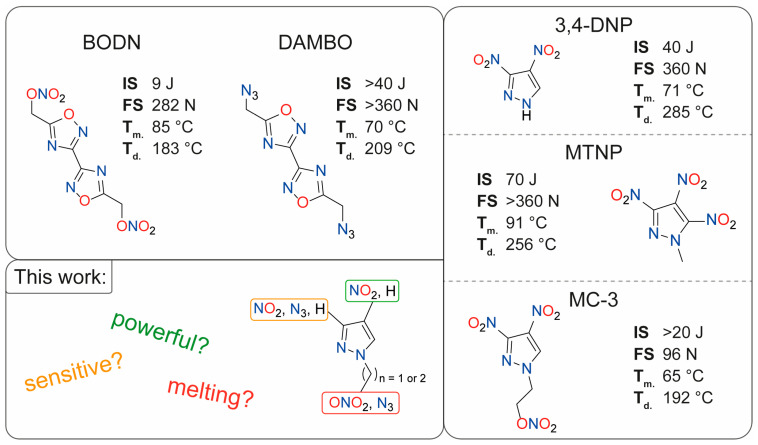
Current examples of energetic materials with suitable melting points based on azoles with bis(1,2,4-oxadiazole)bis(methylene) dinitrate (BODN) [16], diazidomethyl-bisoxadiazole (DAMBO) [17], 3,4-dinitro-1*H*-pyrazole (3,4-DNP) [18,19], 1-methyl-trinitropyrazole (MTNP) [20], and (MC-3) [15] and sensitivity toward impact and friction as well as their endo- (T_m._) or exothermic (T_d._) behavior under heating.

Recent work focusing on the replacement of RDX resulted in high-energy compounds such as CL-20 [12] or TKX-50 [13]. For TNT replacements, a combination of azoles with alkyl functionalities gained attention. Azido or nitrato groups combined with methyl or ethyl groups that are attached to the aromatic ring have proven to be a construction kit for acceptable powerful, low-sensitive, insoluble, and melting compounds [14]. The combinations were also evaluated by computational machine learning methods with good accuracy [15]. Examples of low-melting-point azoles are given in Figure 1.

The organic nitrate BODN [16] has gained attention due to its desirable properties and higher performance than TNT. The diazido-derivative DAMBO is melts 10 °C lower but has advanced stability and is insensitive. As a general trend, alkyl organic nitrates show acceptable densities and moderate sensitivities but lack thermal stability (typically < 190 °C). Whereas the corresponding azido derivatives show lower densities with better thermal stabilities [21,22,23,24]. Recently, nitropyrazoles gained attention as low-melting-point energetic materials (Figure 1). 3,4-DNP shows melting and late decomposition while being sensitive and powerful, but it is acidic and would react with metal shells as is known for picric acid. To overcome the acidity, N–H functionalization, for example, with methyl or nitratoethyl groups, can be performed. MTNP is the most powerful compound in Figure 1 with 8960 m s^−1^ detonation velocity, but is the least efficient to synthesize. Further research has focused on powerful pyrazoles through their combination with ortho azido/nitro groups [25], trinitromethyl moieties [26,27,28], or alkyl bridges [29,30,31], and also energetic coordination compounds [32] have been described. To study the effects of different explosophoric groups (nitro and azido), various chain lengths, and substitution patterns on pyrazoles concerning the properties of potential melt-cast explosives—including attributes such as melting temperature, decomposition temperature, density, and detonation parameters—we utilized the well-known 3-nitro-, 4-nitro, and 3,4-dinitropyrazoles as building blocks in this work, since compounds based on nitropyrazoles are reported as powerful energetic materials [33,34,35]. These nitropyrazoles were modified by replacing their acidic N–H position with alkyl azide and nitrate groups, thus customizing their properties to align with those of a melt-cast explosive. Additionally, we synthesized ortho azido/nitro compounds, resulting in substances with modified properties.

## 2. Results and Discussion

### 2.1. Synthesis

**Figure 2:**
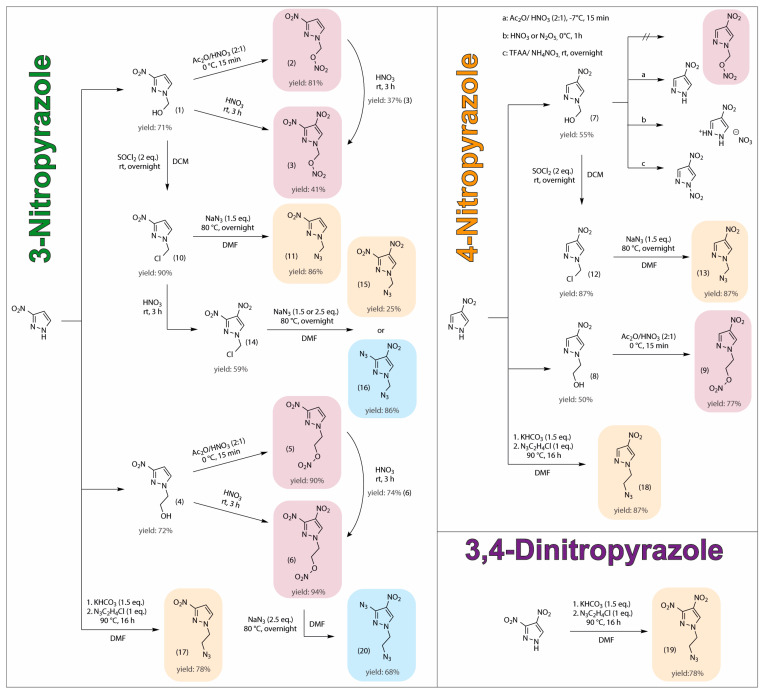
Synthesis overview of azido- and nitratoalkyl-nitropyrazoles.

The synthesis is divided into two sections: 1. synthesis of nitrate esters, and 2. synthesis of azides (Figure 2). The compounds with these functional groups were synthesized using 3-nitro- [19], 4-nitro- [36] and 3,4-dinitropyrazole [19] backbones, each equipped with either a methyl or an ethyl chain. In both cases, hydroxy moieties acted as the starting compounds. In accordance with literature procedures, 1-hydroxymethyl-3-nitropyrazole compounds (**1**) and 1-hydroxymethyl-4-nitropyrazole (**7**) were synthesized by reacting 3-nitropyrazole or 4-nitropyrazole with 40% formaldehyde solution [37]. For the hydroxyethyl groups, compounds (**4**) and (**8**), the nitropyrazole rings were deprotonated with potassium carbonate and further reacted with 2-bromoethanol [38].

Starting from 1-hydroxymethyl-3-nitropyrazole [37] (**1**) and 1-hydroxyethyl-3-nitropyrazole (**4**) [38], four different compounds were synthesized using different nitrating agents. Acetyl nitrate converted the hydroxy group to a nitrate ester with good yields within a few minutes (**2**: 81%, **5**: 90%). Nitration with fuming nitric acid for three hours led to the additional nitration of the 3-nitropyrazole ring at the C4 position. In this case, the yield of the hydroxyethyl compound (**4**) was significantly better (**6**: 94%) than for the hydroxymethyl compound (**1**) (**3**: 41%). An HPLC study showed that a large percentage of the nitratomethyl moiety was cleaved off during the nitration of OH group. Introducing the nitro group at the C4 position of the pyrazole ring enhances electron withdrawal from the pyrazole ring, leading to the cleavage of the nitratomethyl group. This phenomenon is evident in LC-MS measurements (see SI LC-MS measurements) and results in the lower yield of compound **3**. The cleavage of the nitratomethyl group was also observed at lower temperatures (−30 °C), resulting in approximately the same amount of reaction products. Additionally, compounds (**3**) and (**6**) were synthesized by reacting (**2**) and (**5**) with fuming nitric acid.

Replacing 3-nitropyrazole [19] with 4-nitropyrazole [36] enables successful reaction of the hydroxyethyl compound (**8**) with acetyl nitrate, yielding the desired nitratoethyl compound (**9**) with a satisfactory yield of 77%. However, attempts to nitrate the hydroxy group of the hydroxymethyl compound (**7**) were unsuccessful. As observed with compound (**3**), the nitratomethyl group formed in this reaction was cleaved off completely, precluding the formation of the desired product. During the reaction of compound (**7**) with acetyl nitrate, only 4-nitropyrazole could be isolated. Upon nitration with fuming nitric acid or N_2_O_5_, the major product obtained was the nitrate salt of 4-nitropyrazole. Using TFAA and NH_4_NO_3_ as the nitration system resulted not only in the cleavage of the nitratomethyl group but also in the nitration of the pyrazole ring at the *N1* position.

To synthesize the azidomethyl compounds, the hydroxymethyl compounds (**1**) and (**7**) were initially converted to 1-chloromethylnitropyrazoles [39] (**10**) and (**12**) using thionyl chloride with high yields of 90% and 87%, respectively. Both compounds were further subjected to a substitution reaction, resulting in the successful formation of the desired azidomethyl compounds (**11**) and (**13**) in a good yield (**11**: 86%, **13**: 87%). The attempted nitration of compound (**11**) at the C4 position of the pyrazole ring, using fuming nitric acid, resulted in the substitution of the azido group with a nitrato group, thereby regenerating compound (**3**) among other by-products. To circumvent this issue, the nitro group was first introduced by the previously described method prior to azide exchange, yielding compound **14**. Efforts to prepare 1-azidomethyl-3,4-dinitropyrazole through sodium azide substitution proved challenging. The typical use of 1.5 equivalents resulted in the substitution of the chlorine atom and also in an undesired substitution of the nitro group at the C3 position [30,40], leading to a mixture of compound **15** and the ortho azido/nitro compound **16**, which could be separated by column chromatography. In order to prevent substitution at the nitro group, the reaction was conducted with a precise amount of 1.0 equivalent of sodium azide. While this approach reduced the formation of compound **16**, the reaction remained incomplete and additional by-products were observed. Attempts to address this, by adjusting the reaction temperature or time, proved ineffective. However, increasing the equivalents to 2.5 resulted in the complete substitution of both the chlorine atom and nitro group at the C3 position, leading to full conversion to compound **16**. Upon attempting the conversion of (**6**) to 1-nitratoethyl-3-azido-4-nitropyrazole by replacing the nitro group at the C3 position with an azide moiety, an additional substitution of the nitrate ester with azide occurred, resulting in the formation of compound **20** in a good yield of 68%.

In the synthesis of azidoethyl compounds, the implementation of an azidoethyl transfer method [41] proved advantageous as this method allowed for the elimination of two reaction steps. The procedure involved the in situ deprotonation of nitropyrazoles, followed by their reaction with 1-azido-2-chloroethane (**17**, **18**) or 2-azidoethyl mesylate (**20**) at elevated temperatures using standardized conditions. These reactions were conducted in DMF as the solvent, and the resulting products could be efficiently isolated through extraction, giving satisfactory yields. If required, the products can be easily purified using column chromatography.

Three of the described compounds (**2**, **3**, **6**) were previously reported in the literature [15]. The synthesis of compound **2** closely followed established literature procedures. Compounds **3** and **6** were synthesized more efficiently, involving fewer steps, by nitration of the OH group and the subsequent introduction of the second nitro group to the pyrazole ring.

### 2.2. Crystal Structures

**Figure 3:**
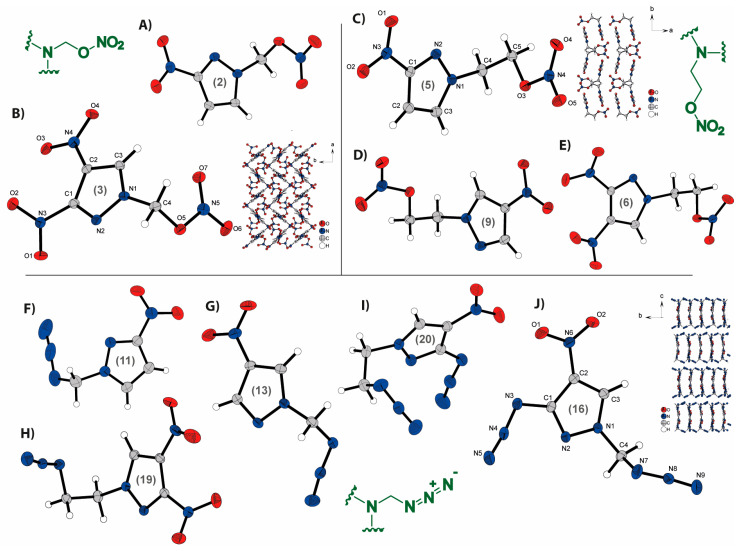
Molecular units of synthesized azido- and nitratoalkyl-nitropyrazoles: (**A**) 1-nitratomethyl-3-nitropyrazole (**2**); (**B**) 1-nitratomethyl-3,4-dinitropyrazole (**3**) and its extended structure; (**C**) 1-nitratethyl-3-nitropyrazole (**5**) and its extended structure; (**D**) 1-nitratoethyl-4-nitropyrazole (**9**); (**E**) 1-nitratoethyl-3,4-dinitropyrazole (**6**); (**F**) 1-azidomethyl-3-nitropyrazole (**11**); (**G**) 1-azidomethyl-4-nitropyrazole (**13**); (**H**) 1-azidoethyl-3,4-dinitropyrazole (**19**); (**I**) 1-azidoethyl-3-azido-4-nitropyrazole (**20**); (**J**) 1-azidomethyl-3-azido-4-nitropyrazole (**16**) and its extended structure.

Crystals of compounds **1**–**13**, **16**, **19**, and **20** were obtained either by recrystallization (**1**, **7**: chloroform; **2**, **3**, **5**: methanol; **4**: ether/dichloromethane; **6**, **20**: ethanol; **8**, **16**: ethyacetate, **20**: ether) or directly from the reaction mixture (**9**–**13**). The crystal structures of the energetic compounds are illustrated in Figure 3. The Supporting Information contains further information on the X-ray measurements and refinements, as well as crystal structure figures for compounds **1**, **4**, **7**, **8**, **10**, and **12**. Additional information on the X-ray structure determinations were deposited in the CCDC database [42], and the corresponding numbers are: **1**: 2255656, **2**: 2255417, **3**: 2255418, **4**: 2255410, **5**: 2255415, **6**: 2255419, **7**: 2255411, **8**: 2255658, **9**: 2255412, **10**: 2255416, **11**: 2255657, **12**: 2255409, **13**: 2255413, **16**: 2261896, **19**: 2255414, **20**: 2269934.

The non-hydrogen atoms’ ellipsoids in all structures are depicted at a 50% probability level.

The bond lengths of the pyrazole ring observed in all crystal structures (e.g., **3**: N6–C7 1.344(7), C7–C6 1.364(8); **9**: N1–C3 1.333(2), C2–C1 1.392(2); **11**: N1–N2 1.347(4), C2–C3 1.363(6)) are consistent with the typical range found in substituted pyrazoles [43]. In the mononitrated structures obtained, hydrogen bonds formed with neighboring CH elements cause the nitro group to adopt a nearly planar conformation within the pyrazole ring, regardless of its position at the 3- or 4-position (e.g., **2**: C2–C1–N3–O2 3.0°; **13**: C3–C2–N3–O2 –4.7°). However, when an additional nitro group is introduced at the 4-position of the 3-nitropyrazole, the nitro group at the 3-position undergoes a rotational displacement from the pyrazole plane, while the nitro group at the 4-position retains its planarity with the pyrazole ring (e.g., **3**: C3–C2–N4–O4 –6.5°, C2–C1–N3–O2 –30.6°). The analysis of the nitratomethyl compounds reveals that in both cases, the nitro group of the nitrate ester is positioned away from the pyrazole ring, forming a nearly planar arrangement with the side chain (**2**: C4–O3–N4–O5 –0.4°; **3**: C4–O5–N5–O7 –6.0°). In the nitratoethyl compounds, a gauche conformation is observed between the pyrazole ring and the nitrate ester (e.g., **6**: O5–C5–C4–N1 61.2°). A similar conformation is also observed in the azidoethyl compound. The azide group is nearly perpendicular to the pyrazole ring, resulting in a lower density. In contrast to the corresponding nitrate esters, the azidomethyl compounds have the azido group directed toward the pyrazole ring. However, in the azido-nitro compound **16**, the azido group is oriented in the opposite direction. Both, the nitro group and the azido group in these compounds lie in the same plane as the pyrazole ring (e.g., **16**: N4–N3–C1–N2 6.4° (azido); **20**: O1–N6–C2–C3 177.2° (nitro)), with the azido group pointing away from the nitro group due to electrostatic repulsion.

The mononitrated nitrate ester compounds (**2**, **5**, **9**) exhibit similar extended structures, as illustrated by the example of compound **5** in Figure 3C. These compounds form layered structures, where pyrazole rings are arranged in mirror symmetry, forming a unit within each layer. The side chains are positioned centrally within the layers. The extension of the side chains induces curvature in the layers, leading to a decrease in density (cf. **2**: 1.696 g/cm^3^, **5**: 1.629 g/cm^3^). Additionally, the nitro group at the C4 position instead of the C3 position results in an increased spacing between the layers (cf. **5**: 4.5433(14) Å, **9**: 7.6928 (18) Å), further contributing to the reduced density (cf. **5**: 1.629 g/cm^3^, **9**: 1.613 g/cm^3^). The dinitrated compounds **3** and **6** display zig-zag structures with acute angles, as exemplified by compound **3** in Figure 3B. This structural arrangement is more pronounced in the methyl derivative **3** than in the ethyl derivative **5**, which likely accounts for the increased sensitivity observed in compound **3** (**IS**: 4 J). The structural arrangements of the azide compounds (**11**, **13**, **19**) vary and do not exhibit comparable patterns. The dinitro compound (**19**), similar to nitric esters, exhibits a zig-zag structure. The extended structures of the azido-nitro (**16**, **20**) compounds demonstrate a rectangle formation, characterized by distinct orientations within each structure. (All the extended structures of energetic compounds are found in the Supplementary Information).

### 2.3. Physicochemical Properties

The physicochemical properties of the nitrate esters (**2**, **3**, **5**, **6**, **9**) and azides (**11**, **13**, **15–20**) have been systematically summarized in Table 1. The assessment of their response to external stimuli unveiled that the majority of the investigated compounds exhibit low sensitivity or insensitivity towards impact and friction. Exceptions were observed with the nitrate ester (**3**), which displayed an impact sensitivity value of 4 J, positioning it at the borderline of highly sensitive materials. The manifestation of the unexpectedly increased impact sensitivity for (**3**) becomes apparent upon checking the crystallographic arrangement, wherein distinct zig-zag motifs are prominently observed throughout the extended structure. Moreover, the electrostatic discharge (ESD) sensitivity of the compound was assessed, revealing a value of 51 mJ. This falls below the threshold of the electrostatic charge typically associated with a human being, underscoring the need for cautious handling measures. Moreover, the azido-nitro compounds (**19**) (*IS*: <1 J, *FS*: 10 N) and (**20**) (*IS*: 2 J, *FS*: 80 N) demonstrated sensitivities in the range of highly sensitive materials. This can be attributed to the presence of adjacent azido and nitro groups, which result in electrostatic repulsion.

The vast majority of the compounds are solids, with the exception of the azidomethyl (**15**) and azidoethyl (**17**, **18**) compounds. Substances with low melting points (**11**, **13**, **20**) undergo solidification under cooling conditions. In order to assess their melting and decomposition temperatures, differential thermal analysis (DTA) was performed, employing a controlled heating rate of 5 °C min^−1^. All solid-state compounds show melting points. It can be generally observed that the azides demonstrate lower melting points (between 40 and 50 °C) compared to their corresponding nitrate esters, while exhibiting around 15−20 °C higher decomposition temperatures (with the exception of **15**). The elongation of alkyl chains in both azides and nitrate esters results in higher decomposition temperatures. Consequently, the azidoethyl pyrazoles exhibit the highest decomposition temperatures (e.g., **19**: 216 °C). Among the five nitrate esters, three possess melting points within a suitable range (**2**: 70 °C, **3**: 93 °C, **5**: 78 °C), whereas the remaining two display slightly lower melting points of 61 (**6**) and 52 °C (**9**), respectively. It should be noted, only compound **5** shows a decomposition temperature exceeding 100 °C beyond its melting point (*T*_melt_: 78 °C, *T*_dec_: 198 °C). The substitution of a nitro group with an azido group on the pyrazole ring does not exhibit a consistent trend regarding thermal behavior.

The examination of the heat of formation and density reveals a general trend wherein the replacement of nitrate esters with azides and the shortening of alkyl chains lead to an increase in the heat of formation but a decrease in density. Accordingly, compounds containing two azido groups exhibit the highest heat of formation values (**16**: 755.8 kJ/mol, **20**: 707.4 kJ/mol). However, there are exceptions observed for the densities of certain pairs of compounds. Specifically, the presence of an additional azido group at the ring (see **15** and **16**) leads to increased density (**15**: 1.58 g/cm^3^, **16**: 1.670 g/cm^3^). The density significantly outweighs the heat of formation in influencing the detonation parameters. Consequently, despite their lower heat of formation, nitrate esters exhibit superior detonation velocities compared to azides. Among the compounds investigated, three of them exhibit detonation velocities around and above 8000 m/s (**3**, **6**, **16**). Compound **3** stands out, with a notable detonation velocity of 8668 m/s accompanied by a density of 1.848 g/cm^3^.

Compounds **3** and **5** were investigated for their compatibility with HMX and RDX based on their promising detonation velocity and/or the thermal behavior. Differential thermal analysis (DTA) was employed to evaluate their compatibility. The results revealed that compound **3** is compatible with HMX, showing minimal deviations in melting (−1 °C) and decomposition (+3 °C) temperatures. It exhibited moderate compatibility with RDX, with slight alterations in the thermal behavior (T*_melt_*: −5 °C; T*_dec_*: +5 °C). Compound **5** demonstrated moderate compatibility with RDX and HMX, as indicated by the observed temperature deviations. When combined with RDX, both the melting point and decomposition temperature exhibited a 6 °C shift. Similarly, slight temperature shifts were observed for **5** when mixed with HMX (T*_melt_*: −4 °C; T*_decom_*: +5 °C) (for additional information, see Supplementary Information).

### 2.4. NMR Spectroscopy

**Figure 4:**
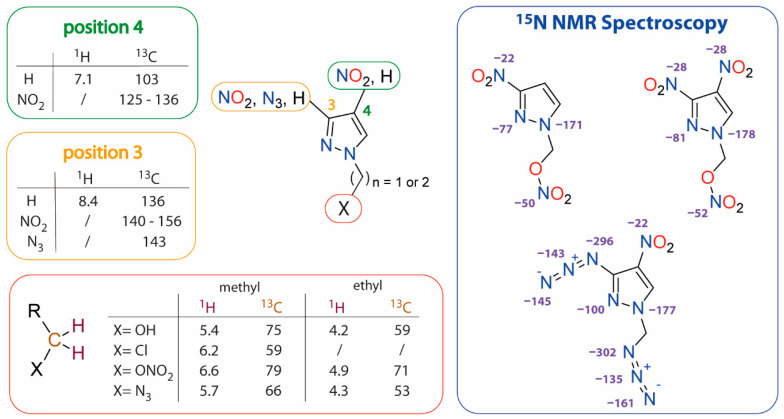
^1^H, ^13^C, and ^14,15^N NMR shifts (in ppm) for the synthesized compounds based on nitropyrazole.

The synthesized compounds were characterized using ^1^H, ^13^C, and, in selected cases, ^14^N NMR spectroscopy. Specifically, compounds **2**, **3**, and **16** were further characterized by ^15^N NMR spectroscopy. DMSO-*d*_6_, CDCl_3_ (for ^1^H, ^13^C and ^14^N NMR), and acetone-*d6* (for ^15^N NMR, due to slow decomposition in DMSO-*d*_6_) were employed as solvents for the measurements. A summary of the observed chemical shifts of ^1^H, ^13^C, and ^14,15^N chemical shifts is presented in Figure 4.

The assignment of ^1^H and ^13^C NMR signals is based on comparison with literature data [45,46]. The ^1^H and ^13^C chemical shifts provide valuable insights into the substitution pattern (-OH, -Cl, -ONO_2_, -N_3_) of the alkyl chain in the pyrazole moiety. The nitrate ester (**2**, **3**) shows the weakest shielding effect to the protons of the alkyl chain, resulting in a downfield shift (6.6 ppm), while the OH group (**1**, **7**) displays the strongest shielding effect, leading to an upfield shift (5.4 ppm). Substituting the nitrate ester with an azide (see **6** and **20**) causes a shift towards higher field (5.7 ppm). The same upfield shift is observed in the case of chlorine-azide exchange (-Cl: 6.2 ppm; -N_3_: 5.7 ppm). The influence of different substituents is more pronounced in compounds with a methyl group (**1**–**3**, **7**, **10**–**16**) compared to those with an ethyl moiety (**5**, **6**, **9**, **17**–**20**), although the overall trends remain consistent. Evidence of the substitution at the pyrazole ring can be derived from the observed ^1^H and ^13^C chemical shifts. The proton located at position 3 experiences a larger deshielding effect (**9**, **13**, **18**; 8.4 ppm) compared to that at position 4 (**2**, **5**, **11**, **17**; 7.1 ppm), attributable to its proximity to the pyrazole nitrogen atom. This trend is also reflected in the positions of the ^13^C NMR signals. Notably, nitration of one of the carbon atoms causes a downfield shift due to the electron-withdrawing effect of the nitro group. However, the presence of electron-withdrawing groups on the neighboring carbon atom mitigates this effect [47].

The assignment of the ^14^N and ^14,15^N NMR signals resulted from the evaluated coupling pattern of ^15^N NMR spectra and ^1^H ^15^N HMBC spectra (for additional information see Figures S6−S11 in the Supplementary Information). The ^15^N signal of the N1 atom in compounds **2**, **3**, and **16**, appears at about −175 ppm. In the disubstituted compounds (**3**, **16**), coupling of N1 with the ring proton at position 5 results in a doublet (^2^*J*_N,H_ = 2.8–3.0 Hz). In the case of the monosubstituted pyrazole (**2**), the N1 signal is split into a doublet of doublets (^2/3^*J*_N,H_ = 6.8, 4.4 Hz) due to coupling also with the second aromatic proton in position 4. The N2 nitrogen of the pyrazole ring exhibits a downfield shift compared to N1 (N1: about −171–177 ppm; N2: −77–100 ppm). Its ^15^N NMR signal is split into a triplet (^3^*J*_N,H_ = 2.6−2.8 Hz) due to the ^3^*J* coupling with the CH_2_ protons. The N2 NMR signal is shifted to higher field when the C3 position is occupied by an azide instead of a nitro group (e.g., **3**: −81 ppm, **16**: −100 ppm), as azides exert a weaker electron-withdrawing effect [46]. The assignment of the nitrogen atoms of the two azido groups was possible by analyzing the splitting pattern of ^15^N NMR signals and the ^1^H ^15^N HMBC spectrum (see Figures S10 and S11 in the Supplementary Information). The signals of the *α* and *β* nitrogen atoms of the azide group bonded to the alkyl chain display triplets (^2^*J*_N,H_ = 2.5 Hz; ^3^*J*_N,H_ = 5.0 Hz) resulting from coupling with the CH_2_ group. The other azido group (directly bonded to the pyrazole ring) shows singlet signals in the ^15^N NMR spectrum.

### 2.5. SSRT (Small-Scale Shock Reactivity Test)

**Figure 5:**
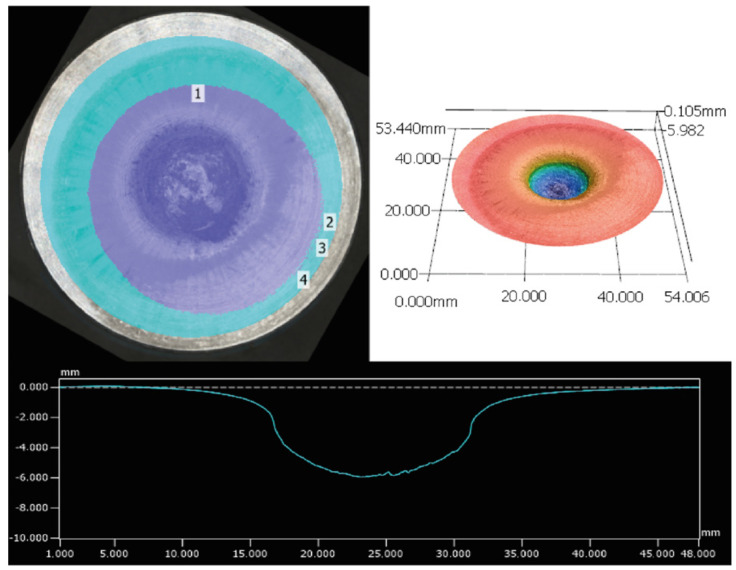
Measured area in cyan and indentations indicated in violet and numbered. Top right: 3D profile with color coded heights. Bottom: 2D profile measurement.

The SSRT test was established by Bohl et al. to measure the explosiveness of a compound on a smaller scale than similar tests such as the Trauzl lead block test [48]. The test setup consists of a steel block with a 7.5 mm wide borehole, which is placed on top of an aluminum witness block. The compound that is tested is filled into the borehole and pressed. To give comparable results with different compounds, the volume is constant and is set to 283 mm^3^ in order to account for the filling volume rather than comparing the same mass of energetic material with different filling heights. To ensure optimal energy transfer, the steel and aluminum blocks are placed into a fixating apparatus and a commercial detonator is then placed directly on the pressed compound. The detonation results in a dent in the aluminum witness block that has a characteristic volume for each energetic material that can be compared to each other [49,50]. The volume can be measured either by filling it with standardized sand, or by more technologically advanced optical topographic measurements with a profilometer (Figure 5).

The benefits of the profilometric measurements include, among others, more accurate and operator-independent values, time savings, and better comparability of values between working groups [49]. The compounds tested, 1-nitratomethyl-3,4-dinitropyrazole (**3**) and 1-nitratoethyl-3,4-dinitropyrazole (**6**), both showed good performance in the test setup (Table 2). Compared to established energetic materials, such as HMX or PETN, the methyl derivative (**3**) outperformed both, while the ethyl (**6**) derivative outperformed PETN. Although no single physicochemical value is measured with the SSRT test, valuable conclusions about the explosiveness and general strength of the compound can be drawn. The methyl derivative (**3**) showed a bigger dent volume than the ethyl derivative (**6**), which can be attributed to the increased density, detonation velocity, and detonation pressure.

## 3. Materials and Methods

All used materials, methods, and the detailed experimental part can be found in the Supplementary Information.

## 4. Conclusions

The objective of this research was to synthesize potential melt-castable explosives based on nitropyrazole and incorporating nitratoalkyl or azidoalkyl side chains, followed by a thorough comparison of their energetic characteristics. A total of 13 energetic materials were synthesized, consisting of five compounds containing nitratoalkyl moiety and eight with azidoalkyl moiety, where the alkyl moiety consisted of methyl or ethyl chains. The nitrate esters were obtained via nitration of the hydroxyl group, while the azides were synthesized through a chlorine-azide exchange reaction. Azidoethyl compounds were directly prepared using an azido ethyl transfer reagent and nitropyrazole. Among these compounds, ten display a solid-state nature with melting points, while the remaining compounds exist in a liquid state. In summary, the investigation reveals that the azido compounds possess superior thermal stability and exhibit higher heats of formation compared to their nitrate ester counterparts. However, this advantage comes at the expense of reduced melting points, leading some compounds to remain in the liquid state. Conversely, the nitrate ester compounds display higher densities, positively influencing their detonation parameters. Notably, compounds **3** and **6**, among others, stand out for their properties. Compound **3** exhibits a high detonation velocity (8668 m/s), while compound **6** demonstrates favorable thermal characteristics (*T*_melt_: 78 °C, *T*_dec_: 198 °C). Moreover, both compounds show good compatibility with HMX and RDX. Intriguingly, compound **3** outperforms the widely used PETN and HMX in the SSRT, indicating its potential as a highly energetic material.

## Figures and Tables

**Table 1:** Physicochemical properties and detonation parameters of all synthesized energetic compounds compared to TNT [44].

	*IS* ^a^ (J)	*FS* ^b^ (N)	*T*_melt_ ^c^ (°C)	T_exo._ ^d^ (°C)	*ρ* ^e^ (g/cm^3^)	Δ_f_*H*° ^f^ (kJ/mol)	*D*_C-J_ ^g^ (m/s)	*p*_C-J_ ^h^ (GPa)
(**2**)	>40	>360	70	161	1.696	−20.2	7675	24.3
(**3**)	4	288	93	159	1.848	8.7	8668	33.6
(**5**)	>40	>360	78	198	1.629	−67.1	7163	19.8
(**6**)	30	>360	61	198	1.734	−43.1	7932	26.7
(**9**)	>40	>360	52	191	1.613	−65.0	7110	19.5
(**11**)	>40	>360	40	179	1.513	401.8	6972	19.9
(**13**)	>40	>360	42	174	1.547	398.7	7100	19.8
(**15**) ^i^	>40	>360	/	154	1.58 ^j^	433.9	7679	23.1
(**17**) ^i^	>40	>360	/	214	1.28 ^j^	369.6	6235	11.9
(**18**) ^i^	>40	>360	/	214	1.34 ^j^	372.1	6483	13.3
(**19**)	25	>360	50	216	1.664	382.5	7639	22.9
(**16**)	<1	10	57	157	1.670	755.8	7945	24.4
(**20**)	2	80	41	172	1.554	707.4	7274	20.1
**TNT**	15	>360	81	289	1.65	−185	6950	20.5

^a^ Impact sensitivity (BAM drophammer, method 1 of 6); ^b^ friction sensitivity (BAM friction tester, method 1 of 6); ^c^ endothermic event (DTA, β = 5 °C∙min^−1^); ^d^ temperature of decomposition (DTA, β = 5 °C∙min^−1^); ^e^ density at 298 K recalculated from X-ray data; ^f^ heat of formation (calculated using the atomization method and CBS-4M enthalpies); ^g^ detonation velocity; ^h^ detonation pressure; ^i^ liquid-oily compounds; ^j^ volumetric determination.

**Table 2:** Results of SSRT for **3** and **6** compared to PETN and HMX.

	(3)	(6)	PETN	HMX
Dent volume	1216.36	1135.83	1107.81	1212.39
m (g) ^a^	496	447	478	511
*ρ* (g/cm^3^) ^b^	1.848	1.664	1.778	1.904
*P_CJ_* (kbar) ^c^	34.2	27.3	30.8	37.8
*V_det_* (m/s) ^d^	8734	8004	8429	9193

^a^ Filling mass; ^b^ theoretical max. density; ^c^ detonation pressure; ^d^ detonation velocity.

## Data Availability

The data presented in this study are available in the article and Supplementary Materials.

## Supplementary Materials

The following supporting information can be downloaded at: https://www.mdpi.com/article/10.3390/molecules28186489/s1. Schemes S1–S5: Synthesis of the presented compounds; Tables S1–S6: Crystallographic data and structure refinement details of the prepared compounds; Table S7: CBS-4M electronic enthalpies for atoms C, H, N and O and their literature values; Table S8: CBS-4M results and calculated gas-phase enthalpies; Tables S9–S11: Physicochemical properties and detonation parameter; Tables S12 and S13: Criteria and results for compatibility measurements; Table S14: Retention time and observed mass for different nitropyrazoles using LC-MS; Tables S15–S20: SSRT results; Table S21: NMR pulse sequences and parameters used for the characterization of the new compounds; Figure S1–S5: Crystal structures; Figure S6–S11: NMR spectra; Figure S12–S14: DTA spectra; Figures S15 and S16: Combined DTA-TGA spectra; Figures S17 and S18: DTA spectra of compatibility measurements; Figures S19 and S20: LC-MS monitoring of the nitration; Figure S21: Setup of the SSRT experiment. References [19,36,37,38,39,50,51,52,53,54,55,56,57,58,59,60,61,62,63,64,65,66,67,68,69,70,71,72] are cited in the Supplementary.
