# Supplementary material for: Synthesis and Characterization of Azido- and Nitratoalkyl Nitropyrazoles as Potential Melt-Cast Explosives
Source: Molecules. 2023 Sep 7;28(18):6489. doi: 10.3390/molecules28186489 (PMC10535347; doi:10.3390/molecules28186489)
Supplement: Supplementary file 1 [file molecules-28-06489-s001.zip › molecules-2578626-supplementary.pdf]

Supporting Information

Table of Content

1. Compound overview 1
2. X-ray diffraction.. 3
3. Heat of formation calculation.. 15
4. Physico-chemical properties 16
5. ^{15}NMR spectroscopy 18
6. Thermal stability 21
7. Compatibilities 25
8. LC-MS measurements.. 27
9. SSRT (small-scale shock reactivity test).. 30
10. Experimental part and general methods 34
11. References 44

1. Compound overview

The syntheses and individual compound numbers are shown in the schemes (**S1**–**S5**) below.

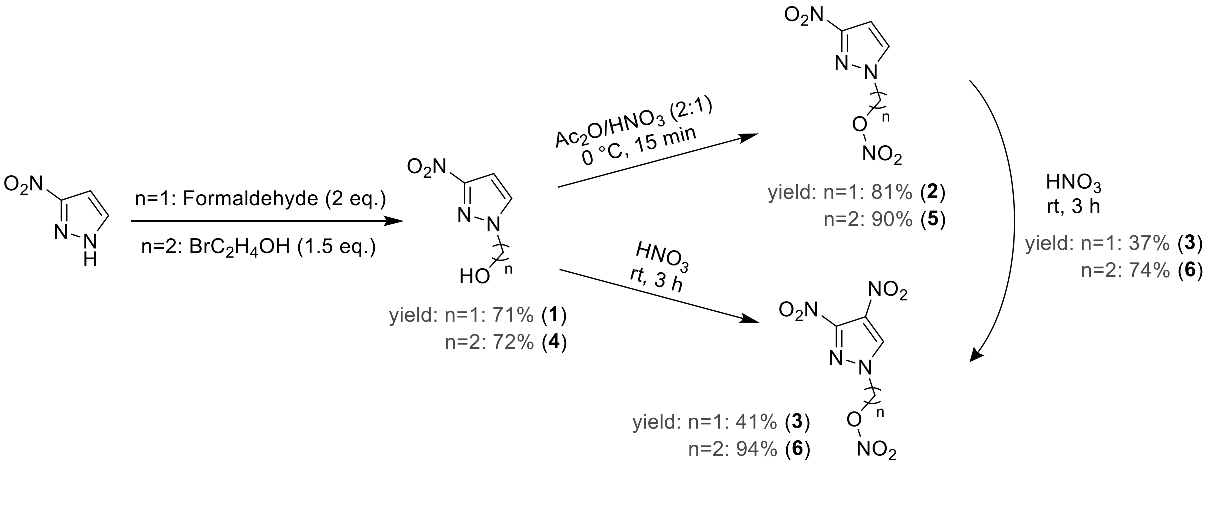

Scheme S1: Synthesis of 1-hydroxyalkyl and 1-nitratoalkyl-nitropyrazoles (**1**–**6**).

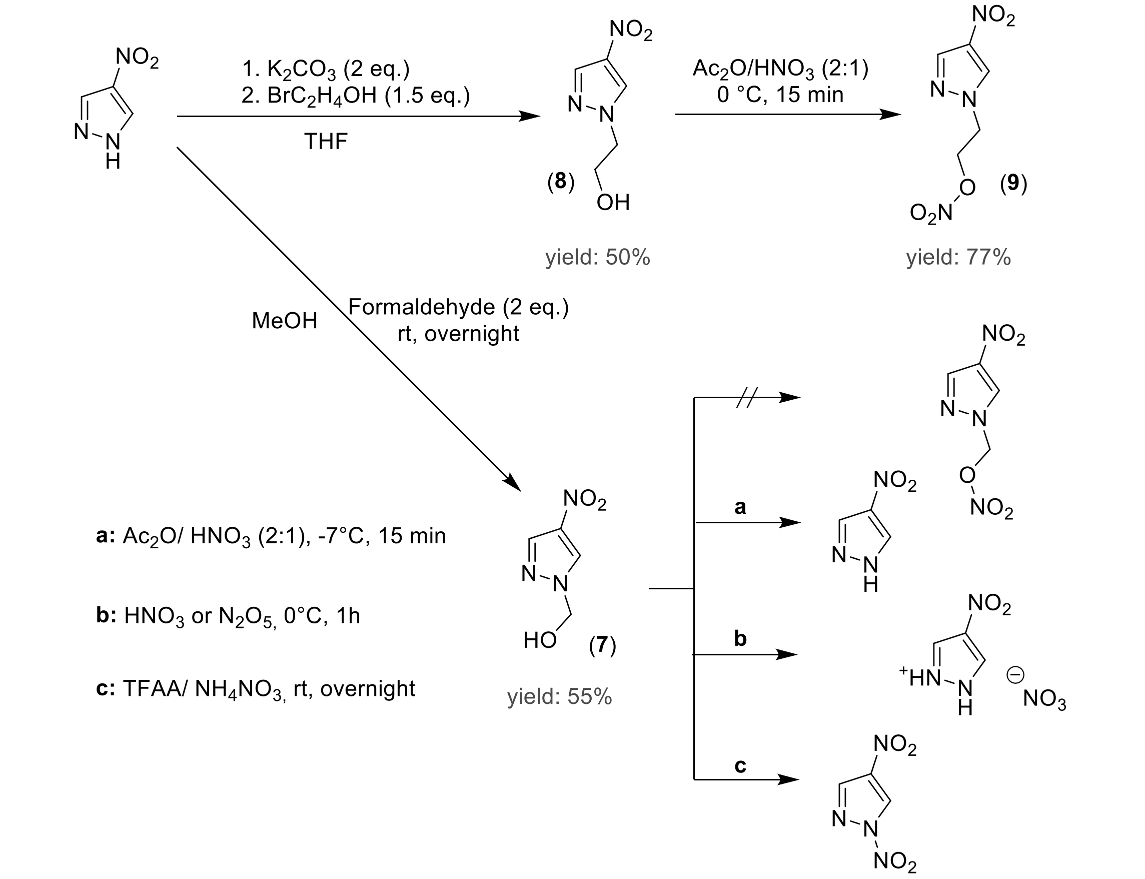

Scheme S2: Synthesis of 1-hydroxyalkyl and 1-nitratoalkyl-4-nitropyrazoles (**7**–**9**).

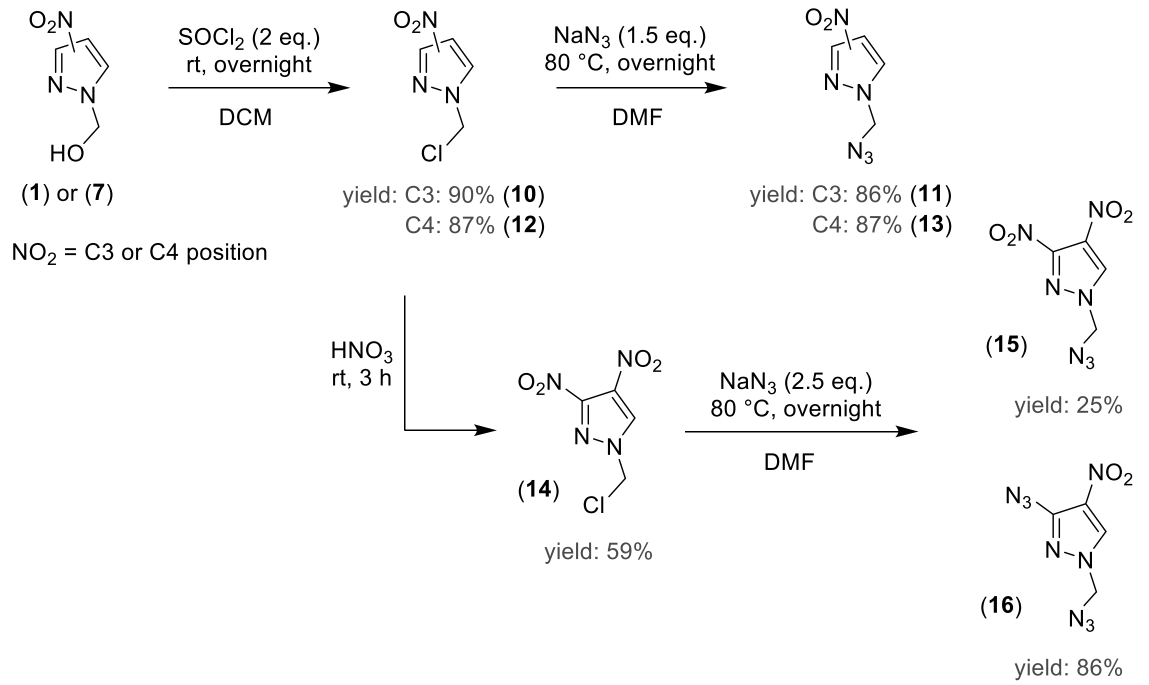

Scheme S3: Synthesis of 1-chloromethyl and 1-azidomethyl-nitropyrazoles (**10**–**15**) and 1-azidomethyl-3-azido-4-nitropyrazole (**16**).

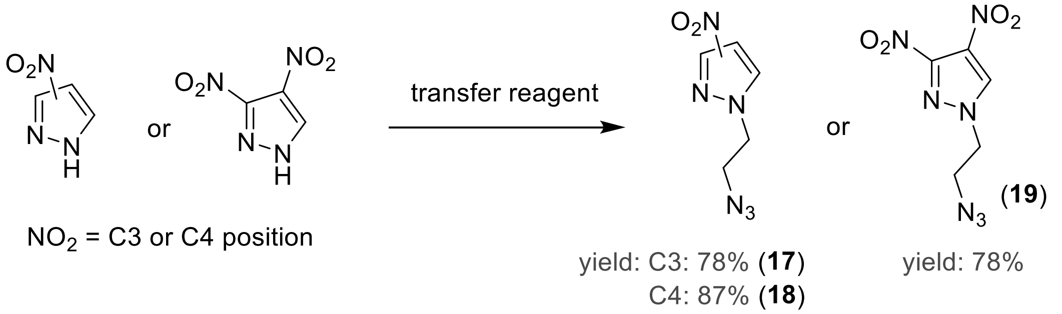

Scheme S4: Synthesis of 1-azidoethyl-nitropyrazoles (**17**–**19**) using an azido-ethyl-transfer reagent.

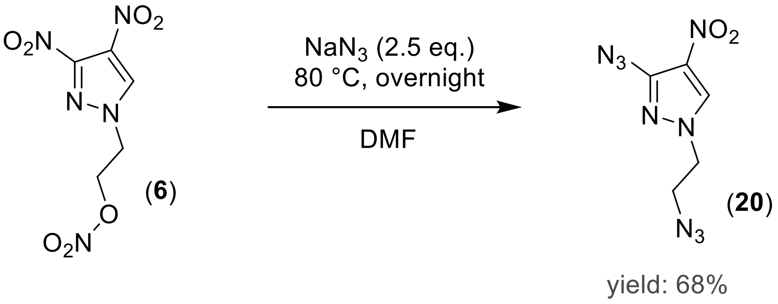

Scheme S5: Synthesis of 1-azidoethyl-3-azido-4-nitropyrazole (**20**).

2. X-ray diffraction

Crystalline compounds were measured either on an *Oxford Xcalibur3* diffractometer with a Spellman generator (voltage 50 kV, current 40 mA) and a Kappa CCD area for data collection using Mo-Kα radiation (λ = 0.71073 Å) or a *Bruker D8 Venture TXS* diffractometer equipped with a multilayer monochromator, a Photon 2 detector and a rotation-anode generator (Mo-Kα radiation). The data collection was performed using the CrysAlisPro software.[S1] The solution of the structure was performed by direct methods and refined by full-matrix least-squares on F2 (SHELXT)[S2] implemented in the OLEX2[S3] software suite. The non-hydrogen atoms were refined anisotropically and the hydrogen atoms were located and freely refined. The absorption correction was carried out by a SCALE3ABSPACK or SADABS Bruker Apex3 multiscan method.[S4,5] All DIAMOND2 plots are shown with thermal ellipsoids at the 50% probability level and hydrogen atoms are shown as small spheres of arbitrary radius.

Table S1: Crystallographic data and structure refinement details for the prepared compounds.

	**1HM3NPz (1)**	**1NM3NPz (2)**	**1NM34DNPz (3)**
Formula	$C_4H_5N_3O_3$	$C_4H_4N_4O_5$	$C_4H_3N_5O_7$
FW [g mol^{-1}]	143.11	188.11	233.11
Crystal system	triclinic	monoclinic	orthorhombic
Space group	*P*-1 (No. 2)	$P2_1/c$ (No. 14)	$Pna2_1$ (No. 33)
Color / Habit	colourless/ plate	colourless/ plates	colourless/ plates
Size [mm]	0.10 x 0.25 x 0.60	0.03 x 0.09 x 0.13	0.10 x 0.25 x 0.70
a [Å]	4.1175(5)	6.5096(3)	9.3876(11)
b [Å]	11.4525(17)	14.8639(7)	10.1407(10)
c [Å]	12.6534(14)	7.7489(4)	17.1149(18)
α [°]	94.378(10)	90	90
β [°]	93.410(9)	105.359(2)	90
γ [°]	96.875(11)	90	90
V [Å^3]	589.24(13)	722.99(6)	1629.3(3)
Z	4	4	8
$\rho_{calc.}$ [g cm^{-3}]	1.613	1.728	1.901
μ [mm^{-1}]	0.139	0.160	0.183
F(000)	296	384	944
$\lambda_{MoK\alpha}$ [Å]	0.71073	0.71073	0.71073
T [K]	105	173	105
θ Min-Max [°]	1.6, 26.4	3.5, 26.4	2.3, 26.4
Dataset	-5:5; -14:14; -15:15	-8: 8; -18:18; -8: 9	-11:7; -12:12; -21:21
Reflections collected	9297	12386	5923
Independent refl.	2414	1472	2995
R_{int}	0.056	0.034	0.047
Observed reflections	1872	1291	2236
Parameters	221	134	306
R_1 (obs)[a]	0.0499	0.0365	0.0500
wR_2 (all data)[b]	0.1327	0.1023	0.1047
S [c]	1.06	1.21	1.02
Resd. dens [e Å^{-3}]	-0.28, 0.29	-0.24, 0.21	-0.23, 0.34
Device type	Oxford Xcalibur3	D8 Venture	Oxford Xcalibur3
Solution	SHELXT	SHELXT	SIR-92
Refinement	SHELXL-2018	SHELXL-2018	SHELXL-2018
Absorption correction	multi-scan	multi-scan	multi-scan
CCDC	2255656	2255417	2255418

[a]$R_1 = \Sigma||F_0|-|F_c||/\Sigma|F_0|$; [b]$wR_2 = [\Sigma[w(F_0^2-F_c^2)^2]/\Sigma[w(F_0)^2]]^{1/2}$; $w = [\sigma c^2(F_0^2)+(xP)^2+yP]^{-1}$ and $P=(F_0^2+2F_c^2)/3$; [c]$S = \{\Sigma[w(F_o^2-F_c^2)^2]/(n-p)\}^{1/2}$ (n = number of reflections; p = total number of parameters).

Table S2: Crystallographic data and structure refinement details for the prepared compounds.

	**1HE3NPz (4)**	**1NE3NPz (5)**	**1NE34DNPz (6)**
Formula	$C_5H_7N_3O_3$	$C_5H_6N_4O_5$	$C_5H_5N_5O_7$
FW [g mol^{-1}]	157.14	202.14	247.14
Crystal system	monoclinic	monoclinic	orthorhombic
Space group	$P2_1/c$ (No. 14)	$P2_1/c$ (No. 14)	$P2_12_12_1$ (No. 19)
Color / Habit	colourless/ block	colourless/ block	colourless/ block
Size [mm]	0.04 x 0.08 x 0.16	0.25 x 0.50 x 0.50	0.03 x 0.06 x 0.12
a [Å]	4.4397(2)	7.1230(11)	8.2650(2)
b [Å]	11.8945(5)	14.488(3)	8.9959(2)
c [Å]	13.0629(6)	7.9349(10)	12.4975(3)
α [°]	90	90	90
β [°]	91.211(2)	102.586(13)	90
γ [°]	90	90	90
V [Å^3]	689.67(5)	799.2(2)	929.20(4)
Z	4	4	4
$\rho_{calc.}$ [g cm^{-3}]	1.513	1.680	1.767
μ [mm^{-1}]	0.127	0.151	0.166
F(000)	328	416	504
$\lambda_{MoK\alpha}$ [Å]	0.71073	0.71073	0.71073
T [K]	173	91	173
θ Min-Max [°]	3.1, 26.4	2.8, 26.4	3.0, 26.4
Dataset	-5: 5; -14: 14; -16: 16	-8: 8; -16: 18; -9: 7	-10:10;-11:11;-15:15
Reflections collected	14822	3676	21075
Independent refl.	1410	1627	1897
R_{int}	0.042	0.027	0.033
Observed reflections	1225	1271	1845
Parameters	128	151	174
R_1 (obs)[a]	0.0339	0.0371	0.0239
wR_2 (all data)[b]	0.0874	0.0881	0.0663
S [c]	1.13	1.05	1.16
Resd. dens [e Å^{-3}]	-0.24, 0.18	-0.23, 0.19	-0.17, 0.17
Device type	D8 Venture	Oxford Xcalibur3	D8 Venture
Solution	SHELXT	SHELXT	SHELXT
Refinement	SHELXL-2018	SHELXL-2018	SHELXL-2018
Absorption correction	multi-scan	multi-scan	multi-scan
CCDC	2255410	2255415	2255419

[a]$R_1 = \Sigma||F_0|-|F_c||/\Sigma|F_0|$; [b]$wR_2 = [\Sigma[w(F_0^2-F_c^2)^2]/\Sigma[w(F_0)^2]]^{1/2}$; $w = [\sigma c^2(F_0^2)+(xP)^2+yP]^{-1}$ and $P=(F_0^2+2F_c^2)/3$; [c]$S = \{\Sigma[w(F_o^2-F_c^2)^2]/(n-p)\}^{1/2}$ (n = number of reflections; p = total number of parameters).

Table S3: Crystallographic data and structure refinement details for the prepared compounds.

	**1HM4NPz (7)**	**1HE4NPz (8)**	**1NE4NPz (9)**
Formula	$C_4H_5N_3O_3$	$C_5H_7N_3O_3$	$C_5H_6N_4O_5$
FW [g mol^{-1}]	143.11	157.14	202.14
Crystal system	monoclinic	monoclinic	monoclinic
Space group	*C*2/*c* (No. 15)	P2$_1$/*c* (No. 14)	*P*2$_1$/*c* (No. 14)
Color / Habit	colourless/ needle	colourless/ block	colourless/ plates
Size [mm]	0.02 x 0.06 x 0.40	0.06 x 0.08 x 0.16	0.01 x 0.04 x 0.04
a [Å]	21.5247(15)	7.6570(2)	9.8174(5)
b [Å]	4.1038(4)	8.6786(3)	8.3784(4)
c [Å]	12.9782(10)	10.3956(3)	10.9893(6)
α [°]	90	90	90
β [°]	92.166(7)	92.928(1)	115.320(2)
γ [°]	90	90	90
V [Å^3]	1145.59(16)	689.91(4)	817.08(7)
Z	8	4	4
$\rho_{calc.}$ [g cm^{-3}]	1.660	1.513	1.643
μ [mm^{-1}]	0.143	0.126	0.148
F(000)	592	328	416
$\lambda_{MoK\alpha}$ [Å]	0.71073	0.71073	0.71073
T [K]	103	173	173
θ Min-Max [°]	3.1, 26.4	3.1, 26.4	3.2, 26.4
Dataset	-26: 26; -5: 5; -16: 16	-9: 9; -10: 10; -12: 12	-12:12;-10:10;-13:13
Reflections collected	7584	14652	14361
Independent refl.	1169	1405	1671
R_{int}	0.038	0.035	0.050
Observed reflections	990	1294	1379
Parameters	111	128	151
R_1 (obs)[a]	0.0298	0.0338	0.0355
wR_2 (all data)[b]	0.0781	0.0897	0.0897
S [c]	1.05	1.11	1.13
Resd. dens [e Å^{-3}]	-0.17, 0.24	-0.21, 0.22	-0.23, 0.16
Device type	Oxford Xcalibur3	D8 Venture	D8 Venture
Solution	SHELXT	SHELXT	SHELXT
Refinement	SHELXL-2018	SHELXL-2018	SHELXL-2018
Absorption correction	multi-scan	multi-scan	multi-scan
CCDC	2255411	2255658	2255412

[a]$R_1 = \Sigma||F_0|-|F_c||/\Sigma|F_0|$; [b]$wR_2 = [\Sigma[w(F_0^2-F_c^2)^2]/\Sigma[w(F_0)^2]]^{1/2}$; $w = [\sigma c^2(F_0^2)+(xP)^2+yP]^{-1}$ and $P=(F_0^2+2F_c^2)/3$; [c]$S = \{\Sigma[w(F_o^2-F_c^2)^2]/(n-p)\}^{1/2}$ (n = number of reflections; p = total number of parameters).

Table S4: Crystallographic data and structure refinement details for the prepared compounds.

	**1ClM3NPz (10)**	**1AM3NPz (11)**	**1ClM4NPz (12)**
Formula	$C_4H_4ClN_3O_2$	$C_4H_4N_6O_2$	$C_4H_4ClN_3O_2$
FW [g mol^{-1}]	161.55	168.13	161.55
Crystal system	orthorhombic	orthorhombic	monoclinic
Space group	*Pbca* (No. 61)	$Pna2_1$ (No. 33)	$P2_1/c$ (No. 14)
Color / Habit	colourless/ block	colourless/ plate	colourless/ needle
Size [mm]	0.03 x 0.05 x 0.10	0.01 x 0.04 x 0.08	0.10 x 0.10 x 0.45
a [Å]	6.7667(2)	8.2802(9)	4.2774(4)
b [Å]	12.9917(4)	14.2417(16)	21.208(3)
c [Å]	15.0807(4)	6.1449(8)	7.2323(7)
α [°]	90	90	90
β [°]	90	90	97.183(9)
γ [°]	90	90	90
V [Å^3]	1325.76(7)	724.63(15)	650.93(13)
Z	8	4	4
$\rho_{calc.}$ [g cm^{-3}]	1.619	1.541	1.648
μ [mm^{-1}]	0.513	0.128	0.523
F(000)	656	344	328
$\lambda_{MoK\alpha}$ [Å]	0.71073	0.71073	0.71073
T [K]	173	173	93
θ Min-Max [°]	3.1, 26.4	3.6, 26.3	1.9, 26.4
Dataset	-8: 8; -16: 16; -18: 18	-10: 10; -17: 17; -7: 7	-4: 5; -26: 25; -8: 9
Reflections collected	21917	12637	2886
Independent refl.	1357	1480	1329
R_{int}	0.041	0.102	0.032
Observed reflections	1236	1085	935
Parameters	107	125	107
R_1 (obs)[a]	0.0366	0.0447	0.0458
wR_2 (all data)[b]	0.0924	0.0894	0.0996
S [c]	1.07	1.07	1.06
Resd. dens [e Å^{-3}]	-0.42, 0.37	-0.16, 0.15	-0.22, 0.28
Device type	D8 Venture	D8 Venture	Oxford Xcalibur3
Solution	SHELXT	SHELXT	SHELXT
Refinement	SHELXL-2018	SHELXL-2018	SHELXL-2018
Absorption correction	multi-scan	multi-scan	multi-scan
CCDC	2255416	2255657	2255409

[a]$R_1 = \Sigma||F_0|-|F_c||/\Sigma|F_0|$; [b]$wR_2 = [\Sigma[w(F_0^2-F_c^2)^2]/\Sigma[w(F_0)^2]]^{1/2}$; $w = [\sigma c^2(F_0^2)+(xP)^2+yP]^{-1}$ and $P=(F_0^2+2F_c^2)/3$; [c]$S = \{\Sigma[w(F_o^2-F_c^2)^2]/(n-p)\}^{1/2}$ (n = number of reflections; p = total number of parameters).

Table S5: Crystallographic data and structure refinement details for the prepared compounds.

	**1AM4NPz (13)**	**1AM3A4NPz(16)**	**1AE34DNPz(19)**
Formula	$C_4H_4N_6O_2$	$C_4H_3N_9O_2$	$C_5H_5N_7O_4$
FW [g mol^{-1}]	168.13	209.15	227.16
Crystal system	monoclinic	orthorhombic	monoclinic
Space group	*C*2/*c* (No. 15)	*Pbca* (No. 61)	$P2_1$ (No. 4)
Color / Habit	colourless/ block	yellow/ plate	colourless/ plate
Size [mm]	0.30 x 0.40 x 0.50	0.03 x 0.10 x 0.16	0.18 x 0.16 x 0.04
a [Å]	6.4339(9)	12.3345(10)	8.120(2)
b [Å]	11.4490(14)	6.5734(6)	13.319(3)
c [Å]	19.124(2)	20.1402(17)	8.235(2)
α [°]	90	90	90
β [°]	93.367(10)	90	91.83(3)
γ [°]	90	90	90
V [Å^3]	1406.3(3)	1633.0(2)	890.1(1)
Z	8	8	4
$\rho_{calc.}$ [g cm^{-3}]	1.588	1.702	1.695
μ [mm^{-1}]	0.131	0.142	0.147
F(000)	688	848	464
$\lambda_{MoK\alpha}$ [Å]	0.71073	0.71073	0.71073
T [K]	123	173	173
θ Min-Max [°]	2.1, 26.4	3.7, 26.4	2.9, 28.2
Dataset	-8: 6; -14: 13; -21: 23	-15:15; -8:8; -25:25	-10:10;-17:17;-10:10
Reflections collected	2960	27146	18061
Independent refl.	1432	1662	4396
R_{int}	0.025	0.067	0.0432
Observed reflections	1067	1526	4122
Parameters	125	148	330
R_1 (obs)[a]	0.0392	0.0400	0.0327
wR_2 (all data)[b]	0.0909	0.1037	0.0819
S [c]	1.02	1.20	1.076
Resd. dens [e Å^{-3}]	-0.21, 0.20	-0.30, 0.28	-0.238, 0.144
Device type	Oxford Xcalibur3	D8 Venture	D8 Venture
Solution	SHELXT	SHELXT	SHELXT
Refinement	SHELXL-2018	SHELXL-2018	SHELXL-2018
Absorption correction	multi-scan	multi-scan	multi-scan
CCDC	2255413	2261896	2255414

[a]$R_1 = \Sigma||F_0|-|F_c||/\Sigma|F_0|$; [b]$wR_2 = [\Sigma[w(F_0^2-F_c^2)^2]/\Sigma[w(F_0)^2]]^{1/2}$; $w = [\sigma c^2(F_0^2)+(xP)^2+yP]^{-1}$ and $P=(F_0^2+2F_c^2)/3$; [c]$S = \{\Sigma[w(F_o^2-F_c^2)^2]/(n-p)\}^{1/2}$ (n = number of reflections; p = total number of parameters).

Table S6: Crystallographic data and structure refinement details for the prepared compounds.

	**1AE3A4NPz (20)**
Formula	$C_5H_5N_9O_2$
FW [g mol^{-1}]	223.18
Crystal system	monoclinic
Space group	$P2_1/c$ (No. 14)
Color / Habit	colourless/ block
Size [mm]	0.08 x 0.12 x 0.18
a [Å]	10.8862(5)
b [Å]	12.1490(6)
c [Å]	7.0825(4)
α [°]	90
β [°]	91.354(2)
γ [°]	90
V [Å^3]	936.45(8)
Z	4
$\rho_{calc.}$ [g cm^{-3}]	1.583
μ [mm^{-1}]	0.129
F(000)	456
$\lambda_{MoK\alpha}$ [Å]	0.71073
T [K]	173
θ Min-Max [°]	3.3, 26.4
Dataset	-13:13; -15:15; -8:8
Reflections collected	16166
Independent refl.	1921
R_{int}	0.041
Observed reflections	1657
Parameters	165
R_1 (obs)[a]	0.0378
wR_2 (all data)[b]	0.0970
S [c]	1.12
Resd. dens [e Å^{-3}]	-0.22, 0.22
Device type	D8 Venture
Solution	SIR-92
Refinement	SHELXL-2018
Absorption correction	multi-scan
CCDC	2269934

[a]$R_1 = \Sigma||F_0|-|F_c||/\Sigma|F_0|$; [b]$wR_2 = [\Sigma[w(F_0^2-F_c^2)^2]/\Sigma[w(F_0)^2]]^{1/2}$; $w = [\sigma c^2(F_0^2)+(xP)^2+yP]^{-1}$ and $P=(F_0^2+2F_c^2)/3$; [c]$S = \{\Sigma[w(F_o^2-F_c^2)^2]/(n-p)\}^{1/2}$ (n = number of reflections; p = total number of parameters).

Compound **2** and **3** can be crystallized from methanol, forming colourless platelets. They crystallized in common space groups (**2**: $P2_1/c$; **3**: $Pna2_1$) and have four and eight molecules per unit cell, with a cell volume of 722.99(6) Å^3 and 1629.3(3) Å^3, respectively. At 173 K and 105 K, the calculated densities are 1.728 g cm^{-3} and 1.901 g cm^{-3}, respectively. Molecular units and extended structures are illustrated in **Figure S1**.

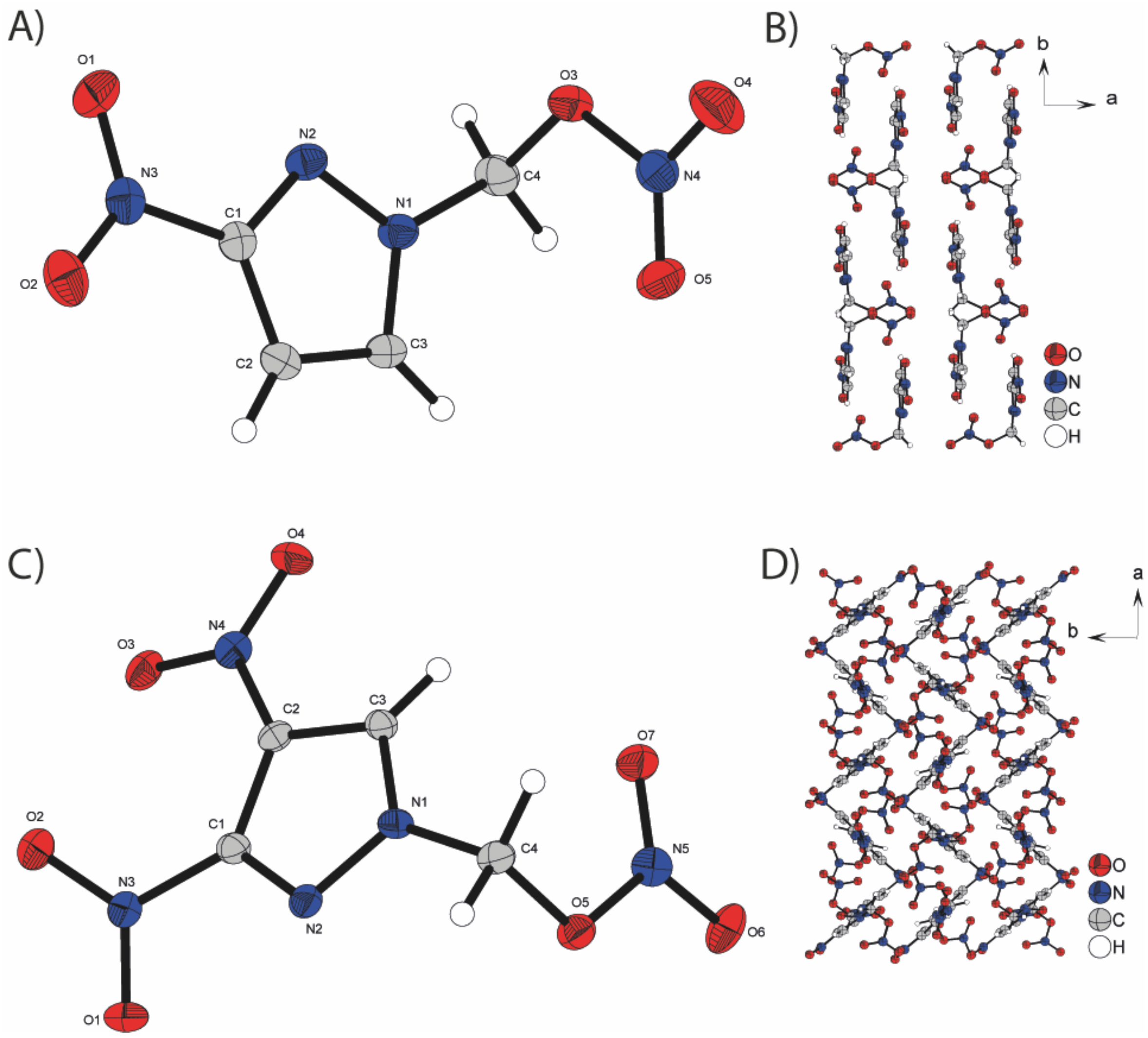

Figure S1: Crystal structures of 1-nitratomethyl-nitropyrazole compounds: **A**, **B**) 1-nitratomethyl-3-nitropyrazole (**2**); **C**, **D**) 1-nitratomethyl-3,4-dinitropyrazole (**3**).

Compound **5**, **9** and **6** can be crystallized from methanol or ethanol forming colourless platelets (**9**) or blocks (**5**, **6**). Compounds **5** and **9** have four molecules per unit cell and share common space groups $P2_1/c$. Their cell volumes are 799.2(2) Å^3 and 817.08(7) Å^3, while the calculated densities at 91 K and 173 K are 1.680 g cm^{-3} and 1.643 g cm^{-3}, in that order. Compound **6** crystallized in the space group $P2_12_12_1$ with four molecules per unit cell and a cell volume of 929.20(4) Å^3. At 173 K, the calculated density is 1.767 g cm^{-3}. Molecular units and extended structures are illustrated in **Figure S2**.

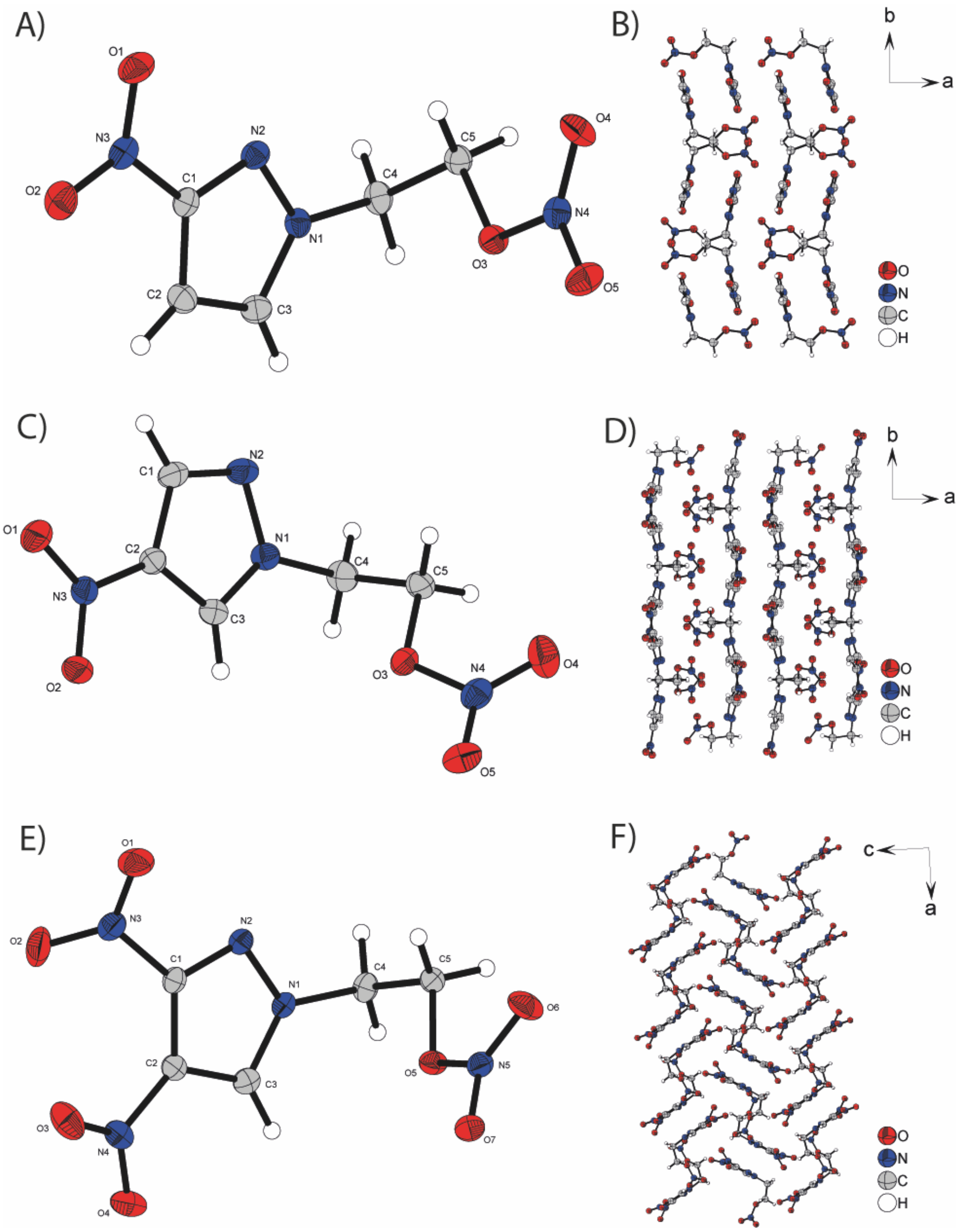

Figure S2: Crystal structures of 1-nitratoethyl-nitropyrazole compounds: **A**, **B**) 1-nitratoethyl-3-nitropyrazole (**5**); **C**, **D**) 1-nitratoethyl-4-nitropyrazole (**9**); **E**, **F**) 1-nitratoethyl-3,4-dinitropyrazole (**6**).

Compound **11**, **13** and **19** crystallized directly from the reaction mixture or ethanol, forming colourless platelets (**11**, **19**) or blocks (**13**). Compounds **11** and **19** precipitated in a common space groups (**11**: *Pna*2$_1$; **19**: *P*2$_1$) and have four molecules per unit cell, with a cell volume of 724.63(15) Å^3 and 890.1(1) Å^3, respectively. At 173 K, the calculated densities are 1.728 g cm^{-3} and 1.901 g cm^{-3}. At a temperature of 123 K, compound **13** formed a crystal lattice in the *C*2/*c* space group, consisting of eight molecules in the unit cell and having a volume of 1406.3(3) Å^3. The calculated density at this temperature is 1.588 g cm^{-3}. Molecular units and extended structures are illustrated in **Figure S3**.

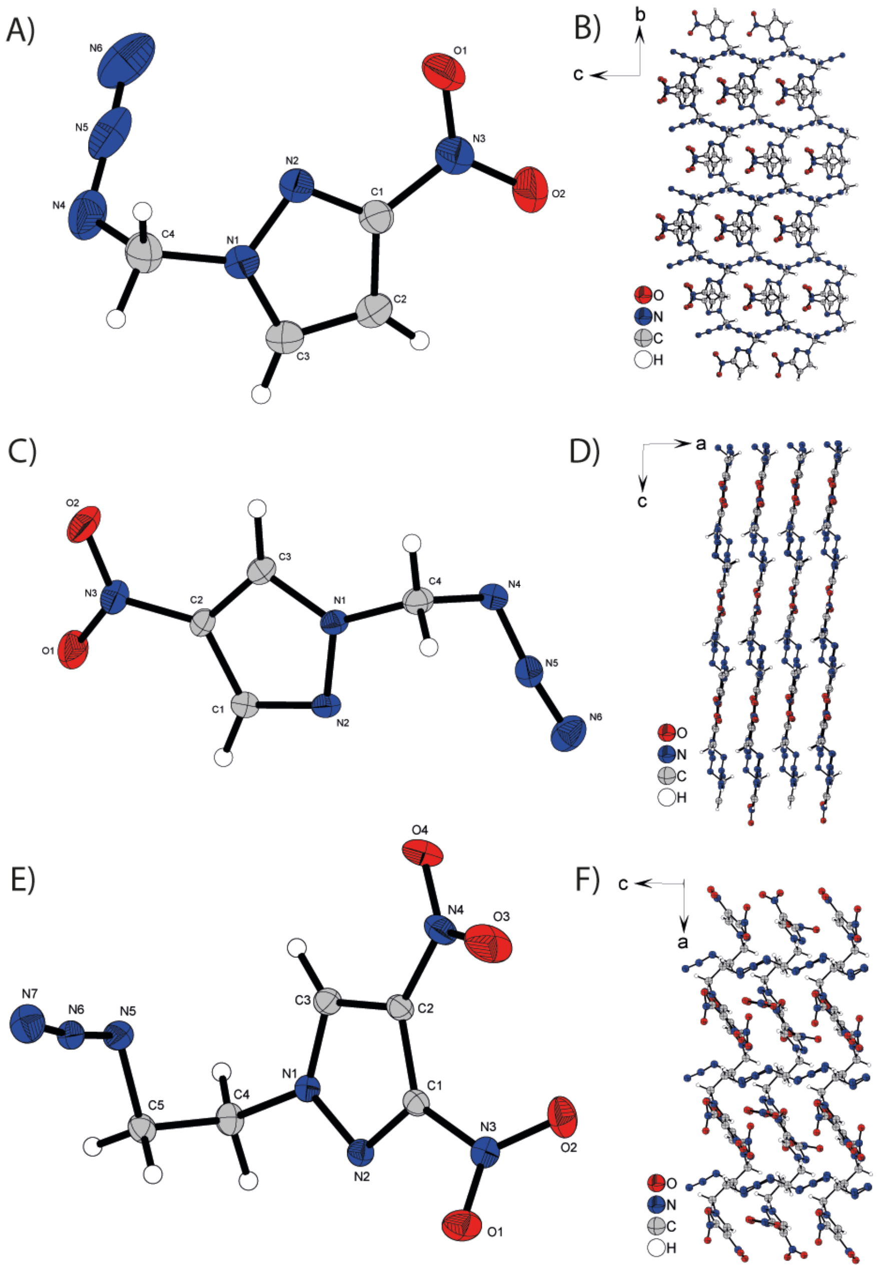

Figure S3: Crystal structures of 1-azidoalkyl-nitropyrazole compounds: **A**, **B**) 1-azidomethyl-3-nitropyrazole (**11**); **C**, **D**) 1-azidomethyl-4-nitropyrazole (**13**); **E**, **F**) 1-azidoethyl-3,4-dinitropyrazole (**19**).

Compound **16** and **20** can be obtained as yellow plates or colorless blocks by crystallization from EtOAc or Et2O. They crystallize in common space groups (**16**: *Pbca*; **20**: $P2_1/c$) and have eight and four molecules per unit cell, with respective cell volumes of 1633.0(2) Å^3 and 936.45(8) Å^3. The densities calculated at 173 K are 1.702 g cm^{-3} and 1.583 g cm^{-3}. Molecular units and extended structures are illustrated in **Figure S4**.

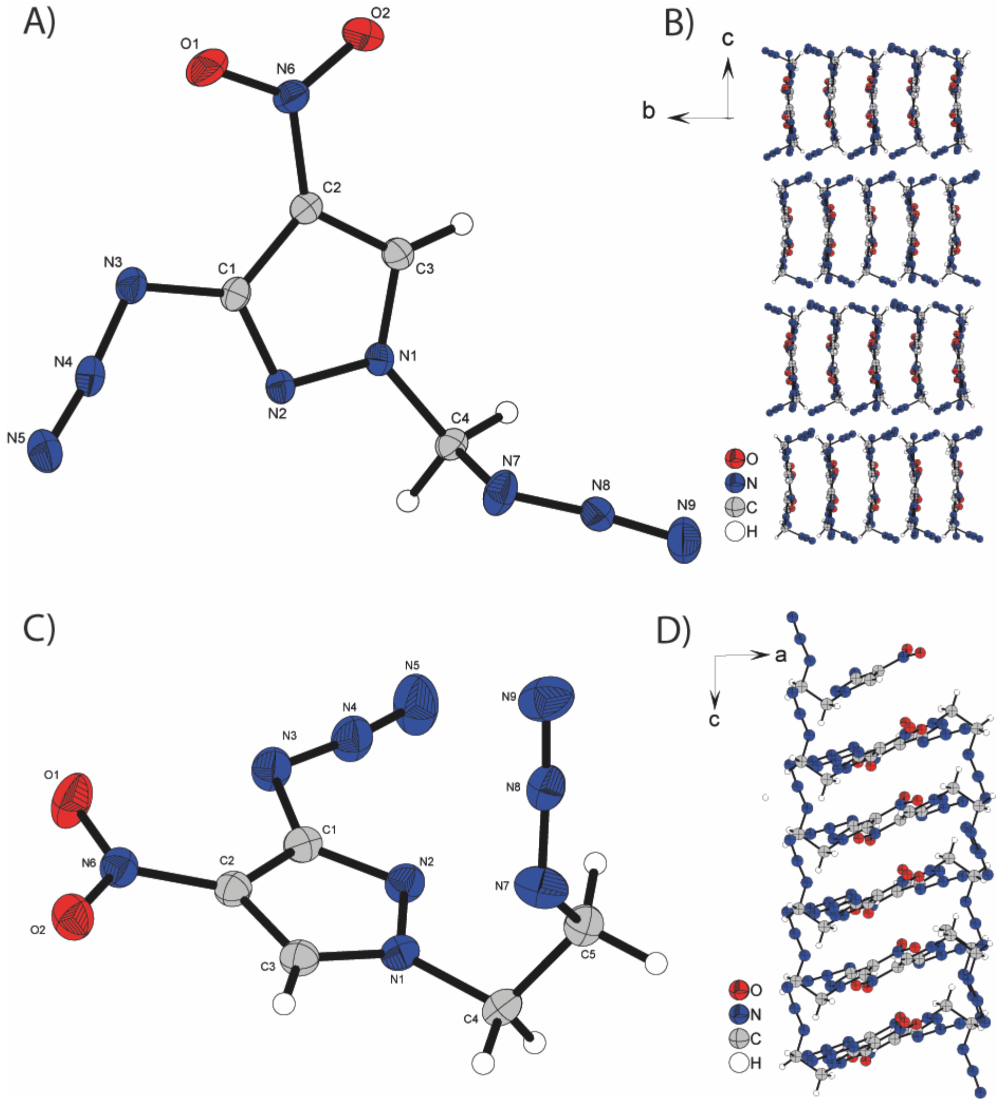

Figure S4: Crystal structures of 1-azidoalkyl-3-azido-4-nitropyrazole compounds: **A**, **B**) 1-azidomethyl-3-azido-4-nitropyrazole (**16**); **C**, **D**) 1-azidoethyl-3-azido-4-nitropyrazole (**20**).

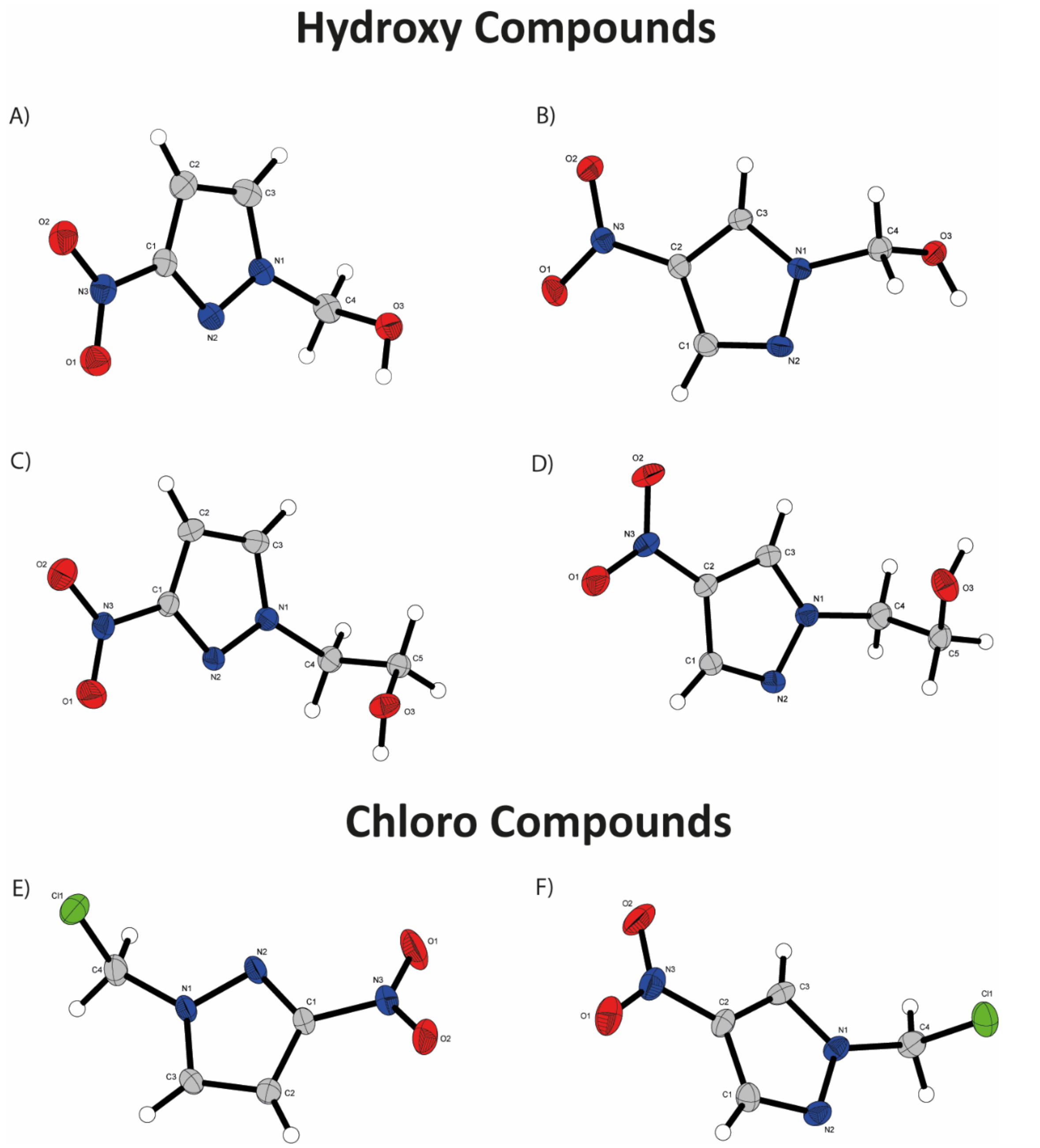

Figure S5: Crystal structures of precursor compounds: **A**) 1-hydroxymethyl-3-nitropyrazole (**1**); **B**) 1-hydroxymethyl-4-nitropyrazole (**7**); **C**) 1-hydroxyethyl-3-nitropyrazole (**4**); **D**) 1-hydroxyethyl-4-nitropyrazole (**8**); **E**) 1-chloromethyl-3-nitropyrazole (**10**); **F**) 1-chloromethyl-4-nitropyrazole (**12**).

3. Heat of formation calculation

All quantum chemical calculations were performed using the Gaussian G09 program package.[S6] The complete basis set (CDS) method of Petersson and coworkers was used for calculation of enthalpies (H) and free energies (G), listed in Table S6 in order to obtain very accurate energies. The CBS models use the known asymptotic convergence of pair natural orbital expressions to extrapolate from calculations using a finite basis set to the estimated complete basis set limit. CBS-4 starts with a HF/3-21G(d) geometry optimization; the zero-point energy is computed at the same level. It then uses a large basis set SCF calculation as a base energy, and a MP2/6-31+G calculation with a CBS extrapolation to correct the energy through second order. A MP4(SDQ)/6-31+(d,p) calculation is used to approximate higher order contributions. In this study, we applied the modified CBS-4M method (M referring to the use of minimal population localization), which is a re-parametrized version of the original CBS-4 method and also includes some additional empirical corrections.[S7] The gas-phase enthalpies ($\Delta_f H°$(g, M, 298)) of the species were computed according to the atomization energy method (**Equation S1**) using room temperatures CBS-4M enthalpies (**Table S7**).[S8]

$$\Delta_f H°_{(g,\ M,\ 298)} = H_{(molecule,\ 298)} - \sum H°_{(atoms,\ 298)} + \sum \Delta_f H°_{(atoms,\ 298)} \quad \textbf{(Eq. S1)}$$

Table S7: CBS-4M electronic enthalpies for atoms C, H, N and O and their literature values.

	$-H^{298}$ / a.u.	$\Delta_f H°_{gas}$ [S9]
H	0.500991	217.998
C	37.786156	716.68
N	54.522462	472.68
O	74.991202	249.18

In order to obtain the energy of formation for the solid phase of all compounds, heat of formations of the condensed phase were calculated by subtracting the heats of sublimation or vaporization, respectively, obtained from RoseBoom 2.3.[S10]

Table S8: CBS-4M results and calculated gas-phase enthalpies.

	$-H^{298}$ [a] [a.u.]	$\Delta_f H°$(g, M) [b] [kJ mol^{-1}]	$\Delta_{sub}H$ /$\Delta_{vap}H$ [c] [kJ mol^{-1}]	$\Delta_f H°$(s)[d] [kJ mol^{-1}]
**2**	−748.781256	83.7	103.9	−20.2
**3**	−953.057307	122.1	113.4	8.7
**5**	−788.025077	39.9	107.0	−67.1
**6**	−992.302632	74.4	117.5	−43.1
**9**	−788.024279	42.0	107.0	−65.0
**11**	−632.770512	496.9	95.1	401.8
**13**	−632.77169	493.8	95.1	398.7
**15**	−837.045917	537.1	103.2	433.9
**17**	−672.01395	454.2	84.6	369.6
**18**	−672.013012	456.7	84.6	372.1
**19**	−876.291745	488.1	105.6	382.5
**16**	−796.155912	859.4	103.6	755.8
**20**	−835.400843	812.8	105.4	707.4

[a] CBS-4M electronic enthalpy; [b] gas phase enthalpy of formation. [c] Calculated with RoseBoom 2.3 [d] Calculated heat of formation for the condensed phase

4. Physico-chemical properties

Table S9: Physicochemical properties and detonation parameter of nitric ester (**2**, **3**, **5**, **6**, **9**) compared to **TNT**.

	**(2)**	**(3)**	**(5)**	**(6)**	**(9)**	**TNT**
Formula	$C_4H_4N_4O_5$	$C_5H_3N_5O_7$	$C_5H_6N_4O_5$	$C_5H_5N_5O_7$	$C_5H_6N_4O_5$	$\mathbf{C_7H_5N_3O_6}$
FW [g·mol⁻¹]	188.10	233.10	202.14	247.12	202.14	**227.13**
IS[a] [J]	>40	4	>40	30	>40	**15**
FS[b] [N]	>360	288	>360	>360	>360	**>360**
Ω_{CO2}[d] [%]	−43	−17	−63	−36	−63	**−74.0**
T_{endo}[e] [°C]	70	93	78	61	52	**81**
$T_{exo.}$[f] [°C]	161	159	198	198	191	**289**
ρ[g] [g·cm⁻³]	1.696	1.848	1.629	1.734	1.613	**1.65**
$\Delta_f H°$[h] [kJ·mol⁻¹]	−20.2	8.7	−67.1	−43.1	−65.0	**−185**
**EXPLO5 V6.05 values**						
$-\Delta_E U°$[i] [kJ·kg⁻¹]	4869	5693	4398	5152	4399	**5022**
T_{C-J} [j] [K]	3491	4059	3082	3645	3096	**3452**
p_{C-J} [k][GPa]	24.3	33.6	19.8	26.7	19.5	**20.5**
D_{C-J} [l] [m·s⁻¹]	7675	8668	7163	7932	7110	**6950**
V^0 [m] [dm³·kg⁻¹]	716	707	730	711	732	**633**

[a] Impact sensitivity (BAM drophammer, method 1 of 6); [b] friction sensitivity (BAM friction tester, method 1 of 6); [c] electrostatic discharge sensitivity (OZM XSpark10); [d] oxygen balance toward carbon dioxide (Ω_{CO2} = (*n*O − 2*x*C − *y*H/2)(1600/FW)); [e] endothermic event (DTA, β = 5 °C·min⁻¹); [f] temperature of decomposition (DTA, β = 5 °C·min⁻¹); [g] density at 298 K recalculated from X-ray data; [h] heat of formation (calculated using the atomization method and CBS-4M enthalpies; [i] detonation energy; [j] detonation temperature; [k] detonation velocity; [l] detonation pressure; [m] volume of detonation gases at standard temperature and pressure conditions; [n] determined at LMU.

Table S10: Physicochemical properties and detonation parameter of azides (**11**, **13**, **15**, **17**, **18**, **19**) compared to **TNT**.

	**(11)**	**(13)**	**(15)**	**(17)**	**(18)**	**(19)**	**TNT**
Formula	$C_4H_4N_6O_2$	$C_4H_4N_6O_2$	$C_4H_3N_7O_4$	$C_5H_6N_6O_2$	$C_5H_6N_6O_2$	$C_5H_5N_7O_4$	$\mathbf{C_7H_5N_3O_6}$
FW [g·mol⁻¹]	168.12	168.12	213.11	182.14	182.14	227.14	**227.13**
IS[a] [J]	>40	>40	>40	>40	>40	25	**15**
FS[b] [N]	>360	>360	>360	>360	>360	>360	**>360**
Ω_{CO2}[d] [%]	−76	−76	−41	−97	−97	−60	**−74.0**
T_{endo}[e] [°C]	40	42	/	/	/	50	**81**
$T_{exo.}$[f] [°C]	179	174	154	214	214	216	**289**
ρ[g] [g·cm⁻³]	1.513	1.547	1.58[o]	1.28[o]	1.34[o]	1.664	**1.65**
$\Delta_f H°$[h] [kJ·mol⁻¹]	401.8	398.7	433.9	369.6	372.1	382.5	**−185**
**EXPLO5 V6.05 values**							
$-\Delta_E U°$[i] [kJ·kg⁻¹]	4483	4497	5241	4167	4222	4900	**5022**
T_{C-J} [j] [K]	3380	3314	3998	2910	2914	3483	**3452**
p_{C-J} [k][GPa]	19.9	19.8	23.1	11.9	13.3	22.9	**20.5**
D_{C-J} [l] [m·s⁻¹]	6972	7100	7679	6235	6483	7639	**6950**
V^0 [m] [dm³·kg⁻¹]	742	740	768	771	761	738	**633**

[a] Impact sensitivity (BAM drophammer, method 1 of 6); [b] friction sensitivity (BAM friction tester, method 1 of 6); [c] electrostatic discharge sensitivity (OZM XSpark10); [d] oxygen balance toward carbon dioxide (Ω_{CO2} = (*n*O − 2*x*C − *y*H/2)(1600/FW)); [e] endothermic event (DTA, β = 5 °C·min⁻¹); [f] temperature of decomposition (DTA, β = 5 °C·min⁻¹); [g] density at 298 K recalculated from X-ray data; [h] heat of formation (calculated using the atomization method and CBS-4M enthalpies; [i] detonation energy; [j] detonation temperature; [k] detonation velocity; [l] detonation pressure; [m] volume of detonation gases at standard temperature and pressure conditions; [n] determined at LMU; [o] volumetric determination.

Table S11: Physicochemical properties and detonation parameter of azides (**16**, **20**) compared to **TNT**.

	**(16)**	**(20)**	**TNT**
Formula	$C_4H_3N_9O_2$	$C_5H_5N_9O_2$	**$C_7H_5N_3O_6$**
FW [g·mol^{-1}]	209.13	223.16	**227.13**
IS[a] [J]	<1	2	**15**
FS[b] [N]	10	80	**>360**
Ω_{CO2}[d] [%]	−57	−75	**−74.0**
T_{endo}[e] [°C]	57	41	**81**
$T_{exo.}$[f] [°C]	157	172	**289**
ρ[g] [g·cm^{-3}]	1.670	1.554	**1.65**
$\Delta_f H°$[h] [kJ·mol^{-1}]	755.8	707.4	**−185**
**EXPLO5 V6.05 values**			
$-\Delta_E U°$[i] [kJ·kg^{-1}]	5177	4709	**5022**
$T_{C\text{-}J}$ [j] [K]	3812	3416	**3452**
$p_{C\text{-}J}$ [k][GPa]	24.4	20.1	**20.5**
$D_{C\text{-}J}$ [l] [m·s^{-1}]	7945	7274	**6950**
V^0 [m] [dm^3·kg^{-1}]	748	751	**633**

[a] Impact sensitivity (BAM drophammer, method 1 of 6); [b] friction sensitivity (BAM friction tester, method 1 of 6); [c] electrostatic discharge sensitivity (OZM XSpark10); [d] oxygen balance toward carbon dioxide (Ω_{CO2} = (nO – $2x$C – yH/2)(1600/FW)); [e] endothermic event (DTA, β = 5 °C·min^{-1}); [f] temperature of decomposition (DTA, β = 5 °C·min^{-1}); [g] density at 298 K recalculated from X-ray data; [h] heat of formation (calculated using the atomization method and CBS-4M enthalpies; [i] detonation energy; [j] detonation temperature; [k] detonation velocity; [l] detonation pressure; [m] volume of detonation gases at standard temperature and pressure conditions; [n] determined at LMU.

5. ^{15}NMR spectroscopy

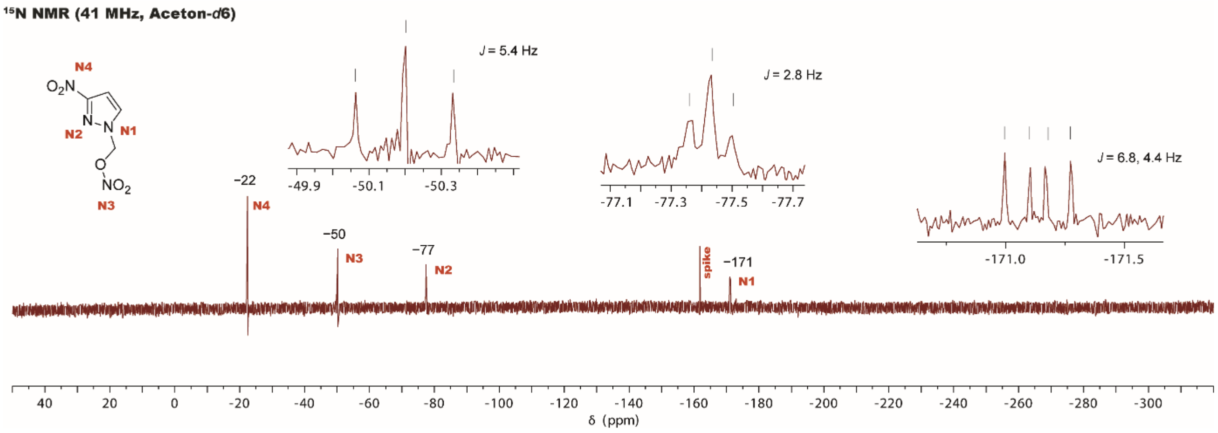

Figure S6: Proton coupled ^{15}N NMR spectrum of compound **2**.

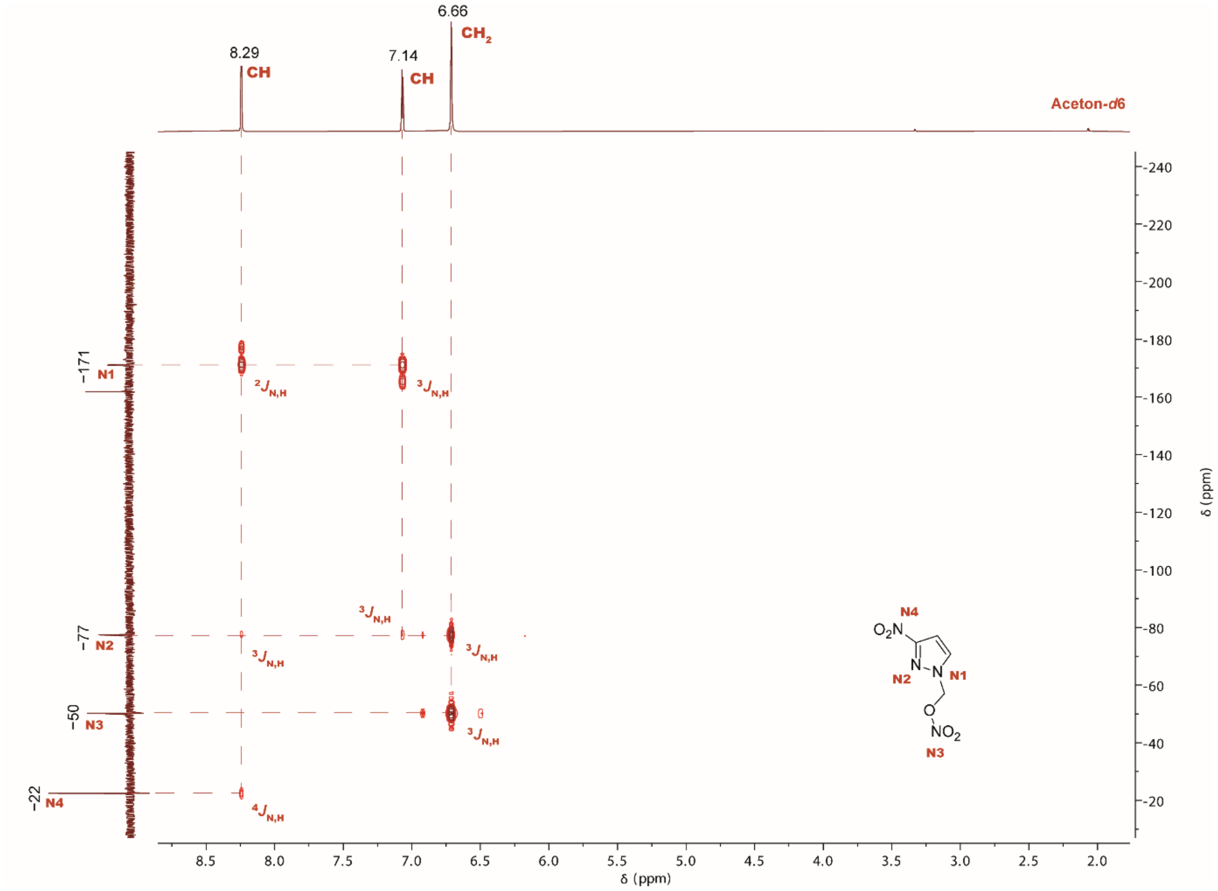

Figure S7: ^{1}H-^{15}N HMBC NMR spectrum of compound **2**.

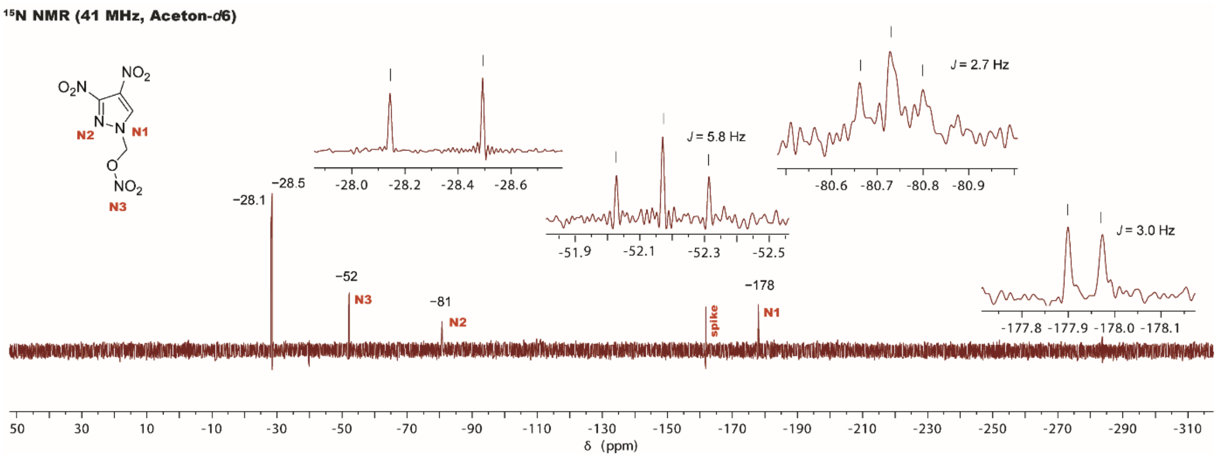

Figure S8: Proton coupled ^{15}N NMR spectrum of compound **3**.

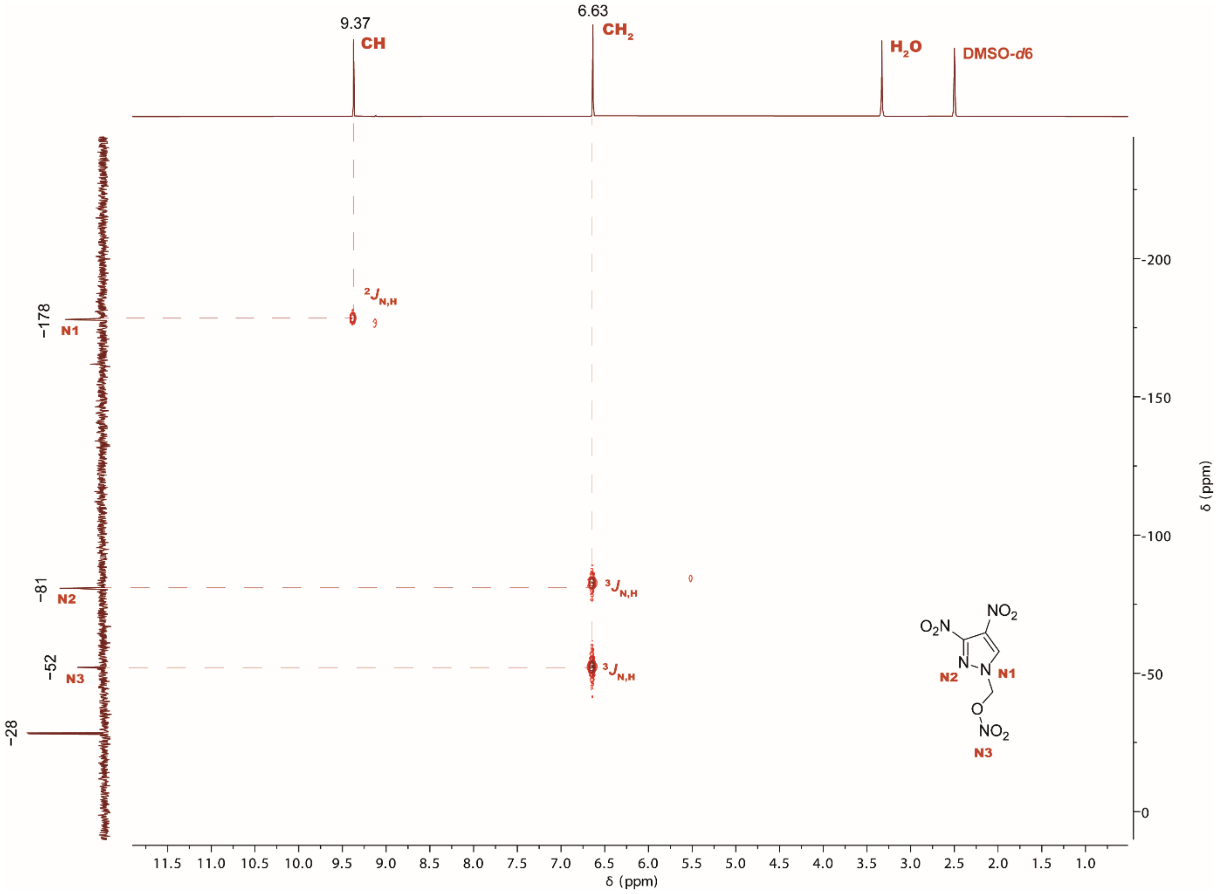

Figure S9: ^{1}H-^{15}N HMBC NMR spectrum of compound **3**.

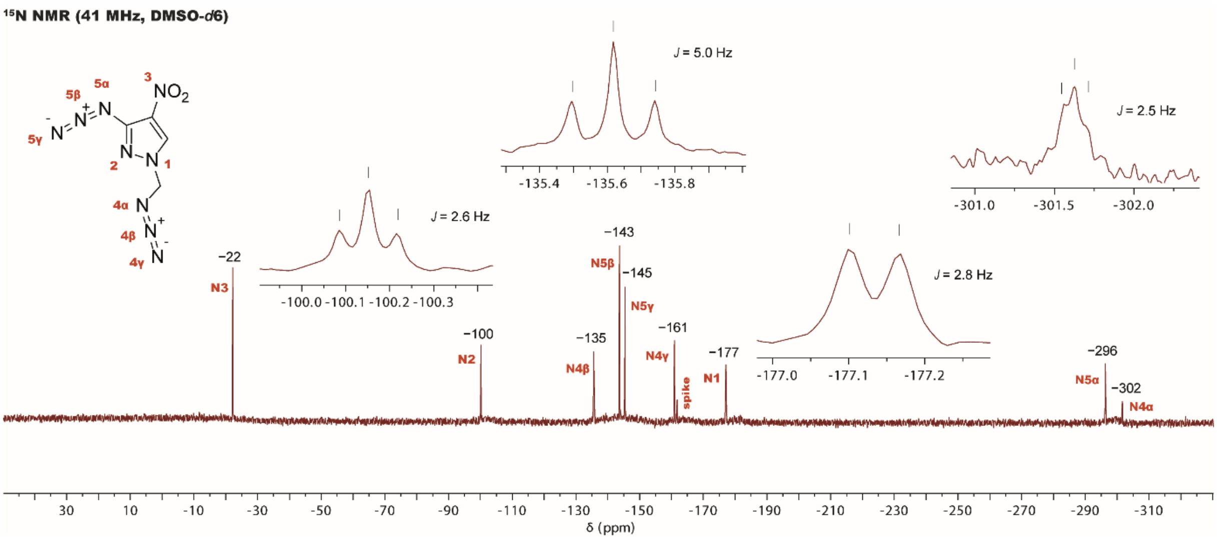

Figure S10: Proton coupled ^{15}N NMR spectrum of compound **16**.

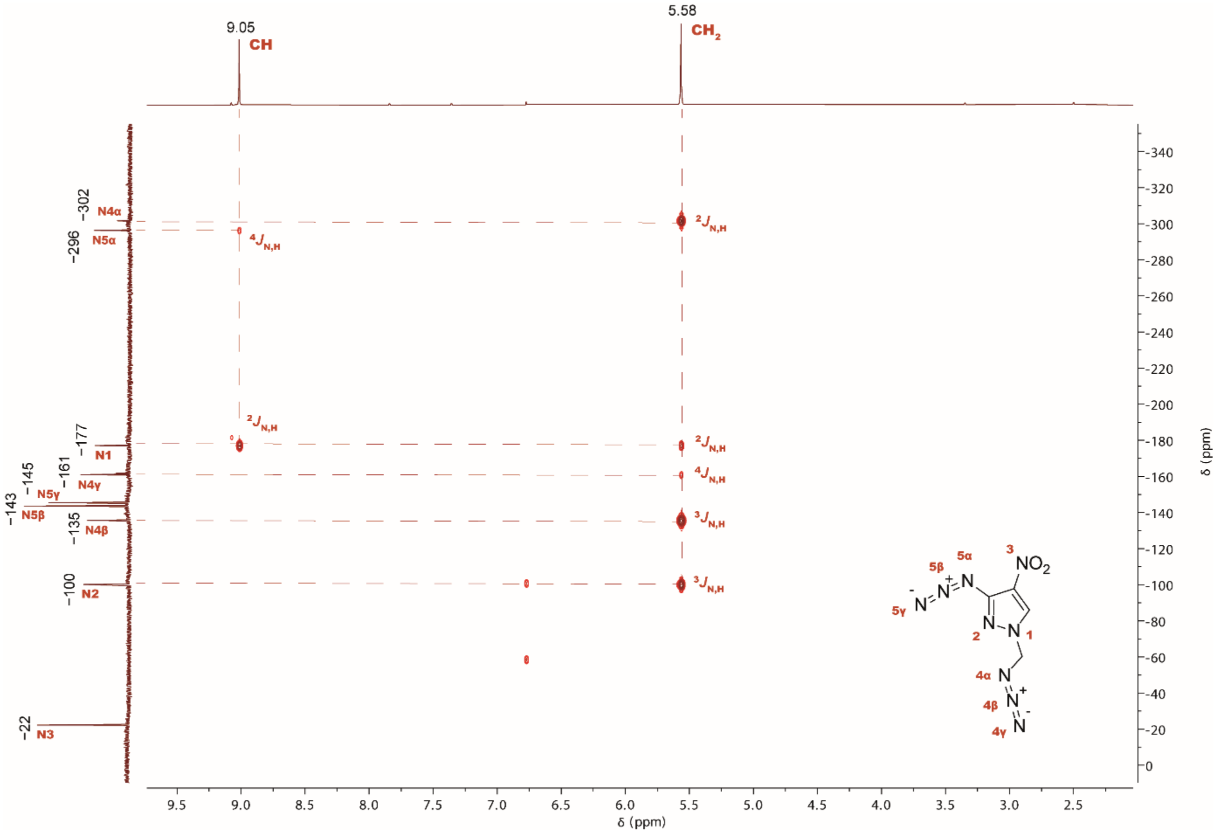

Figure S11: ^{1}H-^{15}N HMBC NMR spectrum of compound **16**.

6. Thermal stability

Differential thermal analysis (DTA) was measured on an OZM Research DTA 552-Ex device in a range of 25−400 °C at a heating rate of 5 °C min^{-1}. Thermogravimetric measurements (TGA) were performed with a Perkin-Elmer TGA 4000 apparatus using a heating rate of 5 °C min^{-1} in a slow stream of nitrogen gas (1mL/min).

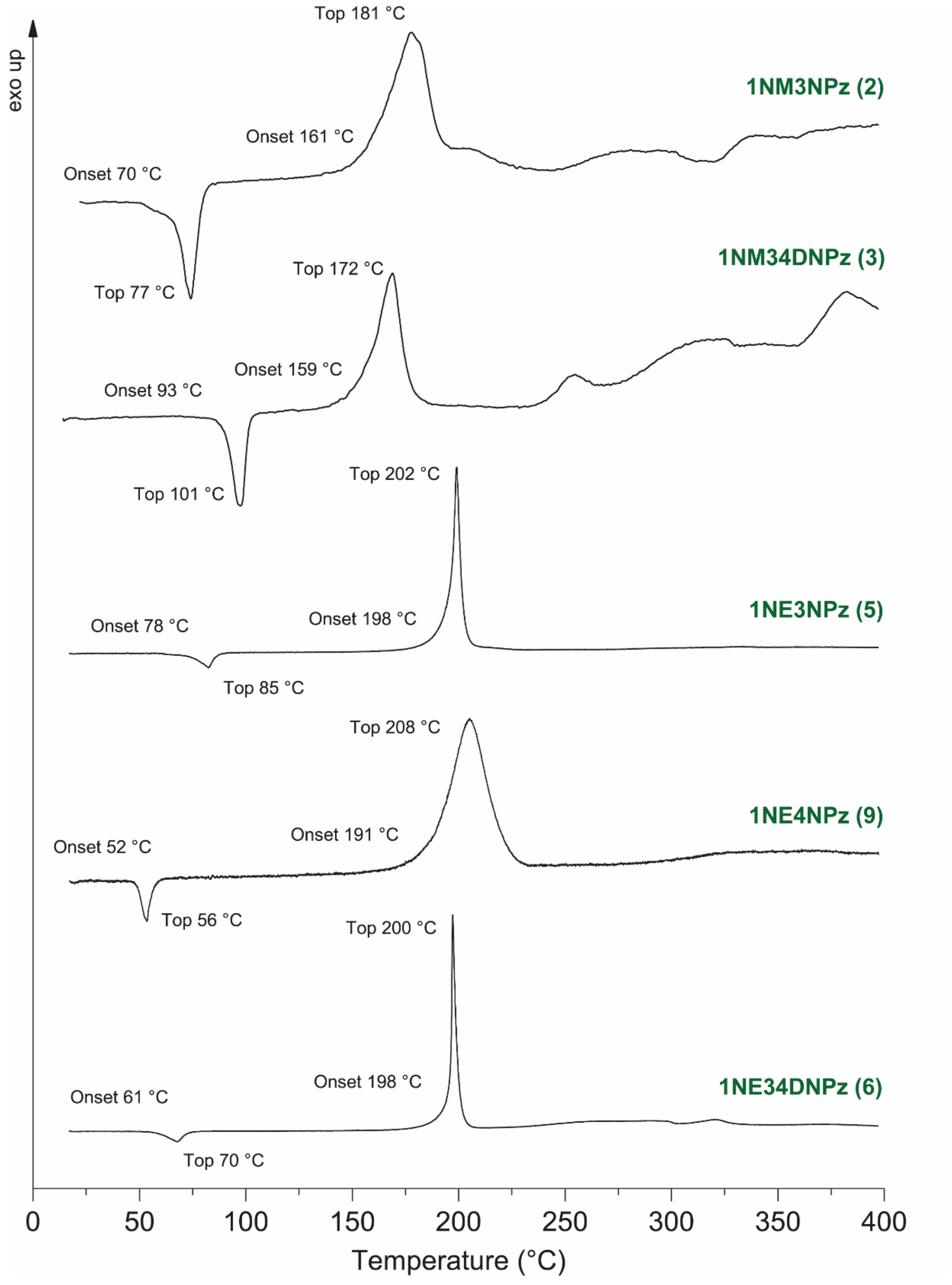

Figure S12: DTA spectra of nitratoalkyl-compounds **2**, **3**, **5**, **6** and **9**.

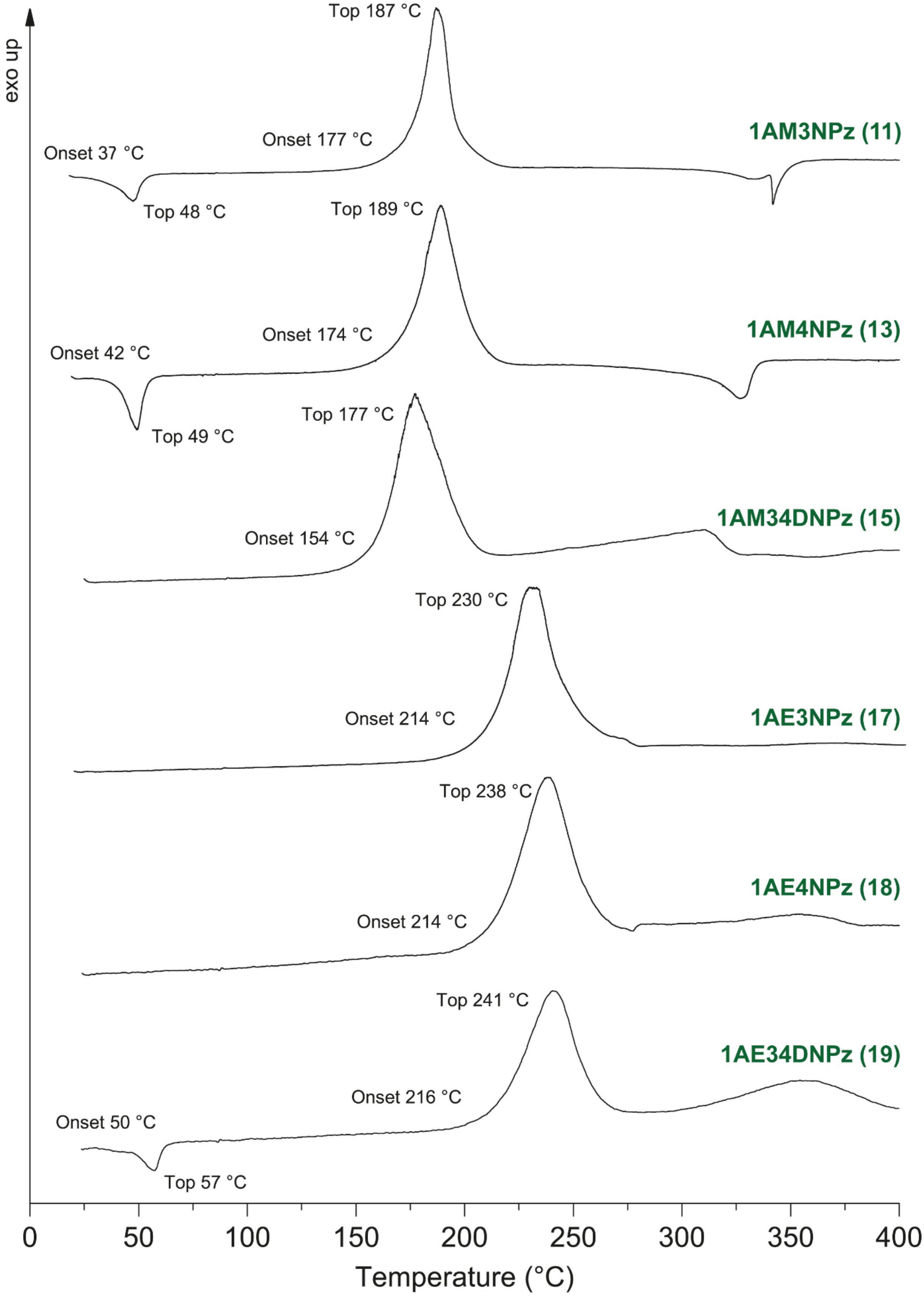

Figure S13: DTA spectra of azidoalky-compounds **11**, **13**, **15**, **17**, **18** and **19**.

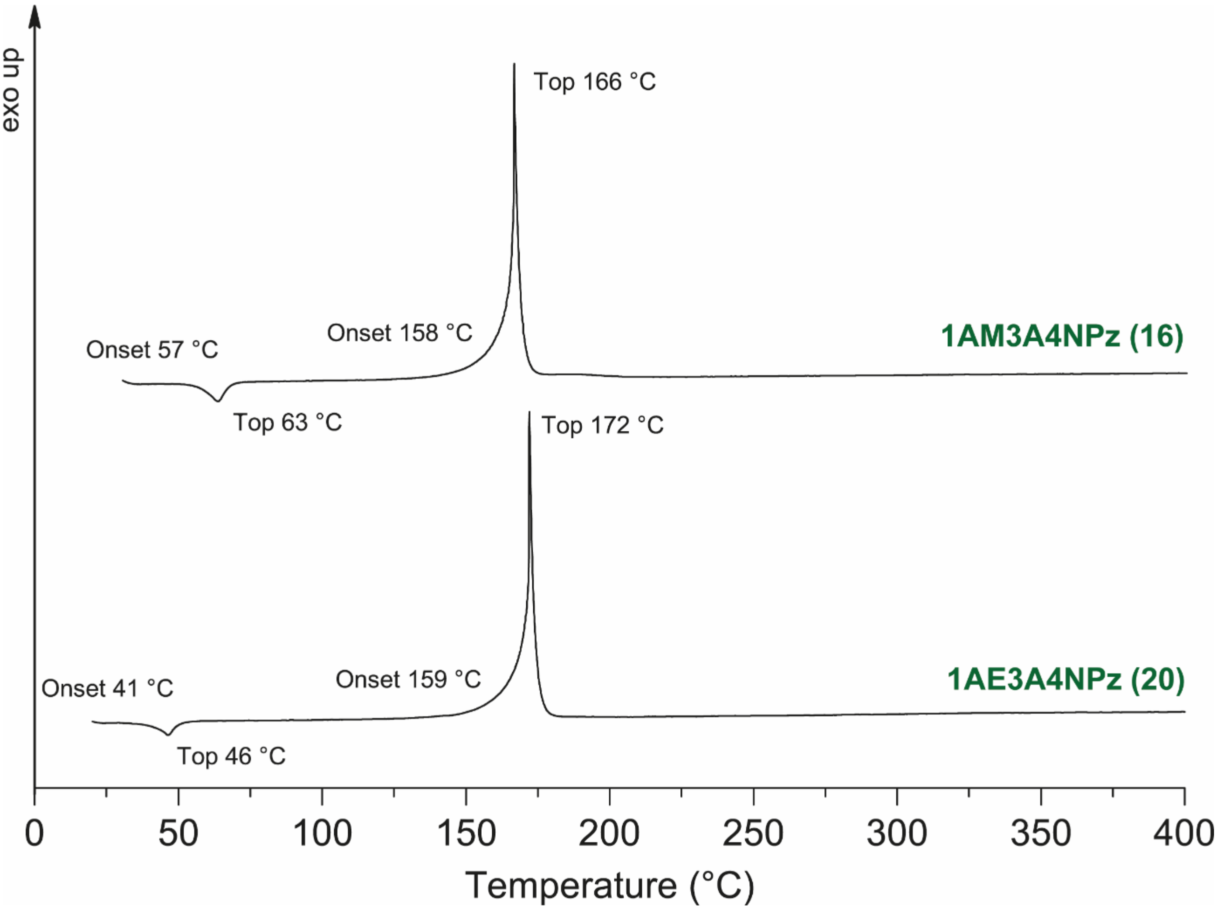

Figure S14: DTA spectra of azidoalky-azido-nitro-compounds **16** and **20**.

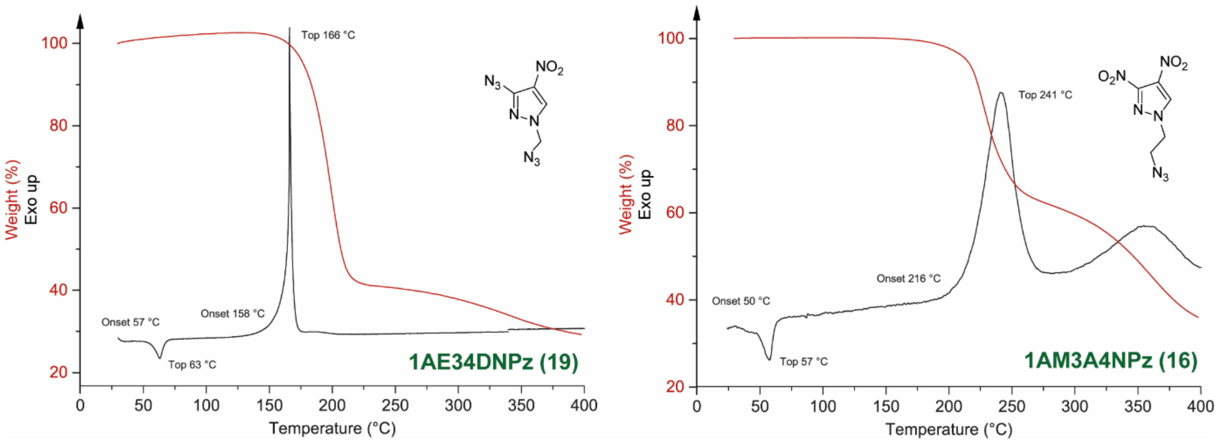

Figure S15: Combined DTA-TGA spectrum of azidoalkyl-compounds **16** and **19**.

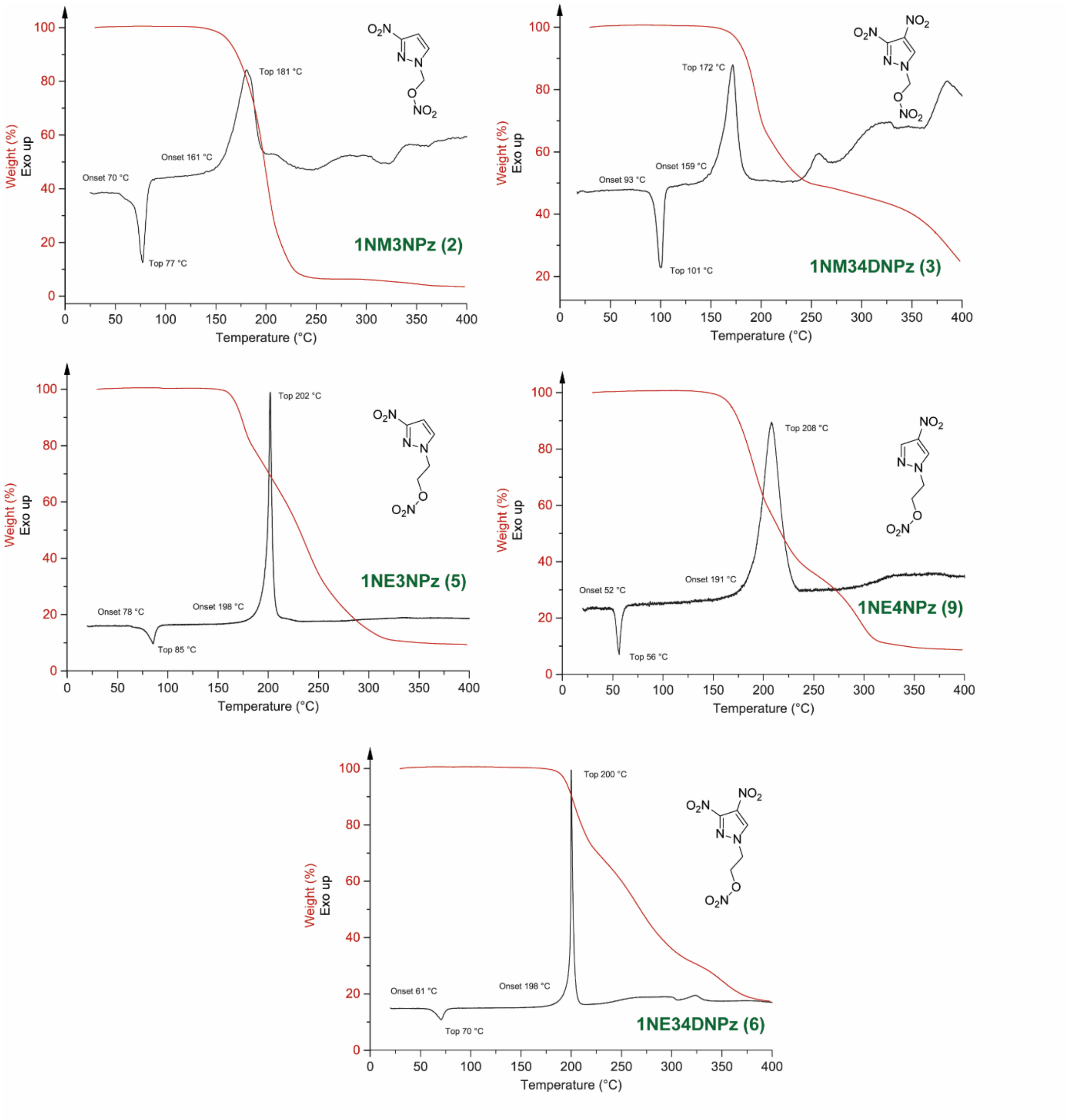

Figure S16: Combined DTA-TGA spectrum of nitratoalkyl-compounds **2**, **3**, **5**, **6** and **9**.

7. Compatibilities

To ensure the safe handling of energetic substances, it is necessary to consider their thermal stability when they come into contact with other materials. This is particularly important for applications such as explosives and pyrotechnics. In order to assess the compatibility of these substances with various additives, different thermal methods such as differential thermal analysis (DTA), thermogravimetric analysis (TGA), pressure increase in closed systems (PIST), and vacuum stability test (VST) can be utilized. In this study, the DTA method was specifically employed to investigate the compatibility of the energetic substances with different additives (RDX and HMX). Differential thermal analysis (DTA) was measured on an OZM Research DTA 552-Ex device in a range of 25−400 °C at a heating rate of 5 °C min^{-1}. The substances were mixed in the ratio of 1:1.

Compatibilities were evaluated according to the standardized procedure outlined in STANAG 4147.[S11] The analysis involved determining the difference between the endothermic and exothermic maxima of the pure substance and its mixture with the test component. The criteria for determining compatibilities are presented in **Table S12**.

Table S12: Criteria for compatibilities.

temperature difference	description
$\Delta T \leq 4$ °C	compatible
4 °C < $\Delta T \leq 20$ °C	moderately
$\Delta T > 20$ °C	incompatible

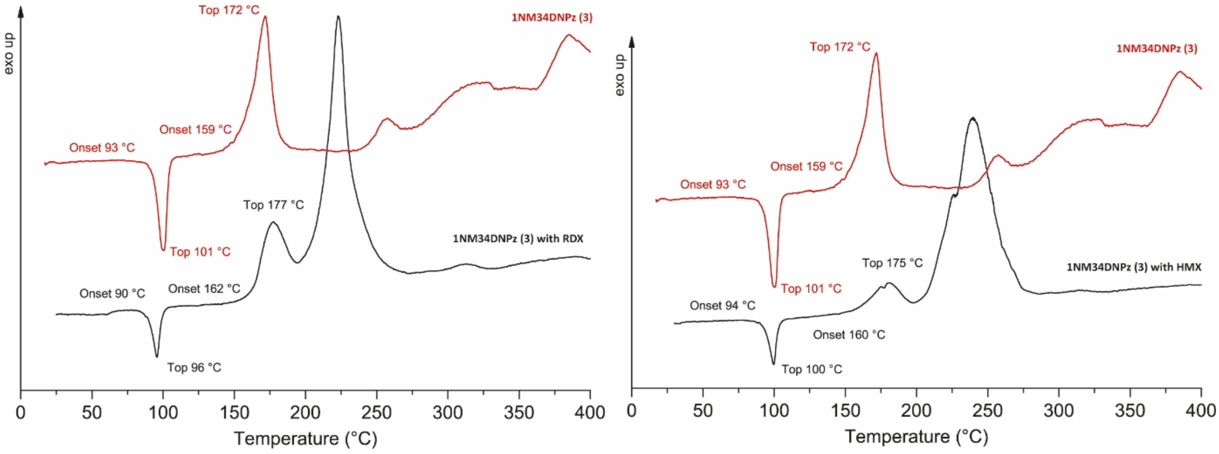

Figure S17: DTA spectra of compatibility measurements of compound **3** with **RDX** (left) and **HMX** (right).

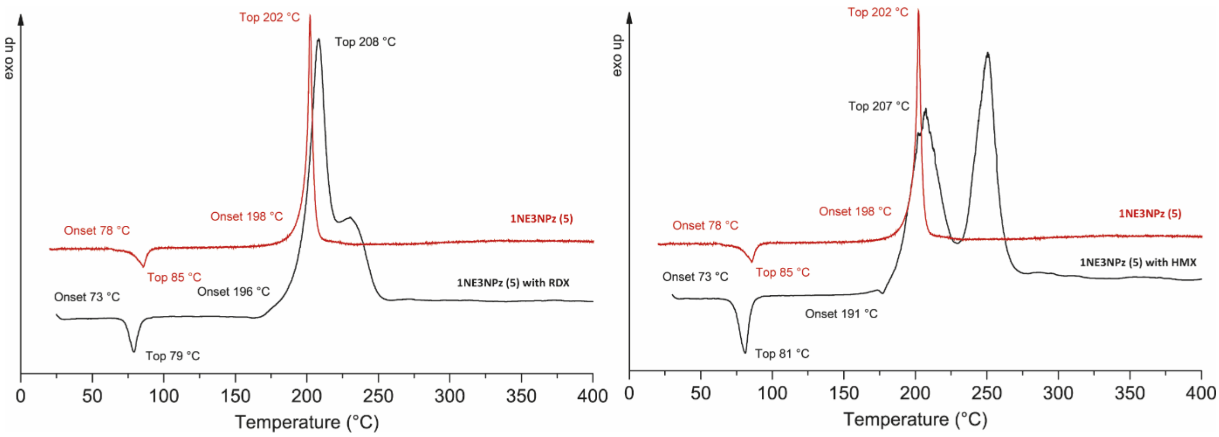

Figure S18: DTA spectra of compatibility measurements of compound **5** with **RDX** (left) and **HMX** (right).

Table S13: Compatibilities of compounds **3** and **5** with **RDX** and **HMX**.

**compound**	**melting [°C]**		**decompositon [°C]**	
	**on**	**top**	**on**	**top**
**1-nitratomethyl-3,4-dinitropyrazole**	**93**	**101**	**159**	**172**
1-nitratomethyl-3,4-dinitropyrazole (RDX)	90	96	162	177
**difference (RDX)**	**3**	**5**	**3**	**5**
1-nitratomethyl-3,4-dinitropyrazole (HMX)	94	100	160	175
**difference (HMX)**	**4**	**1**	**1**	**3**
**1-nitratoethyl-3-nitropyrazole**	**78**	**85**	**198**	**202**
1-nitratoethyl-3-nitropyrazole (RDX)	73	79	196	208
**difference (RDX)**	**5**	**6**	**2**	**6**
1-nitratoethyl-3-nitropyrazole (HMX)	73	81	191	207
**difference (HMX)**	**5**	**4**	**7**	**5**

8. LC-MS measurements

LC-MS (liquid chromatography – mass spectrometry) analysis was performed on a Shimadzu Prominence HPLC system using a Restek Ultra PFPP column (3µm, 150 x 4.6 mm) and a MSQ Plus mass spectrometer from Thermo Scientific in ESI mode. Eluting buffers were buffer A (0.01% formic acid in H2O) and buffer B (0.01% formic acid in MeCN) with the flow rate set to 0.65 mL/min. The gradient was 0 → 7 min, 5% → 80% buffer B. The elution was monitored at 220 nm.

For the experimental procedure, 0.5 g of 1-hydroxymethyl-3-nitropyrazole was introduced into a reaction vial containing 4 mL of fuming HNO_3 maintained at the desired temperature. Samples (100 µL) were taken at specific time intervals: 0 min, 10 min, 30 min, 1h, 2h, 3h, 4h, 5h, and 6h. Each sample was immediately quenched with water (500 µL) and subjected to extraction with EtOAc (300 µL). A 100 µL aliquot of the organic phase was further diluted with an additional 150 µL of EtOAc and injected into the LC-MS system for analysis.

To enhance compound identification during the experiment, separate measurements of the reactant, product, and potential by-products were performed using LC-MS. This allowed for the determination of specific retention times (**Table S14**) associated with each compound, enabling accurate assignment and analysis.

Table S14: Retention time and observed mass for different nitropyrazoles using LC-MS.

compound	retention time [min]	m/z
3-nitropyrazole	4.58	112
3,4-dinitropyrazole	6.32	157
1-hydroxymethyl-3-nitropyrazole	4.45	112
1-nitratomethyl-3-nitropyrazole	6.79	112
1-nitratomethyl-3,4-dinitropyrazole	7.73	157

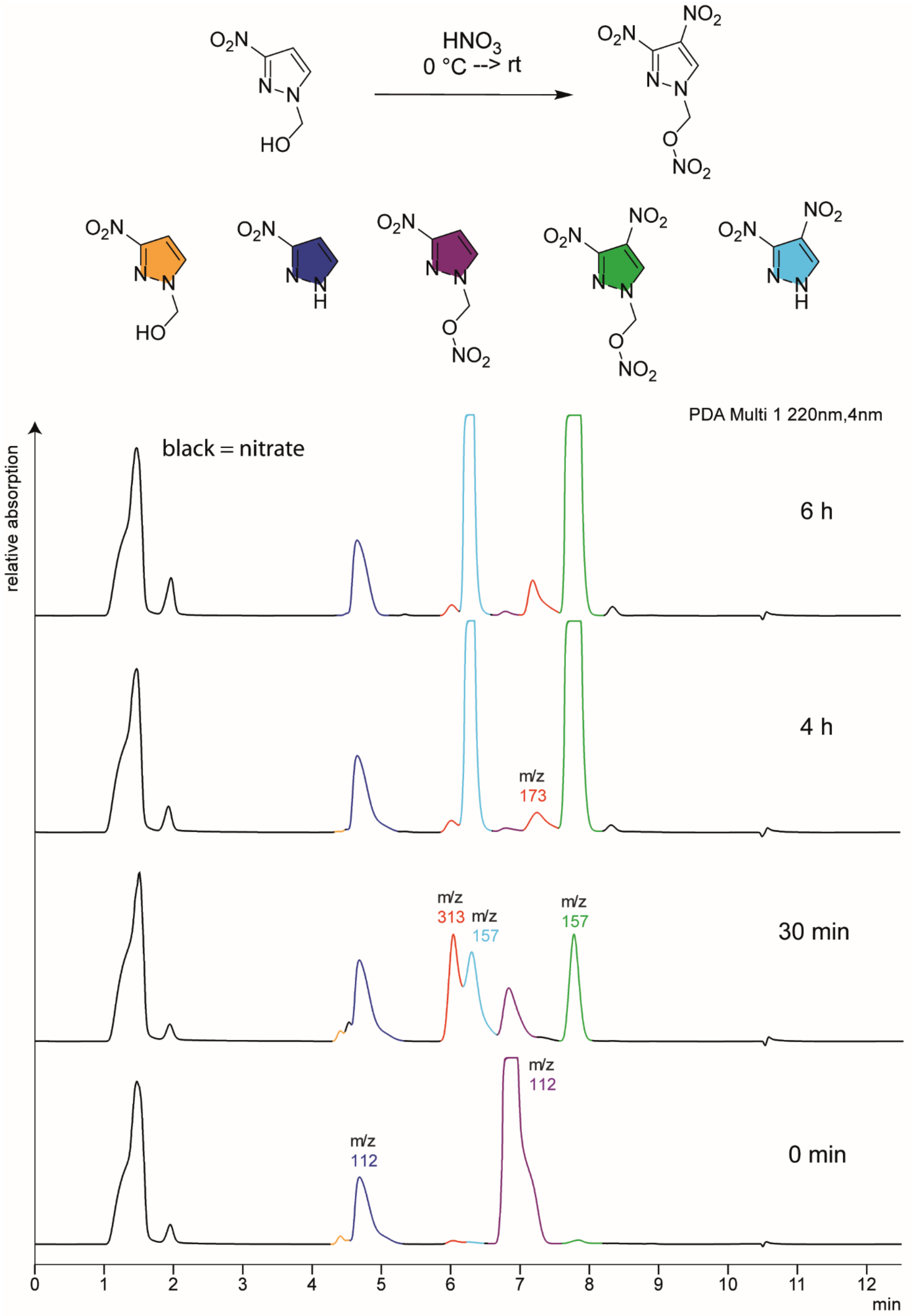

Figure S19: LC-MS monitoring of the nitration of 1-hydroxymethyl-3-nitropyrazole with fuming HNO_3 at 0 °C.

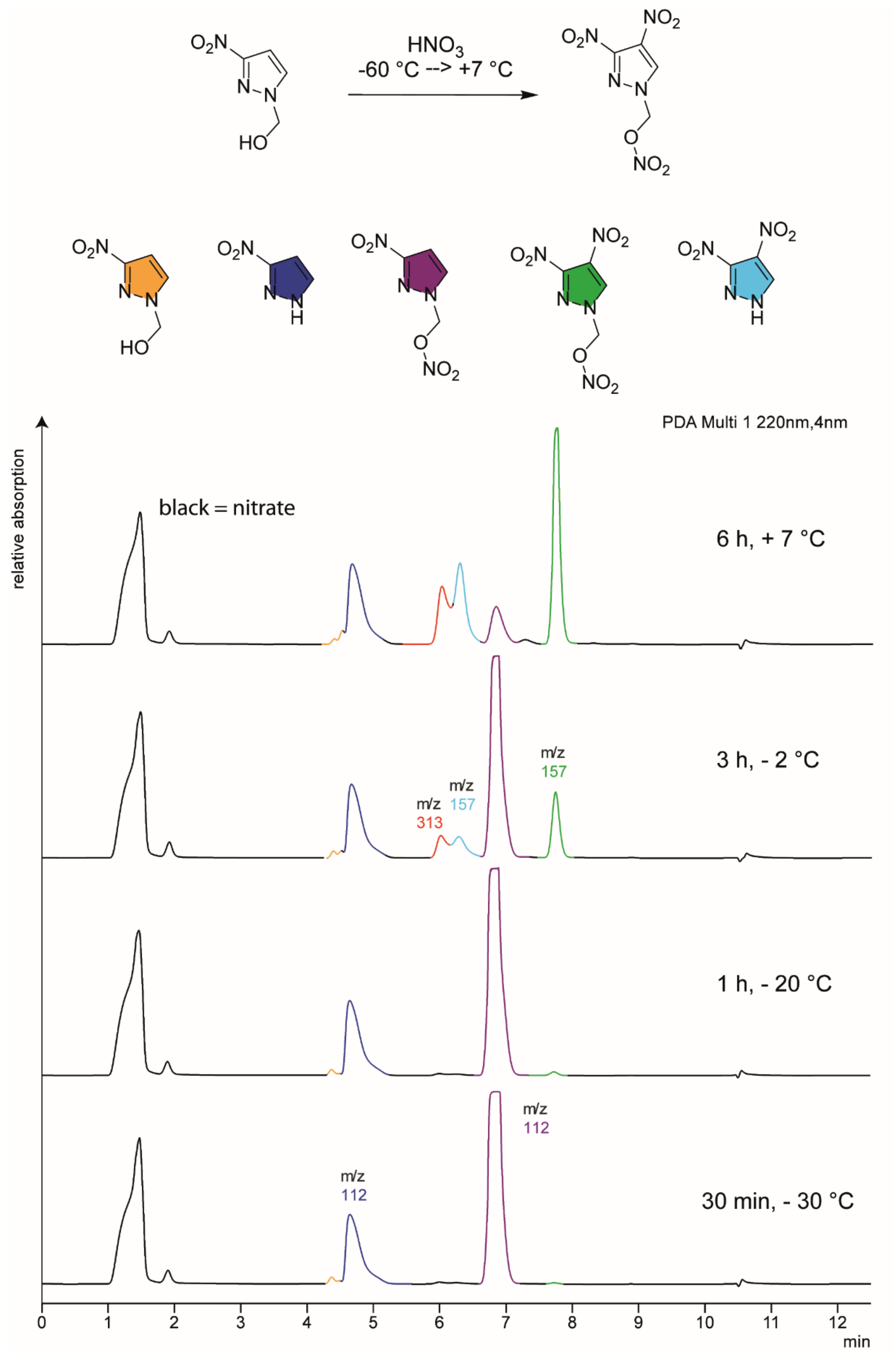

Figure S20: LC-MS monitoring of the nitration of 1-hydroxymethyl-3-nitropyrazole with fuming HNO_3 at −60 °C.

9. SSRT (small-scale shock reactivity test)

To assess the shock reactivity (explosiveness) of the investigated explosives, a small-scale shock reactivity test (SSRT) was conducted. The SSRT is designed to measure the shock reactivity of potentially energetic materials, even below critical diameter, without transitioning to detonation. The test setup combines the advantages of a lead block test[S12] and a gap test.[S13] Each compound was compacted into a perforated steel block. No attenuator (between detonator and sample) or air gap (between sample and aluminum block) was used. Initiation of the tested explosive was achieved using a commercially available detonator (Orica-DYNADET-C2-0ms). Dent sizes resulting from the shock were measured non-contactly using the XYZ-axis motorized 3D profilometer VR-5200, manufactured by Keyence (Osaka, Japan).
More information on the test and its implementation is described by Bauer et al.[S14]

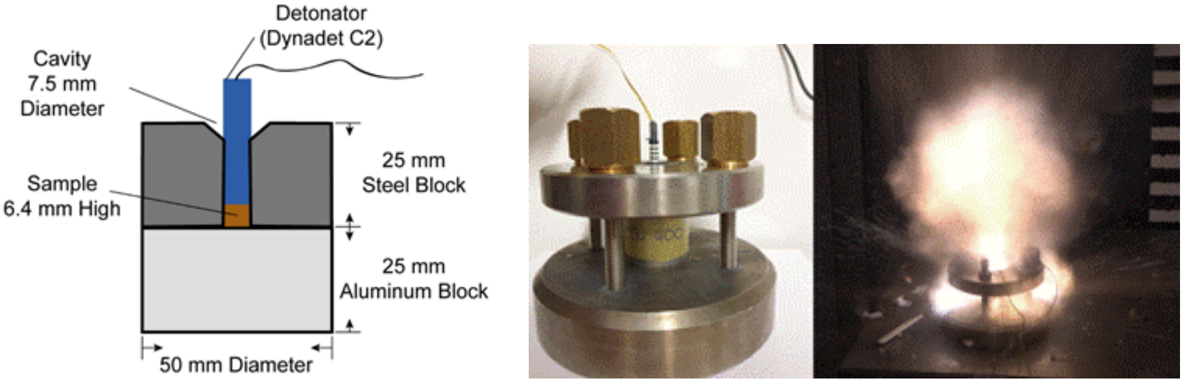

Figure S21: Setup of the SSRT experiment.[S14]

Evaluation of 1-nitratomethyl-3,4-dinitropyrazole (3)

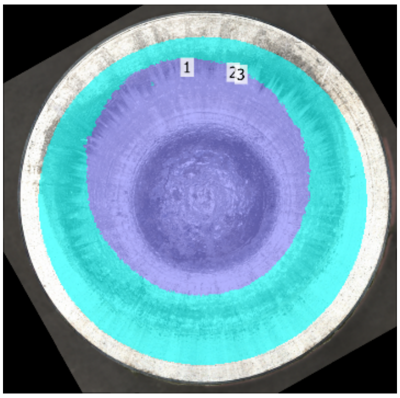

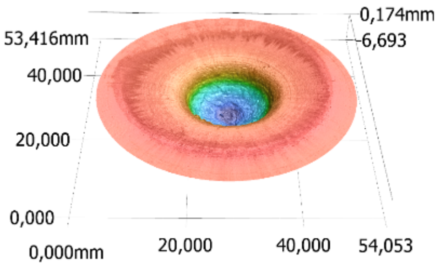

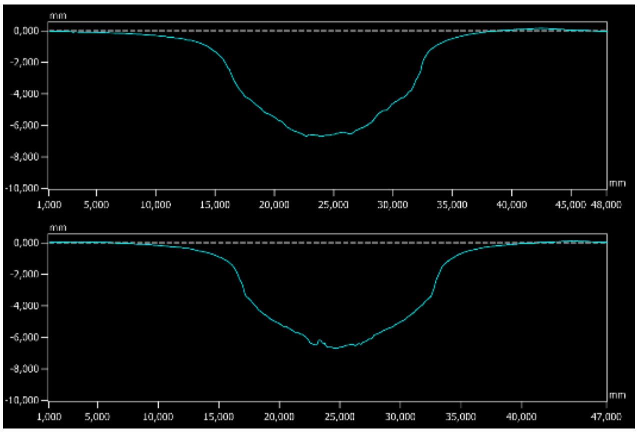

Table S15: SSRT results of 3 (Blast 1).

**1-Nitratomethyl-3,4-dinitropyrazole Blast 1**	
Measurement 1 [mm³]	1256,76
Measurement 2 [mm³]	1257,21
**Average Volume [mm³]**	**1256,98**

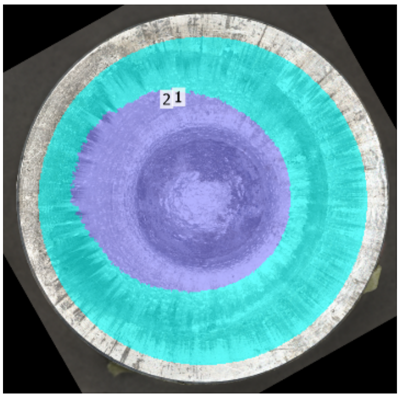

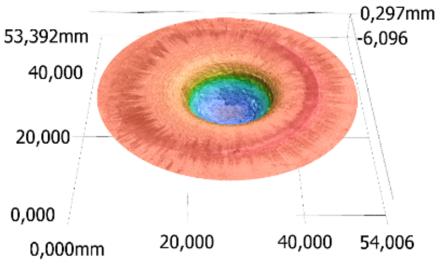

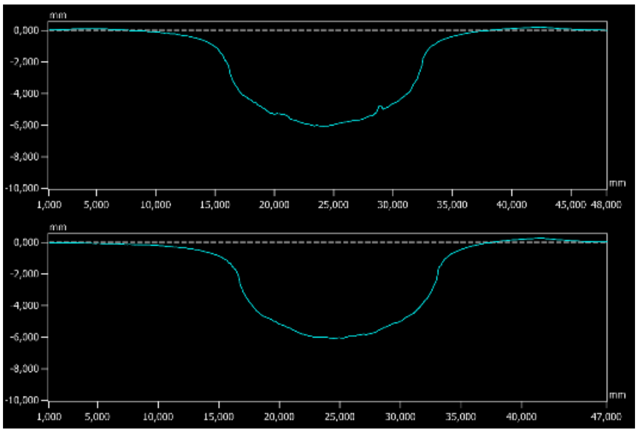

Table S16: SSRT results of 3 (Blast 2).

**1-Nitratomethyl-3,4-dinitropyrazole Blast 2**	
Measurement 1 [mm³]	1171,79
Measurement 2 [mm³]	1170,75
**Average Volume [mm³]**	**1171,27**

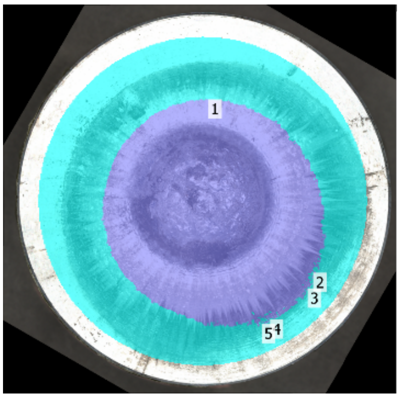

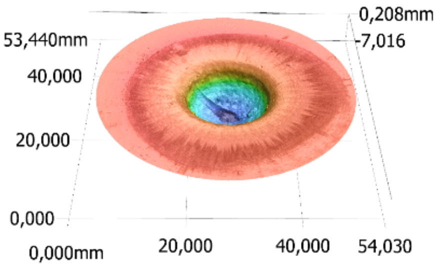

Table S17: SSRT results of 3 (Blast 3).

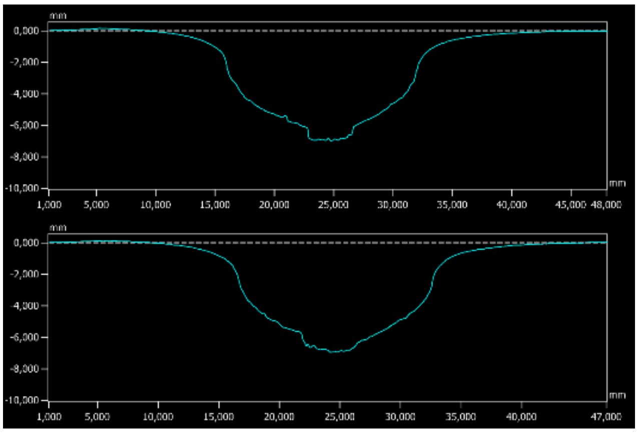

1-Nitratomethyl-3,4-dinitropyrazole Blast 3	
Measurement 1 [mm³]	1218,68
Measurement 2 [mm³]	1223,13
**Average Volume [mm³]**	**1220,90**

Evaluation of 1-nitratoethyl-3,4-dinitropyrazole (6)

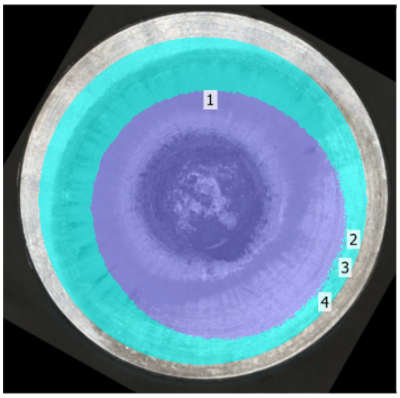

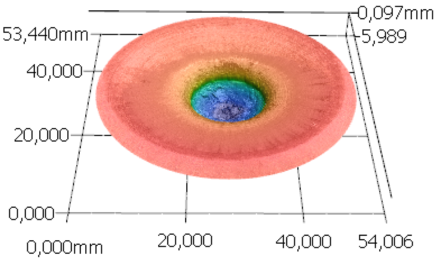

Table S18: SSRT results of 6 (Blast 1).

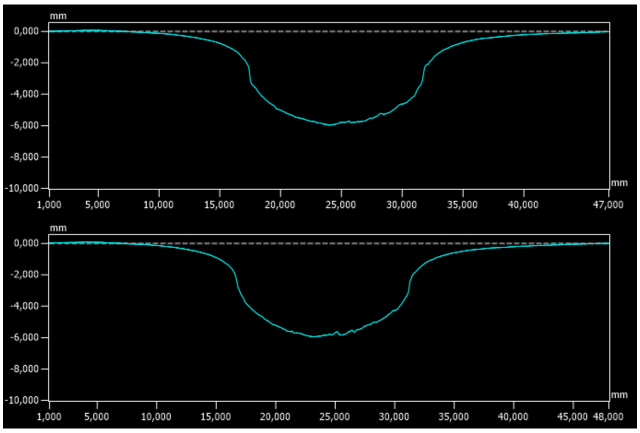

1-Nitratoethyl-3,4-dinitropyrazole Blast 1	
Measurement 1 [mm³]	1129,23
Measurement 2 [mm³]	1130,99
**Average Volume [mm³]**	**1130,11**

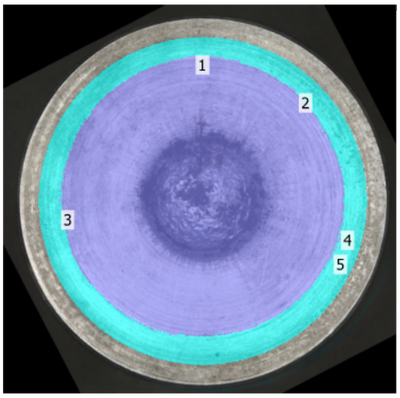

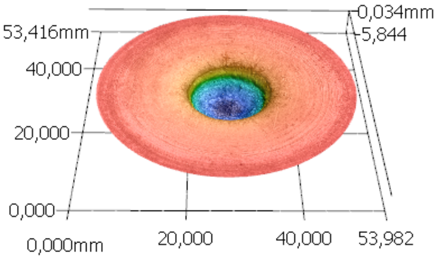

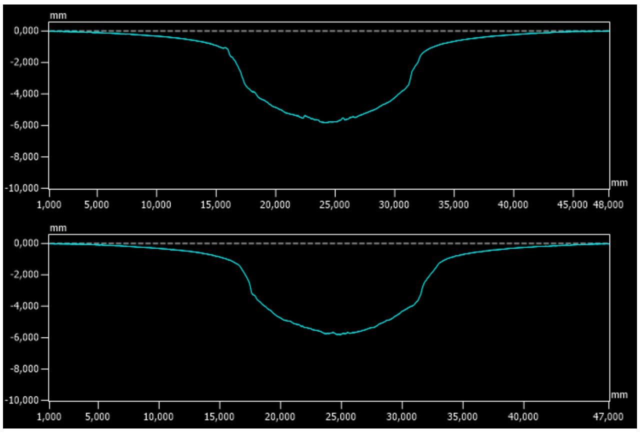

Table S19: SSRT results of 6 (Blast 2).

**1-Nitratoethyl-3,4-dinitropyrazole Blast 2**	
Measurement 1 [mm³]	1191,11
Measurement 2 [mm³]	1179,33
**Average Volume [mm³]**	**1185,22**

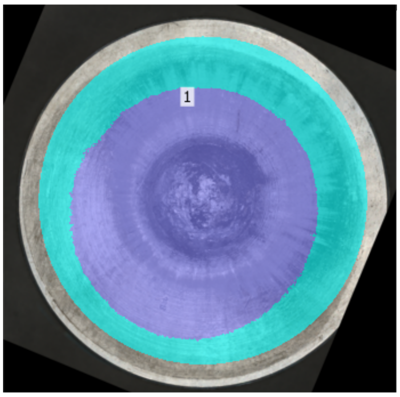

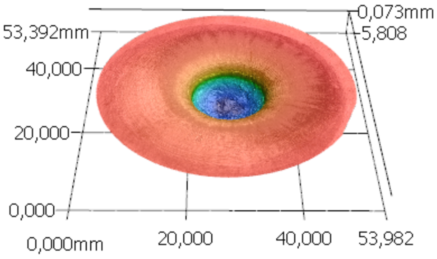

Table S20: SSRT results of 6 (Blast 3).

**1-Nitratoethyl-3,4-dinitropyrazole Blast 3**	
Measurement 1 [mm³]	1088,87
Measurement 2 [mm³]	1095,47
**Average Volume [mm³]**	**1092,17**

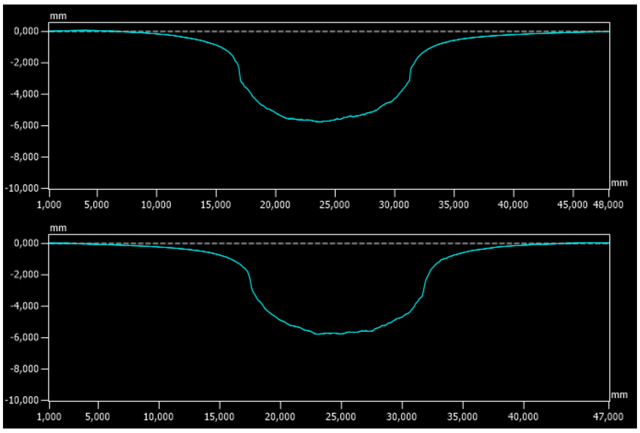

10. Experimental part and general methods

Caution! Nitropyrazoles are potentially energetic materials with sensitivities toward various stimuli. Therefore, meticulous security precautions (safety glass, face shield, earthed equipment and shoes, Kevlar gloves, and ear plugs) have to be applied while synthesizing and handling the described compounds.

All chemical reagents and solvents were employed as received (Sigma-Aldrich, Acros Organics, ABCR, TCI). NMR spectra were recorded on a 400 MHz instrument (*Bruker AV400* or *Bruker AV400TR*) at room temperature. Chemical shifts (δ) are reported in parts per million (ppm) and refer to tetramethylsilane (^{1}H, ^{13}C) and nitromethane (^{14}N, ^{15}N) (for more information see **Table S21**). All spectra were analyzed with the software MestReNOVA 10.0 from Mestrelab Research, S. L. Infrared (IR) spectra were recorded with a Perkin-Elmer Spektrum One FT-IR spectrometer in a range of 4000−400 cm^{-1}. Transmittance values are qualitatively described as “very strong” (vs), “strong” (s), “medium” (m), “weak” (w) and “very weak” (vw). Elemental analysis was carried out using an Elementar Vario el or Vario micro by pyrolysis of the sample and subsequent analysis of formed gases (allowed deviation for liquids: +/- 0.5%; for solids: +/- 0.3%). High-resolution mass spectra were recorded with a *Thermo Finnigan LTQ FT-ICR* (ESI) and a *Thermo Finnigan MAT 95* (EI) mass spectrometer. Melting and decomposition points were determined in a rage of 25−400 °C at a heating rate of 5 °C min^{-1} through differential thermal analysis (DTA) with an OZM Research DTA 552-Ex instrument. Thermogravimetric measurements were performed with a Perkin-Elmer TGA 4000 apparatus using a heating rate of 5 °C min^{-1} in a slow stream of nitrogen gas (1mL/min). The sensitivity data were determined using a BAM (Bundesanstalt für Materialforschung) drophammer[S15] according to STANAG 4489[S16] using a modified instruction[S17] and a BAM friction tester[S18] according to STANAG 4487[S19] using a modified instruction. The electrostatic sensitivity test was carried out using the OZM Electric Spark XSpark10 device[S20]. The classification is based on the 'UN Recommendations on the Transport of Dangerous Goods'.[S21]

Table S21: NMR pulse sequences and parameters used for the characterization of the new compounds.

	^{1}H	^{13}C	^{14}N	^{15}N	$^{1}H/^{15}N$ HMBC
**Pulse Sequence**	zg30	zgpg30	zgpg	zg30	hmbcqpndqf
**Number of Scans**	16	2048	5000	15000-39014	32
**Receiver Gain**	210	210	210	10-14	210
**Relaxation Delay (sec)**	4.0	0.5	0.2	3.0	2.0
**Pulse Width (µs)**	10.6	9.3	22.0	16.8	10.6
**Acuisition Time (sec)**	6.5	1.3	0.6	1.6	0.3
**NMR. Frenq.(MHz)**	400.18	100.64	28.91	40.56	400.18;40.54
**Spectral Width (Hz)**	10000.0	25252.5	14423.1	20380.4	2994.0;16233.8
**Acquired Size (DP)**	65536	32768	8192	32768	1024;256

1-Hydroxymethyl-3-nitropyrazole (1)[S22]

3-Nitropyrazole[S23] (6.00 g, 53.1 mmol, 1.0 eq.) was dissolved in MeOH (40 mL) and formaldehyde (40% aq., 10 mL, 133 mmol, 2.5 eq.) was added. The reaction mixture was stirred at room temperature overnight and put onto a mixture of water (50 mL) and sat. $NaHCO_3$ (30 mL). The solution was extracted with $CHCl_3$ (3 × 50 mL), dried over $MgSO_4$ and evaporated under reduced pressure. The crude product was recrystallized from $CHCl_3$ (~100 mL) to obtain 1-hydroxymethyl-3-nitropyrazole (**1**) (5.41 g, 37.8 mmol, 71%) as colourless crystalline solid.

DTA (5 °C min^{-1}) T_{melt} = 83 °C; **^{1}H NMR** (400 MHz, DMSO-*d*6, 25 °C): δ (ppm) = 8.08 (d, $^3J_{H,H}$ = 2.6 Hz, 1H), 7.30 (t, $^3J_{H,H}$ = 7.9 Hz, 1H), 7.05 (d, $^3J_{H,H}$ = 2.6 Hz, 1H), 5.48 (d, $^3J_{H,H}$ = 7.9 Hz, 2H); **^{13}C{^{1}H} NMR** (101 MHz, DMSO-*d*6, 25 °C): δ (ppm) = 155.4, 133.2, 102.9, 75.3; **^{14}N{^{1}H} NMR** (29 MHz, DMSO-*d*6, 25 °C): δ (ppm) = −20; **EA** ($C_4H_5N_3O_3$, 143.10 g/mol) calc. (found): C 33.57 (33.49), H 3.52 (3.28), N 29.36 (29.46) %; **HRMS** (EI+): *m/z* calculated for $C_4H_5N_3O_3$ [M]: 143.0331, found: 143.0324; **IR** (ATR, rel. int.): $\tilde{\nu}$ (cm^{-1}) = 3401 (m), 3211 (m), 3158 (m), 3147 (m), 3128 (m), 1537 (s), 1503 (s), 1456 (m), 1409 (m), 1379 (s), 1353 (s), 1284 (s), 1224 (m), 1176 (m), 1167 (s), 1084 (s), 1070 (s), 1050 (vs), 1034 (s), 1017 (m), 1006 (m), 998 (s), 983 (s)ö, 888 (w), 823 (s), 788 (s), 756 (s), 742 (vs), 651 (m), 613 (s), 466 (s), 418 (m).

1-Nitratomethyl-3-nitropyrazole (2)

To cooled acetic acid anhydride (3.0 mL, 31.7 mmol, 4.5 eq.), fuming nitric acid (1.5 mL, 35.9 mmol, 5.1 eq.) was added dropwise while keeping the temperature at 0 °C. To this mixture, 1-hydroxymethyl-3-nitropyrazole (**1**) (1.00 g, 6.99 mmol, 1.0 eq.) was added portion wise and stirred 15 min at this temperature. The reaction was stirred further at room temperature for 10 min and poured onto ice-water (~30 mL). The precipitate was filtered off, washed with a little amount of water and air dried at room temperature to give 1-nitratomethyl-3-nitropyrazole (**2**) (1.06 g, 5.64 mmol, 81%) as colourless powder.

DTA (5 °C min^{-1}) T_{melt} = 70 °C, T_{dec} = 161 °C; **^{1}H NMR** (400 MHz, DMSO-*d*6, 25 °C): δ (ppm) = 8.29 (d, $^3J_{H,H}$ = 2.7 Hz, 1H), 7.14 (d, $^3J_{H,H}$ = 2.7 Hz, 1H), 6.66 (s, 2H); **^{13}C{^{1}H} NMR** (101 MHz, DMSO-*d*6, 25 °C): δ (ppm) = 159.8, 136.6, 103.5, 79.1; **^{14}N{^{1}H} NMR** (29 MHz, DMSO-*d*6, 25 °C): δ (ppm) = −21,−48; **^{15}N NMR** (41 MHz, Aceton-*d*6, 25 °C): δ (ppm) = −22.4, −50.2 (t, $^3J_{N,H}$ = 5.4 Hz), −77.4 (t, $^3J_{N,H}$ = 2.8 Hz), −171.1 (dd, $^2J_{N,H}$ = 6.8, 4.4 Hz); **EA** ($C_4H_4N_4O_5$, 188.10 g/mol) calc. (found): C 25.54 (25.84), H 2.14 (2.18), N 29.79 (29.64) %; **HRMS** (EI+): *m/z* calculated for $C_4H_4N_4O_5$ [M]: 188.0182, found: 188.0181; **IR** (ATR, rel. int.): $\tilde{\nu}$ (cm^{-1}) = 3156 (w), 3137 (w), 3059 (w), 2938 (w), 1660 (vs), 1552 (s), 1539 (m), 1510 (s), 1451 (m), 1426 (m), 1396 (m), 1377 (m), 1347 (m), 1304 (s), 1283 (s), 1235 (s), 1180 (s), 1069 (m), 1041 (m), 1004 (m), 993 (m), 952 (s), 888 (vw), 831 (s), 821 (vs), 791 (s), 765 (vs), 755 (vs), 655 (s), 632 (s), 615 (s), 589 (m), 545 (m), 446 (s), 413 (m).

1-Nitratomethyl-3,4-dinitropyrazole (3)

Method A: 1-Hydroxymethyl-3-nitropyrazole (**1**) (3.00 g, 20.9 mmol, 1.0 eq.) was added at 0 °C to fuming nitric acid (15 mL, 359 mmol, 17 eq.) and stirred under ice cooling for 10 min. The reaction mixture was stirred for another 3.5 h at room temperature and poured onto ice-water (~100 mL). The precipitate was filtered off, washed with a little amount of water and air

dried at room temperature to give 1-nitratomethyl-3,4-dinitropyrazole (**3**) (2.00 g, 8.58 mmol, 41%) as colourless powder.

Method B: 1-Nitratomethyl-3-nitropyrazole (**2**) (0.50 g, 2.66 mmol, 1.0 eq.) was added at 0 °C to fuming nitric acid (4 mL, 95.9 mmol, 36 eq.) and stirred at under ice cooling for 10 min. The reaction mixture was stirred further 4 h at room temperature and poured onto ice-water (~20 mL). The precipitate was filtered off, washed with a little amount of water and air dried at room temperature to give 1-nitratomethyl-3,4-dinitropyrazole (**3**) (0.23 g, 0.98 mmol, 37%) as colourless powder.

DTA (5 °C min^{-1}) T_{melt} = 93 °C, T_{dec} = 159 °C; **^{1}H NMR** (400 MHz, DMSO-*d*6, 25 °C): δ (ppm) = 9.37 (s, 1H), 6.63 (s, 2H); **^{13}C{^{1}H} NMR** (101 MHz, DMSO-*d*6, 25 °C): δ (ppm) = 148.0, 136.2, 127.0, 78.9; **^{14}N{^{1}H} NMR** (29 MHz, DMSO-*d*6, 25 °C): δ (ppm) = −27,−51; **^{15}N NMR** (41 MHz, Aceton-*d*6, 25 °C): δ (ppm) = −28.1, −28.5, −52.2 (t, $^{3}J_{N,H}$ = 5.8 Hz), −80.7 (t, $^{3}J_{N,H}$ = 2.7 Hz), −177.9 (d, $^{2}J_{N,H}$ = 3.0 Hz); **EA** ($C_4H_3N_5O_7$, 233.10 g/mol) calc. (found): C 20.61 (20.74), H 1.30 (1.51), N 30.05 (29.36) %; **HRMS** (EI+): *m/z* calculated for $C_4H_3N_5O_7$ [M]: 233.0032, found: 233.0027; **IR** (ATR, rel. int.): $\tilde{\nu}$ (cm^{-1}) = 3169 (w), 3156 (m), 3067 (w), 1648 (s), 1556 (s), 1522 (s), 1460 (m), 1428 (m), 1384 (m), 1364 (s), 1346 (s), 1312 (s), 1282 (vs), 1236 (m), 1145 (vs), 1121 (m), 1056 (w), 1008 (w), 959 (s), 863 (s), 833 (vs), 807 (vs), 766 (s), 754 (s), 741 (s), 683 (m), 663 (s), 622 (s), 600 (m), 583 (s), 496 (s), 430 (m).

1-Hydroxyethyl-3-nitropyrazole (4)[S24]

3-Nitropyrazole[S23] (4.00 g, 35.4 mmol, 1.0 eq.) was dissolved in THF (40 mL). Potassium carbonate (9.78 g, 70.8 mmol, 2.0 eq.) and 2-bromoethanol (3.8 mL, 6.70 g, 53.6 mmol, 1.5 eq.) were added and the mixture was refluxed at 70 °C for 24 h. The solvent of the resulting suspension was evaporated under reduced pressure and water (40 mL) and ethyl acetate (40 ml) was added to the solid residue. The aqueous phase was extracted with ethyl acetate (2 × 40 mL) and the combined organic phases were dried over $MgSO_4$, discolored with activated carbon and evaporated. The crude compound solidified over the course of up to 3 d. For purification the residue was triturated with diethyl ether, filtrated, washed with diethyl ether and air dried at room temperature to give 1-hydroxyethyl-3-nitropyrazole (**4**) (4.00 g, 24.5 mmol, 72%) as colourless solid.

DTA (5 °C min^{-1}) T_{melt} = 72 °C; **^{1}H NMR** (400 MHz, DMSO-*d*6, 25 °C): δ (ppm) = 7.99 (d, $^{3}J_{H,H}$ = 2.5 Hz, 1H), 7.02 (d, $^{3}J_{H,H}$ = 2.5 Hz, 1H), 4.99 (t, $^{3}J_{H,H}$ = 5.3 Hz, 1H), 4.27 (t, $^{3}J_{H,H}$ = 5.2 Hz, 2H), 3.78 (q, $^{3}J_{H,H}$ = 5.3 Hz, 2H); **^{13}C{^{1}H} NMR** (101 MHz, DMSO-*d*6, 25 °C): δ (ppm) = 155.0, 134.5, 102.5, 59.5, 55.7; **^{14}N{^{1}H} NMR** (29 MHz, DMSO-*d*6, 25 °C): δ (ppm) = −20; **EA** ($C_5H_7N_3O_3$, 157.13 g/mol) calc. (found): C 38.22 (38.26), H 4.49 (4.24), N 26.74 (26.44) %; **HRMS** (EI+): *m/z* calculated for $C_5H_8N_3O_3$ $[M]^+$: 158.0560, found: 158.0560; **IR** (ATR, rel. int.): $\tilde{\nu}$ (cm^{-1}) = 3413 (s), 3162 (m), 3114 (m), 1533 (s), 1499 (s), 1460 (m), 1452 (m), 1433 (m), 1407 (m), 1386 (s), 1371 (s), 1359 (s), 1293 (vs), 1253 (m), 1193 (s), 1160 (m), 1080 (m), 1061 (s), 1039 (s), 1012 (m), 1005 (s), 947 (m), 865 (m), 823 (s), 788 (vs), 754 (vs), 676 (w), 657 (w), 622 (m), 576 (m), 538 (m), 500 (m), 426 (m).

1-Nitratoethyl-3-nitropyrazole (5)

To cooled acetic acid anhydride (4.0 mL, 42.3 mmol, 6.7 eq.) fuming nitric acid (2.0 mL, 47.9 mmol, 7.5 eq.) was added dropwise while keeping the temperature at 0 °C. To this mixture 1-hydroxyethyl-3-nitropyrazole (**4**) (1.00 g, 6.36 mmol, 1.0 eq.) was added portion

wise and stirred for 10 min at this temperature. The reaction was stirred further at room temperature until everything was dissolved (approx. 15 min) and poured onto ice-water (~40 mL). The precipitate was filtered off, washed with a little amount of water and air dried at room temperature to give 1-nitratoethyl-3-nitropyrazole (**5**) (1.16 g, 5.74 mmol, 90%) as colourless powder.

DTA (5 °C min^{-1}) T_{melt} = 78 °C, T_{dec} = 198 °C; **^{1}H NMR** (400 MHz, DMSO-*d*6, 25 °C): δ (ppm) = 8.11 (d, $^{3}J_{H,H}$ = 2.6 Hz, 1H), 7.07 (d, $^{3}J_{H,H}$ = 2.5 Hz, 1H), 4.95 (t, $^{3}J_{H,H}$ = 5.0 Hz, 2H), 4.67 (t, $^{3}J_{H,H}$ = 4.9 Hz, 2H); **^{13}C{^{1}H} NMR** (101 MHz, DMSO-*d*6, 25 °C): δ (ppm) = 155.4, 134.8, 103.0, 71.1, 50.2; **^{14}N{^{1}H} NMR** (29 MHz, DMSO-*d*6, 25 °C): δ (ppm) = −21,−44; **EA** ($C_5H_6N_4O_5$, 202.13 g/mol) calc. (found): C 29.71 (29.51), H 2.99 (3.04), N 27.72 (27.18) %; **HRMS** (EI+): *m/z* calculated for $C_5H_6N_4O_5$ [M]: 202.0338, found: 202.0333; **IR** (ATR, rel. int.): $\tilde{\nu}$ (cm^{-1}) = 3179 (w), 3130 (m), 1645 (s), 1628 (s), 1550 (m), 1536 (s), 1507 (m), 1470 (w), 1457 (m), 1429 (m), 1382 (s), 1358 (s), 1301 (s), 1279 (vs), 1258 (s), 1206 (w), 1181 (m), 1089 (w), 1059 (m), 1028 (s), 1000 (m), 961 (w), 892 (s), 878 (s), 856 (s), 823 (s), 788 (s), 755 (vs), 709 (s), 690 (m), 648 (w), 611 (m), 566 (m), 542 (w), 498 (m), 431 (w).

1-Nitratoethyl-3,4-dinitropyrazole (6)

Method A: 1-Hydroxyethyl-3-nitropyrazole (**4**) (1.00 g, 6.36 mmol, 1.0 eq.) was added at 0 °C to fuming nitric acid (4 mL, 95.9 mmol, 15 eq.) and stirred under ice cooling for 10 min. The reaction mixture was stirred further 3.5 h at room temperature, poured onto ice water (~40 mL), and stored at 4 °C for 2 h for complete precipitation. The precipitate was filtered off, washed with a little amount of water, and air dried at room temperature to give 1-nitratoethyl-3,4-dinitropyrazole (**6**) (1.47 g, 5.95 mmol, 94%) as colourless powder.

Method B: 1-Nitratoethyl-3-nitropyrazole (**5**) (0.96 g, 4.75 mmol, 1.0 eq.) was added at 0 °C to fuming nitric acid (8 mL, 192 mmol, 40 eq.) and stirred at under ice-cooling for 15 min. The reaction mixture was stirred further 4 h at room temperature and poured onto ice-water (~40 mL). The precipitate was filtered off, washed with a little amount of water and air dried at room temperature to give 1-nitratomethyl-3,4-dinitropyrazole (**3**) (0.86 g, 3.48 mmol, 73%) as colourless powder.

DTA (5 °C min^{-1}) T_{melt} = 61 °C, T_{dec} = 198 °C; **^{1}H NMR** (400 MHz, DMSO-*d*6, 25 °C): δ (ppm) = 9.23 (s, 1H), 4.96 (t, $^{3}J_{H,H}$ = 4.6 Hz, 2H), 4.70 (t, $^{3}J_{H,H}$ = 4.6 Hz, 2H); **^{13}C{^{1}H} NMR** (101 MHz, DMSO-*d*6, 25 °C): δ (ppm) = 147.2, 134.7, 126.3, 70.4 51.1; **^{14}N{^{1}H} NMR** (29 MHz, DMSO-*d*6, 25 °C): δ (ppm) = −26,−43; **EA** ($C_5H_5N_5O_7$, 247.12 g/mol) calc. (found): C 24.30 (24.24), H 2.04 (1.92), N 28.34 (28.04) %; **HRMS** (EI+): *m/z* calculated for $C_5H_5N_5O_7$ [M]: 247.0189, found: 247.0185; **IR** (ATR, rel. int.): $\tilde{\nu}$ (cm^{-1}) = 3157 (w), 1639 (s), 1545 (s), 1515 (s), 1476 (w), 1460 (m), 1434 (m), 1412 (w), 1358 (s), 1337 (s), 1308 (m), 1281 (vs), 1248 (m), 1195 (w), 1150 (m), 1122 (w), 1081 (w), 1030 (m), 1020 (m), 966 (w), 902 (s), 862 (s), 808 (s), 759 (s), 750 (s), 715 (m), 706 (w), 667 (w), 641 (w), 596 (m), 574 (m), 504 (w), 455 (w).

1-Hydroxymethyl-4-nitropyrazole (7)

4-Nitropyrazole[S25] (3.00 g, 26.5 mmol, 1.0 eq.) was dissolved in MeOH (20 mL) and formaldehyde (40% aq., 5 mL, 66.6 mmol, 2.5 eq.) was added. The reaction mixture was stirred at room temperature overnight and poured onto a mixture of water (25 mL) and sat. $NaHCO_3$ (15 mL). The solution was extracted with $CHCl_3$ (3 × 30 mL), dried over $MgSO_4$, and evaporated under reduced pressure. The crude product was recrystallized from $CHCl_3$

(~70 mL) to obtain 1-hydroxymethyl-4-nitropyrazole (**7**) (2.10 g, 14.7 mmol, 55%) as colourless crystalline solid.

DTA (5 °C min^{-1}) T_{melt} = 98 °C; **^{1}H NMR** (400 MHz, DMSO-*d*6, 25 °C): δ (ppm) = 8.89 (d, $^{4}J_{H,H}$ = 0.7 Hz, 1H), 8.28 (d, $^{4}J_{H,H}$ = 0.8 Hz, 1H), 7.21 (t, $^{3}J_{H,H}$ = 7.8 Hz, 1H), 5.43 (d, $^{3}J_{H,H}$ = 7.8 Hz, 2H); **^{13}C{^{1}H} NMR** (101 MHz, DMSO-*d*6, 25 °C): δ (ppm) = 135.7, 135.3, 129.8, 74.9; **^{14}N{^{1}H} NMR** (29 MHz, DMSO-*d*6, 25 °C): δ (ppm) = −18; **EA** ($C_4H_5N_3O_3$, 143.10 g/mol) calc. (found): C 33.57 (33.59), H 3.52 (3.58), N 29.36 (28.71) %; **HRMS** (EI+): *m/z* calculated for $C_4H_5N_3O_3$ [M]: 143.0331, found: 143.0309; **IR** (ATR, rel. int.): $\tilde{\nu}$ (cm^{-1}) = 3261 (m), 3139 (m), 3109 (m), 1557 (w), 1536 (m), 1514 (s), 1481 (m), 1457 (m), 1411 (s), 1400 (m), 1356 (w), 1326 (s), 1311 (m), 1293 (s), 1213 (w), 1183 (w), 1114 (m), 1071 (vs), 1028 (m), 1000 (m), 978 (s), 897 (m), 863 (m), 816 (s), 749 (vs), 689 (m), 650 (m), 596 (s), 552 (m), 482 (m).

1-Hydroxyethyl-4-nitropyrazole (8)

4-Nitropyrazole[S25] (4.00 g, 35.4 mmol, 1.00 eq.) was dissolved in THF (40 mL). Potassium carbonate (9.78 g, 70.8 mmol, 2.0 eq.) and 2-bromoethanol (3.8 mL, 6.70 g, 53.6 mmol, 1.5 eq.) were added and the mixture was refluxed at 70 °C for 24 h. The solvent of the resulting suspension was evaporated under reduced pressure and water (40 mL) and ethyl acetate (40 ml) was added to the solid residue. The aqueous phase was extracted with ethyl acetate (2 × 40 mL) and combined organic phases were dried over $MgSO_4$, discolored with activated carbon and evaporated. For purification, the residue was triturated with diethyl ether, filtrated, washed with diethyl ether and air dried at room temperature to give 1-hydroxyethyl-4-nitropyrazole (**8**) (2.80 g, 17.8 mmol, 50%) as colourless solid.

DTA (5 °C min^{-1}) T_{melt} = 92 °C; **^{1}H NMR** (400 MHz, DMSO-*d*6, 25 °C): δ (ppm) = 8.80 (d, $^{4}J_{H,H}$ = 0.8 Hz, 1H), 8.25 (d, $^{4}J_{H,H}$ = 0.7 Hz, 1H), 4.99 (t, $^{3}J_{H,H}$ = 5.4 Hz, 1H), 4.22 (t, $^{3}J_{H,H}$ = 5.4 Hz, 2H), 3.77 (q, $^{3}J_{H,H}$ = 5.4 Hz, 2H); **^{13}C{^{1}H} NMR** (101 MHz, DMSO-*d*6, 25 °C): δ (ppm) = 135.5, 134.8, 130.9, 59.2 55.2; **^{14}N{^{1}H} NMR** (29 MHz, DMSO-*d*6, 25 °C): δ (ppm) = −19; **EA** ($C_5H_7N_3O_3$, 157.13 g/mol) calc. (found): C 38.22 (38.33), H 4.49 (4.34), N 26.74 (26.35) %; **HRMS** (EI+): *m/z* calculated for $C_5H_8N_3O_3$ $[M]^+$: 158.0560, found: 158.0558; **IR** (ATR, rel. int.): $\tilde{\nu}$ (cm^{-1}) = 3379 (m), 3140 (m), 3123 (m), 1559 (w), 1531 (s), 1504 (vs), 1485 (s), 1438 (m), 1429 (s), 1408 (vs), 1376 (m), 1364 (m), 1312 (vs), 1296 (vs), 1208 (m), 1162 (w), 1152 (m), 1127 (s), 1069 (s), 1045 (s), 1002 (s), 991 (m), 943 (m), 890 (s), 871 (m), 849 (m), 818 (vs), 756 (vs), 662 (w), 610 (s), 594 (vs), 556 (s), 511 (m), 423 (m).

1-Nitratoethyl-4-nitropyrazole (9)

To cooled acetic acid anhydride (4.0 mL, 42.3 mmol, 6.7 eq.) fuming nitric (2.0 mL, 47.9 mmol, 7.5 eq.) was added dropwise while keeping the temperature at 0 °C. To this mixture, 1-hydroxyethyl-4-nitropyrazole (**8**) (1.00 g, 6.36 mmol, 1.0 eq.) was added portion wise and stirred 20 min at this temperature. The reaction was stirred further 10 min at room temperature and poured onto ice-water (~40 mL). The precipitate was filtered off, washed with a little amount of water and air dried at room temperature to give 1-nitratoethyl-4-nitropyrazole (**9**) (1.01 g, 5.00 mmol, 77%) as colourless powder.

DTA (5 °C min^{-1}) T_{melt} = 52 °C, T_{dec} = 191 °C; **^{1}H NMR** (400 MHz, DMSO-*d*6, 25 °C): δ (ppm) = 8.98 (d, $^{4}J_{H,H}$ = 0.7 Hz, 1H), 8.31 (d, $^{4}J_{H,H}$ = 0.7 Hz, 1H), 4.94 (t, $^{3}J_{H,H}$ = 4.9 Hz, 2H), 4.60 (t, $^{3}J_{H,H}$ = 5.0 Hz, 2H); **^{13}C{^{1}H} NMR** (101 MHz, DMSO-*d*6, 25 °C): δ (ppm) = 136.0, 135.1, 131.2, 70.9, 49.8; **^{14}N{^{1}H} NMR** (29 MHz, DMSO-*d*6, 25 °C): δ (ppm) = −19,−43; **EA** ($C_5H_6N_4O_5$,

202.13 g/mol) calc. (found): C 29.71 (29.79), H 2.99 (2.71), N 27.72 (27.46) %; **HRMS** (EI+): *m/z* calculated for $C_5H_6N_4O_5$ [M]: 202.0338, found: 202.0333; **IR** (ATR, rel. int.): $\tilde{\nu}$ (cm^{-1}) = 3134 (w), 3122 (m), 1629 (s), 1554 (w), 1529 (m), 1505 (s), 1481 (m), 1439 (m), 1422 (m), 1407 (s), 1361 (m), 1313 (s), 1297 (s), 1276 (vs), 1200 (w), 1183 (w), 1132 (m), 1079 (m), 1029 (w), 1008 (m), 1001 (s), 978 (w), 946 (w), 879 (s), 838 (s), 818 (s), 753 (s), 712 (w), 693 (m), 659 (w), 640 (m), 597 (m), 550 (m), 497 (m), 420 (m).

1-Chloromethyl-3-nitropyrazole (10)[S26]

Thionyl chloride (3 mL, 41.3 mmol, 2.1 eq.) dissolved in DCM (10 mL) was added dropwise to a cooled solution of 1-hydroxymethyl-3-nitropyrazole (**1**) (2.85 g, 19.9 mmol, 1.0 eq.) in DCM (20 mL). The reaction mixture was stirred 1 h at 5 °C, then overnight at room temperature and evaporated under reduced pressure. The crude product was purified via column chromatography (*i*Hex/EtOAc 3 : 2, R_f = 0.5) to give 1-chloromethyl-3-nitropyrazole (**10**) (2.91 g, 18.0 mmol, 90%) as an colourless to yellowish solid. (*Note: Crude product can be used for the conversion to compound **14** which can be purified via column chromatography in the next step.*)

DTA (5 °C min^{-1}) T_{melt} = 25 °C; **^{1}H NMR** (400 MHz, DMSO-*d*6, 25 °C): δ (ppm) = 8.29 (d, $^3J_{H,H}$ = 2.7 Hz, 1H), 7.10 (d, $^3J_{H,H}$ = 2.7 Hz, 1H), 6.26 (s, 2H); **^{13}C{^{1}H} NMR** (101 MHz, DMSO-*d*6, 25 °C): δ (ppm) = 156.5, 135.6, 104.0, 58.7; **^{14}N{^{1}H} NMR** (29 MHz, DMSO-*d*6, 25 °C): δ (ppm) = −22; **EA** ($C_4H_4ClN_3O_2$, 161.55 g/mol) calc. (found): C 29.74 (30.59), H 2.50 (2.71), N 26.01 (25.14) %; **HRMS** (EI+): *m/z* calculated for $C_4H_4ClN_3O_2$ [M]: 160.9992, found: 161.0052; **IR** (ATR, rel. int.): $\tilde{\nu}$ (cm^{-1}) = 3157 (w), 3133 (w), 3055 (vw), 1729 (w), 1549 (s), 1510 (s), 1454 (m), 1387 (s), 1352 (m), 1292 (vs), 1247 (m), 1189 (s), 1158 (w), 1100 (w), 1054 (m), 996 (m), 939 (vw), 822 (s), 781 (m), 752 (s), 729 (vs), 718 (s), 642 (m), 610 (w), 538 (w), 441 (w).

1-Azidomethyl-3-nitropyrazole (11)

To a solution of 1-chloromethyl-3-nitropyrazole (**10**) (1.5 g, 9.29 mmol, 1.0 eq.) in DMF (75 mL), sodium azide (0.72 g, 11.1 mmol, 1.2 eq.) was added and the reaction mixture was heated at 80 °C for at least 18 h. The solvent was evaporated under reduced pressure and the residue was taken up in water (~70 mL). The resulting solution was extracted with EtOAc (3 × 50 mL), dried over $MgSO_4$ and evaporated under reduced pressure to give 1-azidomethyl-3-nitropyrazole (**11**) (1.34 g, 7.97 mmol, 86%) as a yellowish solid.

DTA (5 °C min^{-1}) T_{melt} = 40 °C, T_{dec} = 179 °C; **^{1}H NMR** (400 MHz, DMSO-*d*6, 25 °C): δ (ppm) = 8.22 (d, $^3J_{H,H}$ = 2.6 Hz, 1H), 7.13 (d, $^3J_{H,H}$ = 2.7 Hz, 1H), 5.74 (s, 2H); **^{13}C{^{1}H} NMR** (101 MHz, DMSO-*d*6, 25 °C): δ (ppm) = 156.2, 134.9, 103.4, 65.6; **^{14}N{^{1}H} NMR** (29 MHz, DMSO-*d*6, 25 °C): δ (ppm) = −20, −135, −161; **EA** ($C_4H_4N_6O_2$, 168.12 g/mol) calc. (found): C 28.58 (28.88), H 2.40 (2.69), N 49.99 (49.69) %; **HRMS** (EI+): *m/z* calculated for $C_4H_4N_6O_2$ [M]: 168.0396, found: 168.0391; **IR** (ATR, rel. int.): $\tilde{\nu}$ (cm^{-1}) = 3160 (w), 3129 (m), 2150 (m), 2116 (s), 2086 (s), 1545 (m), 1502 (m), 1454 (m), 1386 (s), 1370 (s), 1346 (m), 1288 (vs), 1236 (s), 1202 (s), 1174 (s), 1119 (w), 1086 (w), 1058 (s), 1030 (m), 997 (m), 989 (m), 906 (m), 823 (s), 790 (s), 751 (vs), 665 (m), 645 (m), 615 (w), 565 (m), 540 (w), 445 (m).

1-Chloromethyl-4-nitropyrazole (12)[S26]

Thionyl chloride (3 mL, 41.3 mmol, 2.1 eq.) dissolved in DCM (10 mL) was added dropwise to a cooled solution of 1-hydroxymethyl-4-nitropyrazole (**7**) (2.85 g, 19.9 mmol, 1.0 eq.) in DCM

(20 mL). The reaction mixture was stirred 1 h at 5 °C, then overnight at room temperature and evaporated under reduced pressure. The crude product was purified via column chromatography (*i*Hex/EtOAc 3 : 2, R_f = 0.7) to give 1-chloromethyl-4-nitropyrazole (**12**) (2.80 g, 17.3 mmol, 87%) as an colourless to yellowish solid.

DTA (5 °C min^{-1}) T_{melt} = 38 °C; **^{1}H NMR** (400 MHz, DMSO-*d*6, 25 °C): δ (ppm) = 9.15 (d, $^{4}J_{H,H}$ = 0.7 Hz, 1H), 8.40 (d, $^{4}J_{H,H}$ = 0.7 Hz, 1H), 6.18 (s, 2H); **^{13}C{^{1}H} NMR** (101 MHz, DMSO-*d*6, 25 °C): δ (ppm) = 137.3, 136.0, 131.9, 58.5; **^{14}N{^{1}H} NMR** (29 MHz, DMSO-*d*6, 25 °C): δ (ppm) = −20; **EA** ($C_4H_4ClN_3O_2$, 161.55 g/mol) calc. (found): C 29.74 (29.45), H 2.50 (2.51), N 26.01 (25.77) %; **HRMS** (EI+): *m/z* calculated for $C_4H_4ClN_3O_2$ [M]: 160.9992, found: 161.0003; **IR** (ATR, rel. int.): $\tilde{\nu}$ (cm^{-1}) = 3125 (m), 3046 (m), 2987 (w), 1553 (m), 1533 (s), 1514 (s), 1498 (s), 1468 (m), 1448 (m), 1406 (s), 1353 (m), 1326 (s), 1310 (s), 1295 (s), 1159 (m), 1133 (s), 1001 (s), 984 (m), 935 (m), 887 (s), 863 (w), 817 (s), 752 (s), 729 (s), 712 (vs), 650 (s), 594 (s), 548 (s), 444 (m).

1-Azidomethyl-4-nitropyrazole (13)

To a solution of 1-chloromethyl-3-nitropyrazole (**10**) (1.00 g, 6.19 mmol, 1.0 eq.) in DMF (50 mL) sodium azide (0.61 g, 9.38 mmol, 1.5 eq.) was added and the reaction mixture was heated at 80 °C for min. 18 h. The solvent was evaporated under reduced pressure and the residue was absorbed in water (~50 mL). The resulting solution was extracted with EtOAc (3 × 30 mL), dried over $MgSO_4$ and evaporated under reduced pressure to give 1-azidomethyl-3-nitropyrazole (**13**) (0.91 g, 5.41 mmol, 87%) as a yellowish solid.

DTA (5 °C min^{-1}) T_{melt} = 42 °C, T_{dec} = 174 °C; **^{1}H NMR** (400 MHz, DMSO-*d*6, 25 °C): δ (ppm) = 9.06 (d, $^{4}J_{H,H}$ = 0.7 Hz, 1H), 8.42 (d, $^{4}J_{H,H}$ = 0.8 Hz, 1H), 5.65 (s, 2H); **^{13}C{^{1}H} NMR** (101 MHz, DMSO-*d*6, 25 °C): δ (ppm) = 136.9, 135.6, 131.2, 65.3; **^{14}N{^{1}H} NMR** (29 MHz, DMSO-*d*6, 25 °C): δ (ppm) = −18, −135, −160; **EA** ($C_4H_4N_6O_2$, 168.12 g/mol) calc. (found): C 28.58 (28.57), H 2.40 (2.48), N 49.99 (50.00) %; **HRMS** (EI+): *m/z* calculated for $C_4H_4N_6O_2$ [M]: 168.0396, found: 168.0390; **IR** (ATR, rel. int.): $\tilde{\nu}$ (cm^{-1}) = 3120 (s), 3028 (w), 2164 (m), 2129 (s), 2100 (m), 1557 (w), 1531 (s), 1505 (vs), 1468 (s), 1454 (m), 1424 (m), 1407 (s), 1376 (s), 1321 (s), 1285 (vs), 1245 (vs), 1204 (s), 1187 (m), 1130 (s), 1045 (m), 998 (s), 970 (s), 910 (s), 886 (s), 857 (m), 817 (vs), 753 (s), 740 (s), 668 (m), 645 (m), 590 (s), 554 (s), 468 (m).

1-Chloromethyl-3,4-dinitropyrazole (14)[S23]

1-Chloromethyl-3-nitropyrazole (10) (2.00 g, 18.6 mmol, 1.0 eq.) was added at 0 °C to fuming nitric acid (12 mL, 288 mmol, 15 eq.) and stirred under ice cooling for 10 min. The reaction mixture was stirred further 3 h at room temperature, poured onto ice-water (~80 mL) and extracted with EtOAc (3 × 50 mL). The combined organic phases were dried over $MgSO_4$ and the solvent was evaporated under reduced pressure. The crude product was purified via column chromatography (*i*Hex/EtOAc 3 : 7, R_f = 0.7) to give 1-chloromethyl-3,4-dinitropyrazole (**14**) (2.26 g, 10.9 mmol, 59%) as an yellowish oil.

^{1}H NMR (400 MHz, DMSO-*d*6, 25 °C): δ (ppm) = 9.38 (s, 1H), 6.25 (s, 2H); **^{13}C{^{1}H} NMR** (101 MHz, DMSO-*d*6, 25 °C): δ (ppm) = 147.8, 135.3, 127.0, 58.8; **^{14}N{^{1}H} NMR** (29 MHz, DMSO-*d*6, 25 °C): δ (ppm) = −27; **EA** ($C_4H_3ClN_4O_4$, 206.54 g/mol) calc. (found): C 23.26 (23.61), H 1.46 (1.52), N 27.13 (27.08) %; **HRMS** (EI+): *m/z* calculated for $C_4H_3ClN_4O_4$ [M]: 205.9843, found: 205.9836; **IR** (ATR, rel. int.): $\tilde{\nu}$ (cm^{-1}) = 3139 (w), 3060 (vw), 1545 (s), 1516 (vs), 1463

(m), 1418 (w), 1362 (s), 1339 (s), 1296 (s), 1171 (w), 1141 (s), 1119 (m), 1010 (w), 946 (vw), 861 (s), 807 (vs), 743 (s), 731 (s), 624 (w), 594 (w), 479 (m), 416 (vw).

1-Azidomethyl-3,4-dinitropyrazole (15)

To a solution of 1-chloromethyl-3,4-dinitropyrazole (**14**) (1.86 g, 9.01 mmol, 1.0 eq.) in DMF (75 mL) sodium azide (0.88 g, 13.5 mmol, 1.5 eq.) was added. The reaction mixture was then heated at 80 °C for at least 18 h. Upon cooling to room temperature, the reaction was poured onto ice water (~75 mL) and extracted with EtOAc (3 × 50 mL). The combined organic phases were subsequently washed with $NaHCO_3$ (9% aq. 50 mL) and LiCl (10% aq. 50 mL), followed by drying over $MgSO_4$ and evaporation of the solvent to give a mixture of 1-azidomethyl-3,4-dinitropyrazole (**15**) and 1-azidomethyl-3-azido-4-nitropyrazole (**16**) as yellow oil. Compound **15** can be isolated via column chromatography (*i*Hex:EtOAc 7 : 3, R_f = 0.3) as yellowish oil (0.48 g, 2.27 mmol, 25%).

DTA (5 °C min^{-1}) T_{dec} = 149 °C; **^{1}H NMR** (400 MHz, DMSO-*d*6, 25 °C): δ (ppm) = 9.30 (s, 1H), 5.75 (s, 2H); **^{13}C{^{1}H} NMR** (101 MHz, DMSO-*d*6, 25 °C): δ (ppm) = 140.3, 134.5, 126.6, 66.3; **^{14}N{^{1}H} NMR** (29 MHz, DMSO-*d*6, 25 °C): δ (ppm) = −27,−137; **EA** ($C_4H_3N_7O_4$, 213.02 g/mol) calc. (found): C 22.54 (22.55), H 1.42 (1.73), N 46.01 (45.70) %; **IR** (ATR, rel. int.): $\tilde{\nu}$ (cm^{-1}) = 3139 (w), 2119 (s), 1542 (s), 1516 (vs), 1462 (m), 1361 (s), 1341 (s), 1300 (s), 1237 (s), 1198 (m), 1139 (s), 1119 (m), 1035 (w), 1005 (w), 913 (m), 861 (s), 807 (vs), 745 (s), 666 (w), 626 (w), 598 (w), 561 (w), 491 (m).

1-Azidomethyl-3-azido-4-nitropyrazole (16)

To a solution of 1-chloromethyl-3,4-dinitropyrazole (**14**) (0.50 g, 2.42 mmol, 1.0 eq.) in DMF (25 mL) sodium azide (0.39 g, 6.05 mmol, 2.5 eq.) was added. The reaction mixture was then heated at 80 °C for at least 18 h. Upon cooling to room temperature, the reaction was poured onto ice water (~25 mL) and extracted with EtOAc (3 × 30 mL). The combined organic phases were subsequently washed with $NaHCO_3$ (9% aq. 30 mL) and LiCl (10% aq. 40 mL), followed by drying over $MgSO_4$ and evaporation of the solvent to give 1-azidomethyl-3-azido-4-nitropyrazole (**16**) (0.44 g, 2.09 mmol, 86%) as yellowish solid. If desired, the obtained compound can be further purified via column chromatography (EtOAc, R_f = 0.7).

DTA (5 °C min^{-1}) T_{melt} = 50 °C, T_{dec} = 157 °C; **^{1}H NMR** (400 MHz, DMSO-*d*6, 25 °C): δ (ppm) = 9.06 (s, 1H), 5.58 (s, 2H); **^{13}C{^{1}H} NMR** (101 MHz, DMSO-*d*6, 25 °C): δ (ppm) = 143.4, 133.9, 125.1, 65.5; **^{14}N{^{1}H} NMR** (29 MHz, DMSO-*d*6, 25 °C): δ (ppm) = −20,−135,−143; **^{15}N NMR** (41 MHz, DMSO-*d*6, 25 °C): δ (ppm) = −22.2, −100.2 (t, $^3J_{N,H}$ = 2.6 Hz),−135.6 (t, $^2J_{N,H}$ = 5.0 Hz),−143.7,−145.4,−160.9,−177.1 (d, $^2J_{N,H}$ = 2.8 Hz), −296.3,−301.6 (t, $^2J_{N,H}$ = 2.5 Hz); **EA** ($C_4H_3N_9O_2$, 209.13 g/mol) calc. (found): C 22.97 (23.85), H 1.45 (1.78), N 60.28 (59.55) %; **HRMS** (EI+): *m/z* calculated for $C_4H_3N_9O_2$ [M]: 209.0410, found: 209.0402; **IR** (ATR, rel. int.): $\tilde{\nu}$ (cm^{-1}) = 3145 (m), 3042 (w), 2976 (w), 2165 (m), 2134 (vs), 2096 (s), 1536 (s), 1504 (vs), 1474 (vs), 1460 (s), 1441 (m), 1361 (vs), 1307 (s), 1250 (s), 1219 (vs), 1133 (s), 1107 (s), 1033 (m), 995 (m), 908 (s), 871 (s), 852 (s), 812 (w), 792 (s), 757 (s), 736 (s), 680 (m), 666 (s), 621 (vs), 608 (vs), 559 (s), 533 (s), 504 (m), 463 (m).

1-Azidoethyl-3-nitropyrazole (17)

To a solution of 3-nitropyrazole[S23] (2.00 g, 17.7 mmol, 1.0 eq) in DMF (20 mL) 1-azido-2-chloroethane (1.87 g, 17.7 mmol, 1.0 eq) and potassium hydrogen carbonate (3.67 g, 26.5 mmol, 1.5 eq) were added and the suspension was heated to 90 °C for 16 h. Water (100 mL) was added after cooling down to room temperature and the solution was extracted with EtOAc (3 × 100 mL). The combined organic layers were washed with LiCl solution (10%, 2 × 50 mL) and brine (100 mL), dried over $MgSO_4$ and the solvent was removed under reduced pressure. 1-Azidoethyl-3-nitropyrazole (**17**) (2.52 g, 13.8 mmol, 78%) was obtained as a yellow liquid. If desired the crude product can be purified further by flash column chromatography (*i*Hex:EtOAc 8 : 2, R_f = 0.2).

DTA (5 °C min^{-1}) T_{dec} = 214 °C; **^{1}H NMR** (400 MHz, $CDCl_3$, 25 °C): δ (ppm) = 7.55 (d, $^3J_{H,H}$ = 2.5 Hz, 1H), 6.92 (d, $^3J_{H,H}$ = 2.5 Hz, 1H), 4.32 (t, $^3J_{H,H}$ = 5.1 Hz, 2H) (t, $^3J_{H,H}$ = 5.1 Hz, 2H); **^{13}C{^{1}H} NMR** (101 MHz, $CDCl_3$, 25 °C): δ (ppm) = 133.2, 103.2, 52.8, 50.4; **^{14}N{^{1}H} NMR** (29 MHz, $CDCl_3$, 25 °C): δ (ppm) = −21,−135,−169; **EA** ($C_5H_6N_6O_2$, 182.14 g/mol) calc. (found): C 32.97 (33.19), H 3.32 (3.28), 46.14 (46.17) %; **IR** (ATR, rel. int.): $\tilde{\nu}$ (cm^{-1}) = 3157 (w), 3131 (w), 2098 (s), 1542 (s), 1536 (s), 1504 (s), 1456 (m), 1444 (m), 1420 (w), 1382 (s), 1348 (m), 1296 (vs), 1191 (m), 1177 (m), 1053 (m), 999 (m), 939 (vw), 822 (vs), 779 (s), 751 (s), 687 (w), 629 (w), 617 (w), 553 (w), 488 (w), 420 (w).

1-Azidoethyl-4-nitropyrazole (18)

4-Nitropyrazole[S25] (2.00 g, 17.7 mmol, 1.0 eq) was dissolved in DMF (20 mL), 1-azido-2-chloroethane (1.87 g, 17.7 mmol, 1.0 eq) and potassium hydrogen carbonate (3.67 g, 26.5 mmol, 1.5 eq) were added and the suspension was heated to 90 °C for 16 h. Water (100 mL) was added after cooling down to room temperature and the solution was extracted with EtOAc (3 × 100 mL). The combined organic layers were washed with LiCl solution (10%, 2 × 50 mL) and brine (100 mL), dried over $MgSO_4$ and the solvent was removed under reduced pressure. 1-Azidoethyl-4-nitropyrazole (**18**) (2.83 g, 15.5 mmol, 87%) was obtained as yellow liquid. If desired the crude product can be purified further by flash column chromatography (*i*Hex:EtOAc 6 : 4, R_f = 0.34).

DTA (5 °C min^{-1}) T_{dec} = 214 °C; **^{1}H NMR** (400 MHz, $CDCl_3$, 25 °C): δ (ppm) = 8.21 (s, 1H), 8.12 (s, 1H), 4.28 (t, $^3J_{H,H}$ = 5.5 Hz 2H), 3.82 (t, $^3J_{H,H}$ = 5.5 Hz 2H).; **^{13}C{^{1}H} NMR** (101 MHz, $CDCl_3$, 25 °C): δ (ppm) = 136.6, 129.7, 52.6, 50.2; **^{14}N{^{1}H} NMR** (29 MHz, $CDCl_3$, 25 °C): δ (ppm) = −14,−139,−171; **EA** ($C_5H_6N_6O_2$, 182.14 g/mol) calc. (found): C 32.97 (33.37), H 3.32 (3.25), N 46.14 (45.64) %; **IR** (ATR, rel. int.): $\tilde{\nu}$ (cm^{-1}) = 3135 (w), 2935 (vw), 2098 (s), 1529 (s), 1506 (vs), 1475 (m), 1440 (m), 1405 (s), 1371 (w), 1351 (m), 1312 (s), 1296 (vs), 1190 (w), 1170 (w), 1129 (m), 1065 (w), 1002 (m), 971 (w), 945 (w), 874 (m), 817 (vs), 753 (vs), 680 (w), 630 (w), 596 (m), 550 (m), 498 (w), 418 (w).

1-Azidoethyl-3,4-dinitropyrazole (19)

3,4-Dinitropyrazole[S23] (2.00 g, 12.7 mmol, 1.0 eq) was dissolved in DMF (20 mL) and 2-azidoethyl mesylate (2.09 g, 12.7 mmol, 1.0 eq) and potassium hydrogencarbonate (2.62 g, 26.5 mmol, 1.5 eq) were added. The suspension was heated to 90 °C for 16 h. Water (100 mL) was added after cooling down to room temperature and the solution was extracted with EtOAc (3 × 100 mL). The combined organic layers were washed with LiCl solution (10%, 2 × 50 mL) and brine (100 mL), dried over $MgSO_4$ and the solvent was removed under reduced

pressure. 1-Azidoethyl-3,4-dinitropyrazole (**19**) (2.52 g, 13.8 mmol, 78%) was obtained as yellow liquid. If desired the crude product can be purified further by flash column chromatography (*i*Hex:EtOAc 6 : 4, R_f = 0.28).

DTA (5 °C min^{-1}) T_{melt} = 50 °C, T_{dec} = 216 °C; **^{1}H NMR** (400 MHz, $CDCl_3$, 25 °C): δ (ppm) = 8.25 (s, 1H), 4.31 (t, $^{3}J_{H,H}$ = 5.3 Hz, 2H), 3.89 (t, $^{3}J_{H,H}$ = 5.5 Hz, 2H); **^{13}C{^{1}H} NMR** (101 MHz, $CDCl_3$, 25 °C): δ (ppm) = 154.4, 132.1, 130.1, 53.7, 49.6; **^{14}N{^{1}H} NMR** (29 MHz, $CDCl_3$, 25 °C): δ (ppm) = −28,−136; **EA** ($C_5H_5N_7O_4$, 227.14 g/mol) calc. (found): C 26.44 (26.83), H 2.22 (2.25), N 43.17 (42.78) %; **IR** (ATR, rel. int.): $\tilde{\nu}$ (cm^{-1}) = 3136 (m), 2145 (m), 2102 (s), 1552 (s), 1535 (s), 1512 (vs), 1456 (s), 1443 (m), 1432 (m), 1411 (m), 1362 (s), 1336 (s), 1297 (vs), 1228 (m), 1147 (s), 1118 (m), 1064 (w), 1016 (w), 1003 (m), 935 (w), 859 (s), 805 (s), 756 (m), 746 (s), 702 (w), 668 (w), 642 (m), 626 (m), 593 (m), 557 (m), 503 (m), 443 (m).

1-Azidoethyl-3-azido-4-nitropyrazole (20)

To a solution of 1-nitratoethyl-3,4-dinitropyrazole (**6**) (1.50 g, 6.07 mmol, 1.0 eq.) in DMF (75 mL) sodium azide (0.99 g, 15.2 mmol, 2.5 eq.) was added. The reaction mixture was then heated at 80 °C for at least 18 h. Upon cooling to room temperature, the reaction was poured into ice water (~75 mL) and extracted with EtOAc (3 × 80 mL). The combined organic phases were subsequently washed with $NaHCO_3$ (9% aq. 50 mL) and LiCl (10% aq. 70 mL), followed by drying over $MgSO_4$ and evaporation of the solvent to give 1-azidoethyl-3-azido-4-nitropyrazole (**20**) (0.93 g, 4.154 mmol, 68%) as yellow solid. If desired, the obtained compound can be further purified via column chromatography (EtOAc, R_f = 0.8).

DTA (5 °C min^{-1}) T_{melt} = 41 °C, T_{dec} = 159 °C; **^{1}H NMR** (400 MHz, DMSO-*d*6, 25 °C): δ (ppm) = 8.98 (s, 1H), 4.31 (t, $^{3}J_{H,H}$ = 5.5 Hz, 2H), 3.81 (t, $^{3}J_{H,H}$ = 5.5 Hz, 2H); **^{13}C{^{1}H} NMR** (101 MHz, DMSO-*d*6, 25 °C): δ (ppm) = 142.6, 133.8, 124.4, 51.9, 49.2; **^{14}N{^{1}H} NMR** (29 MHz, DMSO-*d*6, 25 °C): δ (ppm) = −18,−133,−142; **EA** ($C_5H_5N_9O_2$, 223.16 g/mol) calc. (found): C 26.91 (27.19), H 2.26 (2.67), N 56.49 (56.24) %; **IR** (ATR, rel. int.): $\tilde{\nu}$ (cm^{-1}) = 3116 (m), 3015 (vw), 2941 (w), 2874 (w), 2341 (w), 2232 (w), 2136 (vs), 2094 (vs), 1732 (w), 1532 (s), 1505 (vs), 1480 (s), 1471 (s), 1449 (m), 1435 (s), 1396 (m), 1381 (s), 1356 (s), 1343 (s), 1303 (vs), 1224 (vs), 1186 (s), 1148 (s), 1069 (m), 991 (m), 937 (w), 869 (s), 838 (m), 785 (m), 755 (s), 678 (m), 639 (s), 612 (s), 550 (w), 532 (m), 505 (m), 498 (s), 415 (m).

11. References

[S1] CrysAlisPro, Oxford Diffraction Ltd. version 171.33.41, **2009**.

[S2] G. M. Sheldrick, *Acta Cryst.*, **2015**, A71, 3–8.

[S3] Dolomanov, O. V., Bourhis, L. J., Gildea, R. J., Howard, J. A. K. & Puschmann, H. J., *Appl. Cryst.*, **2009**, 42, 339–341.

[S4] SCALE3 ABSPACK – An Oxford Diffraction program (1.0.4, gui: 1.0.3), Oxford Diffraction Ltd., **2005**.

[S5] APEX3. Bruker AXS Inc., Madison, Wisconsin, USA.

[S6] M. J. Frisch, G. W. Trucks, H. B. Schlegel, G. E. Scuseria, M. A. Robb, J. R. Cheeseman, G. Scalmani, V. Barone, G. A. Petersson, H. Nakatsuji, X. Li, M. Caricato, A. V. Marenich, J. Bloino, B. G. Janesko, R. Gomperts, B. Mennucci, H. P. Hratchian, J. V. Ortiz, A. F. Izmaylov, J. L. Sonnenberg, D. Williams-Young, F. Ding, F. Lipparini, F. Egidi, J. Goings, B. Peng, A. Petrone, T. Henderson, D. Ranasinghe, V. G. Zakrzewski, J. Gao, N. Rega, G. Zheng, W. Liang, M. Hada, M. Ehara, K. Toyota, R. Fukuda, J. Hasegawa, M. Ishida, T. Nakajima, Y. Honda, O. Kitao, H. Nakai, T. Vreven, K. Throssell, J. A. Montgomery, Jr., J. E. Peralta, F. Ogliaro, M. J. Bearpark, J. J. Heyd, E. N. Brothers, K. N. Kudin, V. N. Staroverov, T. A. Keith, R. Kobayashi, J. Normand, K. Raghavachari, A. P. Rendell, J. C. Burant, S. S. Iyengar, J. Tomasi, M. Cossi, J. M. Millam, M. Klene, C. Adamo, R. Cammi, J. W. Ochterski, R. L. Martin, K. Morokuma, O. Farkas, J. B. Foresman, and D. J. Fox, Gaussian16 ,Gaussian, Inc., Wallingford, CT, USA, **2016**.

[S7] a) J. W. Ochterski, G. A. Petersson, J. A. Montgomery, *J. Chem. Phys*. **1996**, *104*, 2598–2619; b) J. A. Montgomery, M. J. Frisch, J. W. Ochterski, G. A. Petersson, *J. Chem. Phys*. **2000**, *12*, 6532–6542.

[S8] a) L. A. Curtiss, K. Raghavachari, P. C. Redfern, J. A. Pople, *J. Chem. Phys*. **1997**, *106*, 1063–1079; b) B. M. Rice, S. V. Pai, J. Hare, *Combust. Flame* **1999**, *118*, 445–458; c) E. F. C. Byrd, B. M. Rice, *J. Phys. Chem. A* **2006**, *110*, 1005–1013.

[S9] P. J. Linstrom, W. G. Mallard (Editors), *National Institute of Standards and Technology Standard Reference Database Number 69*, Gaithersburg MD, 20899, http://webbook.nist.gov/chemistry/ (July **2022**).

[S10] S. Wahler, P. Chung, T. M. Klapoetke, **2023**, Training machine learning models based on the structural formula for the enthalpy of vaporization and sublimation and a thorough analysis of Trouton's rules *J. Energ. Mater* doi.org/10.1080/07370652.2023.2219678.

[S11] NATO standardization agreement (STANGAG), *Chemical compatibility of ammunition components with explosives (non-nuclear application*), No. 4147 2. ed., **2001**.

[S12] R. Meyer, J. Koehler, A. Homburg, *Explosives*, 6th edn., Wiley, Weinheim, **2007**, p. 196–198.

[S13] R. Meyer, J. Koehler, A. Homburg, *Explosive*s, 6th edn., Wiley, Weinheim, **2007**, p. 145.

[S14] L. Bauer, M. Benz, T. M. Klapoetke, *Propellants Explos. Pyrotech*. **2022**, *47*, e202100332.

[S15] http://www.bam.de (July **2022**).

[S16] NATO Standardization Agreement (STANAG) on Explosives, *Impact Sensitivity Tests*, no. 4489, 1st ed., September 17, **1999**.

[S17] WIWEB-Standardarbeitsanweisung 4-5.1.02, Ermittlung der Explosionsgefährlichkeit, hier der Schlagempfindlichkeit mit dem Fallhammer, November 8, **2002**.

[S18] NATO Standardization Agreement (STANAG) on Explosives, *Friction Sensitivity Tests*, no. 4487, 1st ed., August 22, **2002**.

[S19] WIWEB-Standardarbeitsanweisung 4-5.1.03, Ermittlung der Explosionsgefährlichkeit oder der Reibeempfindlichkeit mit dem Reibeapparat, November 8, **2002**.

[S20] OZM, http://www.ozm.cz. (July **2022**).

[S21] Impact: Insensitive >40 J, less sensitive ≤35 J, sensitive ≤4 J, very sensitive ≤3 J; friction: Insensitive >360 N, less sensitive=360 N, sensitive 80 N, very sensitive ≤80 N, extreme sensitive ≤10 N; According to the UN Recommendations on the Transport of Dangerous Goods (+) indicates: not safe for transport.

[S22] W. Meng, P. T. W. Cheng, Patent WO 2011/130459 A1, **2011**.

[S23] M. F. Boelter, A. Harter, T. M. Klapoetke, J. Stierstorfer *ChemPlusChem* **2018**, *83*, 804–811.

[S24] M. Treu, S. K. Zahn, Patent WO 2016/180843 A1, **2016**.

[S25] R. S. Gross, Z. Guo, B. Dyck, T. Coon, C. Q. Huang, R. F. Lowe, D. Marinkovic, A. Moorjani, J. Nelson, S. Zamani-Kord, D. E. Grigoriadis, S. R. J. Hoare, P. D. Crowe, J. H. Bu, M. Haddach, J. McCarthy, J. Saunderes, R. Sullivan, T. Cheng, J. P. Williams, *J. Med. Chem.* **2005**, *48*, 5780–5793.

[S26] P. J. Machin, Patent EP0034831A2, **1981**.
